# Decoding MnO_2_ redox chemistry from mechanistic ambiguity to design principles for aqueous Zn-ion batteries

**DOI:** 10.1038/s41467-026-74350-z

**Published:** 2026-06-11

**Authors:** Yuan Shang, Sankhadip Saha, Haotian Wen, Qihui Zhang, Xinyuan Wu, Bram Hoex, Mingyue Wang, Nana Wang, Tongjun Luo, Sougat Purohit, Gopalakrishnan Sai Gautam, Wesley M. Dose, Lars Thomsen, Shery Chang, Priyank Kumar, Dipan Kundu

**Affiliations:** 1 LBRI, School of Chemical Engineering, UNSW Sydney, Kensington, Australia; 2 School of Materials Science and Engineering, UNSW Sydney, Kensington, Australia; 3 School of Photovoltaic and Renewable Energy Engineering, UNSW Sydney, Kensington, Australia; 4 Institute for Superconducting and Electronic Materials, Faculty of Engineering and Information Sciences, University of Wollongong, Wollongong, Australia; 5 Centre for Clean Energy Technology, School of Mathematical and Physical Sciences, Faculty of Science, University of Technology Sydney, Sydney, Australia; 6 School of Chemistry, The University of Sydney, Sydney, Australia; 7 Department of Materials Engineering, Indian Institute of Science, Bengaluru, India; 8 Australian Synchrotron, ANSTO, Clayton, VIC Australia; 9 School of Chemical Engineering, UNSW Sydney, Kensington, Australia

**Keywords:** Batteries, Batteries

## Abstract

Manganese dioxide (MnO_2_) is a leading positive electrode candidate for aqueous zinc-ion batteries, combining safety, high voltage, low cost, and sustainability for grid-scale storage. However, its practical development remains restricted by poor reversibility, rooted in an unresolved mechanistic debate spanning over a decade. Here, we combine operando characterizations, multimodal spectroscopic analyses, and theory to establish a unified picture: proton-primed MnO_2_ dissolution and subsequent redeposition as nanocrystalline and disordered MnO_x_ nanosheets, coexisting with reversible proton intercalation in parent MnO_2_ and predominantly in deposited MnO_x_, forming a dual redox mechanism. pH-driven insulating byproduct precipitation emerges as a significant kinetic barrier that limits deep dissolution and capacity utilization. Guided by these insights, we introduce surface activation and architectural design strategies toward mitigating kinetic barriers, enabling enhanced capacity and stability in both Swagelok and pouch-type cells. By reconciling mechanistic ambiguity and translating it into actionable design principles, this work demonstrates a framework for developing durable Mn-based positive electrodes for sustainable energy storage.

## Abstract (web summary)

The redox chemistry of manganese dioxide cathodes in aqueous zinc-ion batteries remains debated despite extensive study. Here, authors clarify the coupled dissolution–redeposition and proton redox processes and show how this mechanistic insight informs electrode design for stable aqueous batteries.

## Introduction

Aqueous zinc-ion batteries (AZIBs) are emerging as safe, low-cost candidates for grid-scale energy storage, but their practical development rests on advancing both zinc negative electrode and positive electrode (electro)chemistries under scalable conditions^1–3^. While significant progress has been made toward stabilizing zinc negative electrodes—through electrolyte engineering^4–6^, surface coatings^7,8^, and dendrite suppression^9–11^—positive electrode development has lagged behind. Among available options, manganese dioxide (MnO_2_) stands out as the most promising positive electrode material. It offers a high theoretical capacity of 308 mAh g^−1^, typically achievable in mildly acidic aqueous electrolytes of AZIBs, moderate operating voltage (~1.3–1.4 V vs Zn/Zn^2+^), and is low-cost and sustainable. The working potential (~0.8 to 1.8 V vs Zn/Zn^2+^) sits comfortably within the stability limit of aqueous electrolytes, mitigating oxygen evolution and hydrogen evolution reaction (OER and HER) risks that can pose a challenge at higher and lower potentials, respectively. With targeted materials-level optimization and voltage tuning, MnO_2_-based positive electrodes could deliver high energy density without compromising electrolyte stability—an advantage that layered vanadium oxides and Prussian blue analogues (PBAs) fail to offer^12^. Vanadium oxide-based AZIB positive electrodes, despite their high capacities, suffer from a lower average potential (~0.5 V below MnO_2_), which will complicate cell-to-pack engineering and significantly reduce energy density^13^. PBAs, on the other hand, provide higher working potentials (1.7–1.75 V vs. Zn/Zn^2+^) but exhibit low capacities and poor stability at practical rates; their high operating voltage also makes them vulnerable to OER, particularly under low-current cycling where coulombic efficiency drops below 98% and stability deteriorates^14^. These limitations position MnO_2_ as the most balanced positive electrode candidate for AZIBs^13^.

**Fig. 1:**
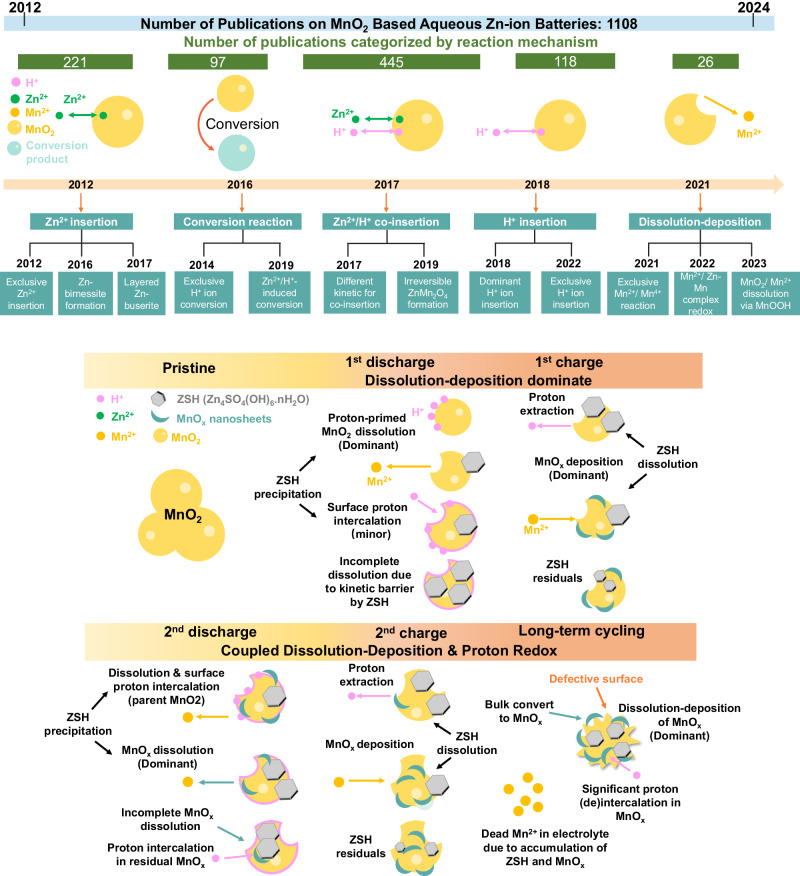
Evolution and unification of MnO_2_ redox mechanisms. Evolution of mechanistic understanding of MnO_2_ positive electrodes in aqueous Zn-ion batteries (top) and the unified mechanistic picture revealed in this work (bottom).

Yet, despite MnO_2_’s promise, its electrochemistry in AZIBs remains one of the most debated topics in aqueous battery research, as illustrated by the evolving hypotheses in Fig. 1 (top panel). Over the past decade, mechanistic models have shifted from Zn^2+^ tunnel insertion^15^ to conversion reactions involving Zn^2+^ intercalation^16,17^, then to proton-coupled conversion steps (MnO_2_ → MnOOH)^18^, to Zn_x_MnO_2_ formation^19^, followed by Zn^2+^/H^+^ co-insertion^20,21^ and proton insertion^22,23^, before recent studies^24–26^ proposed dissolution–deposition as a dominant process. Our analysis of more than 1,100 publications from the past ten years reveals that these models have not converged: Zn^2+^ insertion and co-insertion still dominate the literature even in recent years (Fig. S1), while only a small fraction acknowledge dissolution–deposition, despite growing experimental evidence. This fragmentation is not merely academic; it leaves fundamental questions unresolved. Why does MnO_2_ deliver far less than its theoretical capacity under practical loadings? Why does performance deteriorate over extended cycling? And how do parasitic processes such as ZSH (zinc sulfate hydroxide; Zn_4_SO_4_(OH)_6_·xH_2_O) precipitation, triggered by interfacial pH shifts, influence reversibility? Existing frameworks, often lattice-centric, fail to capture the dynamic interplay of interfacial reactions, dissolution, redeposition, and proton involvement across cycles. Addressing these gaps is critical to establish design principles that translate MnO_2_’s theoretical advantages into durable, high-capacity positive electrodes for grid-scale storage.

In this work, we establish a unified mechanistic picture of MnO_2_ electrochemistry in AZIBs (Fig. 1) by integrating operando characterization, in-depth spectroscopic analyses, and density functional theory modeling. Our findings reveal that charge storage in MnO_2_ is governed by a proton-primed dissolution–deposition pathway, wherein surface protonation destabilizes Mn–O bonds and initiates Mn^2+^ dissolution, followed by redeposition as disordered MnO_x_ nanosheets. This process coexists with reversible proton intercalation in both the parent MnO_2_ and the deposited MnO_x_, forming a dual redox mechanism. We further identify ZSH precipitation as a dominant kinetic barrier that limits deep dissolution and capacity utilization. Guided by these insights, we demonstrate that surface and architectural engineering can mitigate these bottlenecks, enhancing capacity, reversibility, and long-term cycling stability. By reconciling long-standing mechanistic ambiguities, this study provides actionable design principles for Mn-based positive electrodes, bridging the gap between fundamental understanding and practical performance in AZIBs.

## Results and discussion

### The initial mechanistic transition

**Fig. 2:**
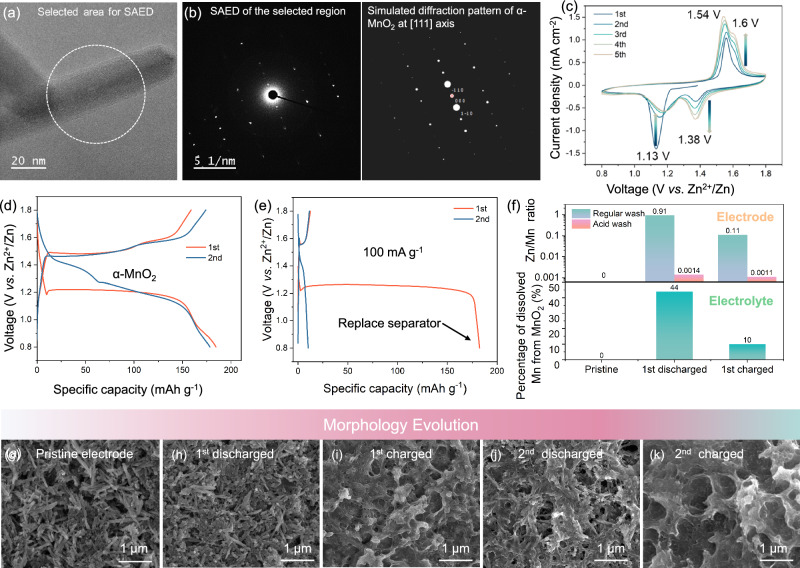
Electrochemistry of α-MnO_2_ during the initial cycle. **a** Representative TEM image of the α-MnO_2_ nanorod with the region indicated for SAED analysis, and **b** experimental and simulated SAED pattern for α-MnO_2_ nanorod. **c** The CV curves of the Zn||MnO_2_ cell during the first five cycles at a scan rate of 0.2 mV s^−1^ in the 0.8 and 1.8 V window vs. Zn^2+^/Zn. **d** The GCD profile of the Zn||MnO_2_ cell for the first two cycles when cycling at 100 mA g^−1^ in the same window. **e** The GCD profile of the Zn||MnO_2_ cell for the first two cycles before and after replacing the separator. **f** The Zn/Mn ratio for the MnO_2_ electrode before and after cycling, and the percentage of dissolved Mn (relative to MnO_2_ mass in the pristine electrode) in the electrolyte measured by ICP-OES after first discharge and charge. **g**–**k** The SEM images of the α-MnO_2_ electrode at different cycling stages.

Tunneled MnO_2_, with 1 × 1 and 2 × 2 tunnels, is the most widely used positive electrode for rechargeable zinc batteries with mildly acidic aqueous sulfate electrolytes owing to its structural stability and moderate electronic conductivity^27^. Therefore, here, α-MnO_2_ is selected as a model system to investigate the charge storage mechanism of MnO_2_ in AZIBs. Hydrothermal synthesis yielded phase-pure α-MnO_2_ nanorods with lengths of 0.1 to 1 μm and widths of 20 to 50 nm as revealed by transmission electron microscopy (TEM) and selected area electron diffraction (SAED, Fig. 2a, b) and additional X-ray and electron microscopy analysis (Figs. S2, S3). Electrochemical activation in a 1 M ZnSO_4_ electrolyte without Mn^2+^ additive reveals striking evolutions. The first cycle exhibits a single discharge plateau and two cyclic voltammetry (CV) peaks, at 1.13 V and 1.54 V, corresponding to cathodic and anodic processes, respectively, but subsequent cycles develop a dual plateau profile with new CV features during reduction (1.38 V) and oxidation (1.60 V) (Fig. 2c, d, Fig. S4). This transition persists thereafter, signaling a fundamental change in the dominant redox process after the first cycle. From an initial specific capacity of 184 mAh g^−1^ at 100 mA g^−1^ ( ~ C/3 current rate) there is gradual fading during subsequent cycles, retaining only 100 mAh g^−1^ after 100 cycles (Fig. S5). Conventional explanations^18,28^ invoke Jahn-Teller distortion and Mn^3+^ disproportionation, but these lattice-centric models contradict emerging evidence for a dissolution-deposition pathway. Resolving this ambiguity is critical because design strategies will diverge; stabilizing the lattice may be irrelevant or even detrimental to obtaining higher capacity if interfacial chemistry governs reversibility.

### Evidence for dissolution–deposition as the governing mechanism during initial cycles

To probe the governing mechanism, we interrupted cycling after the first discharge and replaced the separator and electrolyte. The subsequent charge capacity collapsed to ~12 mAh g^−1^ and did not recover (Fig. 2e, Fig. S6). If Zn^2+^ intercalation were dominant, the charge carriers would remain in the lattice and still be extractable after electrolyte replacement. The near-complete loss of capacity instead implicates a solution-mediated pathway: Mn dissolves during discharge and is removed when the electrolyte is replaced, preventing redeposition. Quantitative ICP-OES (inductively coupled plasma optical emission spectroscopy) confirms this picture, showing that 44% of the active Mn (w.r.t. the Mn in the pristine MnO_2_ electrode) dissolves during the first discharge (Fig. 2f). Concurrently, ZSH precipitates at the interface driven by H^+^ removal or OH^−^ generation during proton consumption by MnO_2_ dissolution or possible H^+^ intercalation. To probe Zn^2+^ intercalation further, the impact of two cleaning treatments was compared for the discharged electrode. Quantitative analysis after regular washing (rinsing with deionized water or DI water and ethanol) shows a high Zn to Mn ratio of 0.91, while dilute acid washing (0.1 M H_2_SO_4_ + DI water + ethanol) that removes ZSH, yields a negligible Zn to Mn ratio of 0.0014 (Fig. 2f). If Zn^2+^ intercalation or Zn^2+^ intercalation mediated conversion of MnO_2_ to any Zn_x_Mn_2_O_4_ kind of phase were significant, the Zn/Mn ratio would remain high even after dilute acid washing. Its near-zero value excludes Zn^2+^ intercalation or any Zn-mediated conversion as a dominant mechanism for MnO_2_. During charging, ZSH partially dissolves as protons are released by MnO_2_ deposition, reducing the Zn/Mn ratio in the regularly washed charged samples. However, residual ZSH persists, suggesting incomplete dissolution during charging. ICP-OES analysis of the electrolyte corroborates this mechanism. Mn^2+^ concentration spikes in the electrolyte after discharge (44% of MnO_2_, Fig. 2f) and decreases upon charging as Mn redeposits. A trace amount of Mn^2+^ lingers in the electrolyte, consistent with the inferior Coulombic efficiency (CE, Fig. S7) in the first cycle.

Morphological evolution supports incomplete dissolution. SEM (scanning electron microscopy) shows that after the first discharge, α-MnO_2_ nanorods remain (Fig. 2g, h), contradicting claims of complete dissolution in previous studies^29^. This suggests that the dissolution reaction does not consume all active α-MnO_2_ particles, thereby explaining the capacity shortfall relative to the two-electron theoretical limit (616 mAh g^−1^). Upon charging, the electrode surface becomes uniformly covered with newly formed nanosheets (Fig. 2i), similar to observations in the previous studies^23,30^, which identified them as irreversible nanograins. These nanosheets mostly dissolve during the second discharge, thereby revealing the underlying nanorod structure of α-MnO_2_ (Fig. 2j), and reemerge upon charging (Fig. 2k), confirming a reversible deposition-dissolution cycle that persists over extended cycling (Fig. S8). This is further validated in a MnO_2_-free cell with MnSO_4_-containing electrolyte (see discussion for Figs. S9−11). The SEM-EDS (EDS: Energy dispersive X-ray spectroscopy) mapping, as presented in Figs. S12, 13, further reveals uniformly distributed Mn and O across the deposited nanosheets, with minimal Zn, indicating the deposit primarily consists of manganese-based oxides (MnO_x_).

**Fig. 3:**
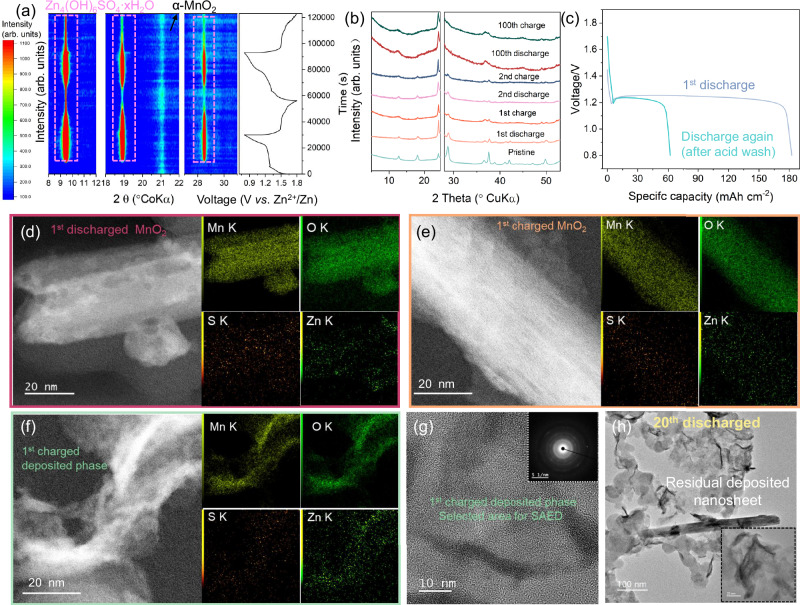
Structural and morphological signatures revealing dominant dissolution-deposition. **a** Operando XRD evolution for ZSH and α-MnO_2_ when cycled at 30 mA g^−1^, along with the corresponding GCD profile. **b** The ex-situ XRD pattern for the α-MnO_2_ electrode after different discharge and charge at a current of 100 mA g^−1^. **c** The GCD profile of discharge before and after acid washing and reassembling. STEM-EDS analysis for α-MnO_2_ electrode after **d** 1^st^ discharge and **e** 1^st^ charge to show the element distribution among the nanorod. **f** STEM-EDS analysis reveals the element composition of the newly deposited phase. STEM–EDS mapping shows uniform Mn and O distribution with negligible Zn, confirming the absence of Zn incorporation. **g** TEM image for the newly deposited phase and corresponding SAED pattern. **h** TEM image of the 20^th^ discharged α-MnO_2_ electrode. Inset: high-magnification view of the residual MnO_x_ nanosheets.

Spectroscopic and structural evidence further complements the dissolution–deposition mechanism. Raman spectra show that the 620 cm^−1^ Mn-O stretch weakens on discharge and recovers on charge across cycles (Fig. S14), without vanishing after discharge, consistent with incomplete dissolution. Operando X-ray diffraction (XRD) reveals ZSH peaks (at 9.5°, 18.9°, and 28.5°) emerging and intensifying during discharge and diminishing upon charging above ~1.5 V (Fig. 3a), while α-MnO_2_ reflections persist with essentially constant d-spacings, and no additional crystalline phases appear beyond ZSH during cycling, as further confirmed by operando Synchrotron XRD analysis (Fig. S15). Synchrotron XRD, with superior resolution compared to laboratory XRD, further affirms that the deposited MnO_x_ is either amorphous or highly disordered, and the mechanism excludes conversion pathways to ZnMn_2_O_4_ or ZnMn_3_O_7_. Even after extended cycling, α-MnO_2_ displays its structural identity (Fig. 3b), albeit with a gradual loss of crystallinity due to progressive transformation into amorphous or disordered MnO_x_. Although the discharge plateau shifts between the first and second cycles, the operando XRD signatures remain effectively unchanged, indicating a consistent dissolution–deposition mechanism. The voltage shift is better attributed to the progressive dissolution/redeposition of MnO_x_ with oxidation states distinct from the parent MnO_2_ (vide infra). Notably, ZSH precipitated during discharge is difficult to remove fully without rigorous cleaning (Fig. S16). Any residual ZSH can significantly compromise XRD interpretation because, upon drying, ZSH exhibits hydration-dependent peak shifts, which may lead to misassigning its reflections to other phases^31^.

### Interfacial passivation as a significant kinetic barrier to deep-dissolution of α-MnO_2_

Although the dissolution-deposition mechanism of MnO_2_ is now relatively well established, an important question remains: why does the specific capacity of α-MnO_2_ (only ~182 mAh g^−1^) fall well below the two-electron theoretical capacity for dissolution reaction (616 mAh g^−1^)? Because the dissolution–deposition pathway is surface-mediated, the accessibility of electrochemically active surface area is expected to strongly influence the overall extent of reaction. Operando XRD (Fig. 3a) shows substantial formation of ZSH during discharge, which only partially dissolves upon charging. Given its insulating nature, ZSH contributes to interfacial passivation and restricts access to reactive Mn–O sites, thereby slowing dissolution–deposition kinetics. Consistent with this, acid washing to remove ZSH from the discharged electrode yields an additional 62 mAh g^−1^ in the following discharge (Fig. 3c), confirming that ZSH surface deposits impose a substantial kinetic barrier. Notably, ZSH can also transform into other insulating byproducts, such as poorly ordered ZnO^32^, which is difficult to resolve unambiguously by XRD or other spectroscopic techniques; such species would also contribute to interfacial passivation. Beyond surface accessibility, the capacity limitation may also stem from the local atomic structure in MnO_2_, as has been proposed earlier^32^. In particular, electron transfer is more favorable in edge-sharing than corner-sharing Mn–Mn sites. Preferential removal of edge-sharing octahedra during dissolution enriches corner-sharing Mn units at the surface, ultimately suppressing further dissolution and available capacity.

### Electron microscopy evaluation rules out the possibility of a conversion reaction

Although the above results strongly support the dissolution-deposition pathway and exclude Zn^2+^ mediated conversion or intercalation as dominant mechanisms, the amorphous or disordered nature of the deposited phase precludes definitive identification by XRD alone. High-resolution electron microscopy was employed to resolve this. Scanning tunneling electron microscopy-EDS (STEM-EDS) mapping of a single MnO_2_ nanorod (Fig. 3d) after discharge shows uniform Mn and O distribution with no detectable Zn signal (Fig. S17). In addition, consistent with previous analyses, SAED (Fig. S18a) and TEM imaging of the discharged MnO_2_, after ZSH removal by acid treatment, verify that the MnO_2_ retains its tetragonal crystal structure and morphological feature, without the appearance of any other phases (Fig. S18b). Similarly, the charged MnO_2_ nanorod exhibits negligible Zn and S signals (Fig. 3e, Fig. S19) and maintains its crystalline structure (Fig. S20a). The wave-like nanosheets appear during the charging stage and adhere to the MnO_2_ nanorod (Fig. S20b), and STEM-EDS mapping (Fig. 3f, Fig. S21) and SAED analysis (Fig. 3g) of the nanosheet corroborate that the deposited MnO_x_ phase is nanocrystalline or highly disordered, consistent with its invisibility in XRD due to nanoscale dimensions. Across extended cycling (2^nd^ and even 20^th^ cycle), no evidence of phase transition (Fig. S22) or Zn^2+^ intercalation (Figs. S23–27) emerges, with MnO_2_ nanorod consistently retaining its original crystal structure. Interestingly, TEM imaging and STEM-EDS (Fig. 3h, Fig. S26) show that some deposited nanosheets persist after discharge (20^th^ discharge), coexisting with MnO_2_ nanorods. Contrary to previous claims^33^, the reversibly deposited phase does not change identity after long-term cycling, and remains as MnO_x_ **(**Figs. S26, 27). Collectively, direct microscopic evidence unequivocally rules out Zn^2+^-mediated insertion or conversion reaction and confirms that the deposited product is nanocrystalline and disordered MnO_x_.

### Proton-coupled surface redox: coexisting with dissolution–deposition

**Fig. 4:**
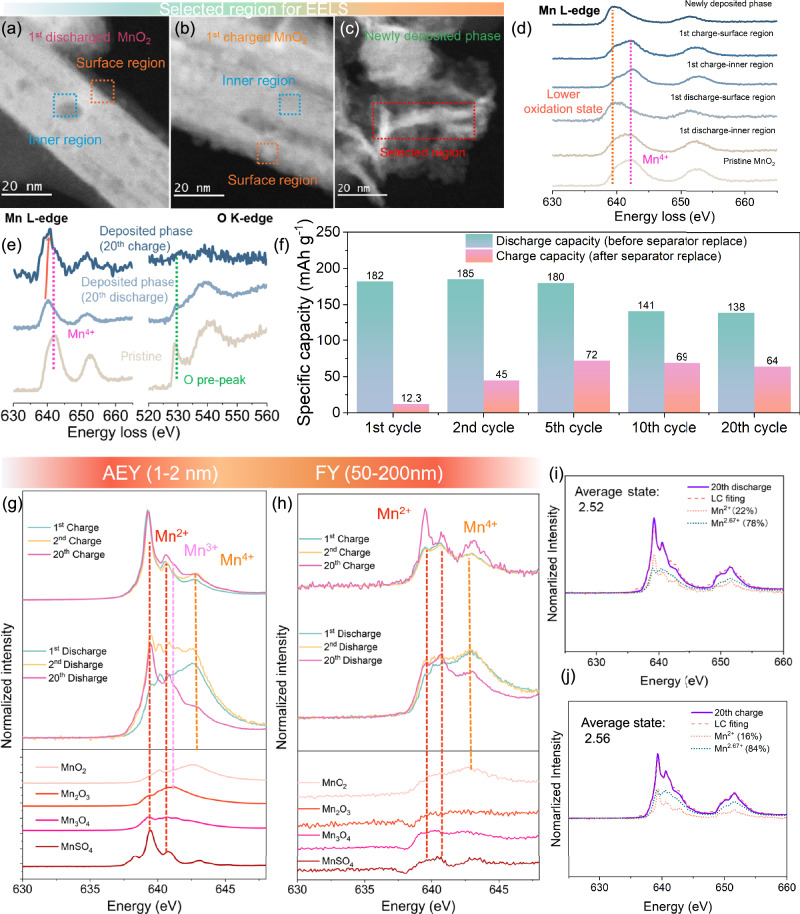
Valence evolution confirms dual redox. The high-angle annular dark-field scanning TEM (HAADF-STEM) images selected from **a** 1^st^ discharge α-MnO_2_ nanorod, **b** 1^st^ charge α-MnO_2_ nanorod and **c** newly deposited phase for EELS measurement. **d** The corresponding EELS Mn L-edge spectra collected from the labeled region for the electrodes. **e** Comparison of EELS Mn L-edge and O K-edge spectra for the pristine electrode and 20^th^ cycled deposited phase. **f** The evolution of recharged specific capacity after replacing the separator and reassembling the cell at different cycles. The soft XAS Mn L-edge spectra collected from **g** AEY and **h** FY mode for different discharge and charge electrodes. The linear combination fitting of AEY Mn L-edge spectra for the 20^th^ **i** discharged and **j** charged electrode. All the spectra are normalized to pre-edge baseline (set to 0) and post-edge continuum (set to 1).

Disentangling proton intercalation from MnO_2_ dissolution remains challenging because both processes—MnO_2_ dissolution (Eq. (2)) and H⁺ intercalation (Equation 3)—raise the local pH at the electrode/electrolyte interface and promote ZSH formation during discharge. To rigorously evaluate the H⁺ insertion hypothesis, we probed Mn valence states in pristine and cycled MnO_2_, and the deposited MnOₓ phase using electron energy-loss spectroscopy (EELS) (Fig. 4a–c, Fig. S28). The EELS Mn L-edge spectrum of the pristine α-MnO_2_ shows an L_3_ edge at 642.1 eV and an L_2_ edge at 652.6 eV, consistent with Mn^4+^ valence state across the nanorod (Fig. 4d)^34^. Strikingly, spectra from the first discharged nanorod (Fig. 4a) reveal distinct coordination environments between the surface and bulk (Fig. 4d). The inner region of the nanorod retains the L_3_-edge peak position and L_3_–L_2_ energy gap (ΔE ≈ 10.5 eV) similar to the pristine phase (Fig. 4d, Table S1), indicating that the bulk remains predominantly Mn^4+^ after discharge. In contrast, the surface region shows a negatively shifted L_3_ edge at 640.0 eV and an increased ΔE of 12.0 eV, signifying a reduction to a lower oxidation state relative to the bulk^35^.

The intensity of the pre-peak in the O K-edge, arising from hybridization between Mn 3 *d* and O 2*p* orbitals, also serves as a sensitive probe of Mn oxidation state and local coordination. A reduction in Mn valency and/or the presence of oxygen vacancies diminishes the O coordination shell and increases electron occupancy in Mn 3 *d* orbitals, thereby attenuating the pre-peak intensity^36,37^. Accordingly, the markedly weaker O K-edge pre-peak (Fig. S29) for the surface region of the α-MnO_2_ nanorods after first discharge, compared to the bulk region and pristine α-MnO_2_ nanorods, further confirms surface reduction. Upon charging, the Mn L_3_ edge of the nanorod surface shifts back to 641.9 eV with a reduced ΔE of 10.6 eV (Fig. 4d, Table S1). In addition, concurrent restoration of the O K-edge pre-peak intensity (Fig. S29) suggests that the surface state change is reversible upon charging. Given the exclusion of Zn^2+^ intercalation based on prior analyses, this reversible surface-limited redox of the α-MnO_2_ can be ascribed to reversible proton intercalation. Notably, a recent study has also confirmed the proton insertion through ^2^H solid-state NMR (nuclear magnetic resonance) spectroscopy and Electrochemical Quartz Crystal Microbalance Studies^38^. However, as we observe, only a very small fraction of the first-discharge capacity arises from the non-dissolution pathway (vide supra), indicating that H^+^ insertion in the parent α-MnO_2_ is limited and predominantly surface-mediated, rather than occurring as bulk intercalation as proposed in that study. Nevertheless, this reversible surface redox persists across extended cycling (2nd and 20th cycles), as evidenced by consistent EELS Mn L_3_-edge shifts and O K-edge pre-peak evolution (Figs. S30, 31, Tables S2, 3).

EELS spectra from the disordered MnO_x_ nanosheet deposited after the first charge reveal a downshifted Mn L_3_-edge (639.8 eV, Fig. 4d, Table S1) and diminished O K-edge pre-peak intensity, consistent with its defective nature and lower average Mn oxidation state. As expected, the MnO_x_ shows a very small change in the O K-edge pre-peak upon discharge and charge owing to its abundant oxygen defects, which already weaken the pre-peak. However, it shows a reversible shift at the Mn L-edge—as evident from the EELS after 20^th^ discharge and charge (Fig. 4e, Table S3)—confirming a reversible redox process attributable to proton (de)intercalation. In fact, the residual MnO_x_ nanosheets, resulting from incomplete dissolution during discharge, become active hosts for proton intercalation. To further confirm the extent of proton intercalation, separator replacement experiments were conducted at various discharge stages (Fig. 4f, Fig. S32). Unlike the first cycle, where the charge capacity collapses post-replacement, subsequent cycles show notable recovery: 45 mAh g^−1^ after the second cycle and stabilization around 70 mAh g^−1^ after the fifth cycle (~40% relative to the first discharge capacity). This recovery confirms that the charge storage mechanism of α-MnO_2_ is not solely dependent on the dissolution/deposition pathway beyond the first cycle. Instead, the continuous buildup of deposited MnO_x_ with repeated cycling contributes to the gradual rebound in charge capacity over successive cycles due to promoted proton intercalation in the residual nanosheets.

### Valence evolution of deposited MnO_x_

While the dissolution–deposition pathway is being increasingly recognized, most studies^39–42^ interpret the charging process predominantly within a Mn^2+^ → Mn^4+^ oxidation framework, often assuming the redeposited phase to be Mn^4+^-rich (e.g., birnessite-MnO_2_^40^ or ZnMn_3_O_7_^42^). The oxidation state of the deposited Mn species remains challenging to establish unambiguously, as commonly used structure-sensitive or indirect probes do not uniquely resolve the valence state of the deposited products^43^. To identify the chemical state of deposited MnO_x_, we employed Mn L-edge soft X-ray absorption spectroscopy (XAS), which probes localized Mn 3*d* states via 2p–3*d* transitions^44^. Unlike Mn K-edge XAS, the L-edge offers high sensitivity to local atomic and electronic structure, enabling direct comparison with reference spectra for distinct oxidation states (Fig. 4g–j)^45,46^. Surface-sensitive Auger electron yield (AEY, probing depth 1–2 nm) spectra of pristine α-MnO₂ electrodes display the expected L_3_ doublet at 639.9 and 642.4 eV (Fig. S33), consistent with Mn^4+^^45,47^. After the first discharge, α-MnO₂ largely retains the Mn^4+^ state (average valence 3.49 by linear combination fitting, Fig. S34a), with minor features at 639.4 and 641.2 eV attributable to surface Mn^2+^ and Mn^3+^, respectively, consistent with proton-mediated surface reduction (Fig. 4g). In stark contrast, after the first charge, the AEY spectrum is dominated by Mn^2+^ and Mn^3+^ features (Fig. 4g), yielding an average oxidation state of +2.28 (Fig. S34b). After 20 cycles, the Mn L-edge spectra become comparable between discharged and charged states (Fig. 4i, j), reflecting the progressive accumulation of MnO_x_, resulting from its incomplete dissolution during discharge - a phenomenon corroborated by both XRD and TEM analyses (vide supra). The discharged sample (Fig. 4i), however, shows a lower Mn oxidation state than the charged sample (Fig. 4j), confirming proton intercalation-mediated redox. Given the surface sensitivity of AEY, the oxidation state (+2.56; charged phase) predominantly reflects the MnO_x_ deposits, closely matching the mixed-valence state of Mn_3_O_4_ (+2.67). In total electron yield (TEY) mode, which probes deeper (5–10 nm), the L_3_ peak evolution mirrors the AEY trend (Fig. S35), with Mn^2+^ contributions to the MnO_x_ signature increasing upon discharge and decreasing upon charge. These observations are in strong agreement with EELS results, reinforcing that surface nanosheet deposition during charge and its progressive accumulation with cycling—undergoing proton intercalation-mediated redox—govern the observed valence evolution.

### Deterioration of dissolution-deposition reversibility: a key contributor to capacity fading

Fluorescence yield (FY) XAS, which penetrates approximately 50-200 nm into the electrode, probes the bulk electronic structure beyond the surface^48^. After the first and the second discharge, the FY spectra exhibit minimal low-valence features, indicating that proton intercalation is confined to the near-surface region and that the α-MnO₂ bulk largely retains its Mn^4+^ state (Fig. 4h). In contrast, the charged electrodes already display mixed-valence characteristics (Mn^2+^/Mn^4+^), reflecting the presence of MnO_x_ deposits on the surface. With progressive cycling, the parent α-MnO_2_ continues to dissolve, even as the capacity becomes increasingly dominated by redox reactions of MnO_x_, leading to a gradual depletion of the parent MnO_2_ phase. Consistent with this, extended cycling (20 cycles, Fig. 4h) leads to prominent low-valence features in both charged and discharged states - more pronounced after charge—indicating a progressive transformation of the crystalline electrode into disordered MnO_x_ and its accumulation on the surface. This underscores a limitation of the dissolution–deposition electrochemistry: it is not fully reversible. Complementary ICP-OES analysis (Fig. S36) reveals a steady rise in Mn^2+^ concentration in the electrolyte after charging with cycling. This does not indicate that Mn^2+^ becomes intrinsically inactive; rather suggests that the progressive accumulation of residual MnO_x_ and ZSH-related insulating byproducts on the surface reduces the number of accessible nucleation sites for redeposition. In parallel, the shrinking MnO_2_ framework continues to supply Mn^2+^ into solution over successive cycles, while the elevated Mn^2+^ concentration imposes a common-ion effect that suppresses further dissolution. Together, these findings establish that capacity fading originates from the coupled effects of incomplete redeposition and solution-phase saturation, which progressively impair the dissolution–deposition pathway.

### Mechanistic mapping of MnO_2_ charge storage: coupled dissolution–deposition and proton redox

**Fig. 5:**
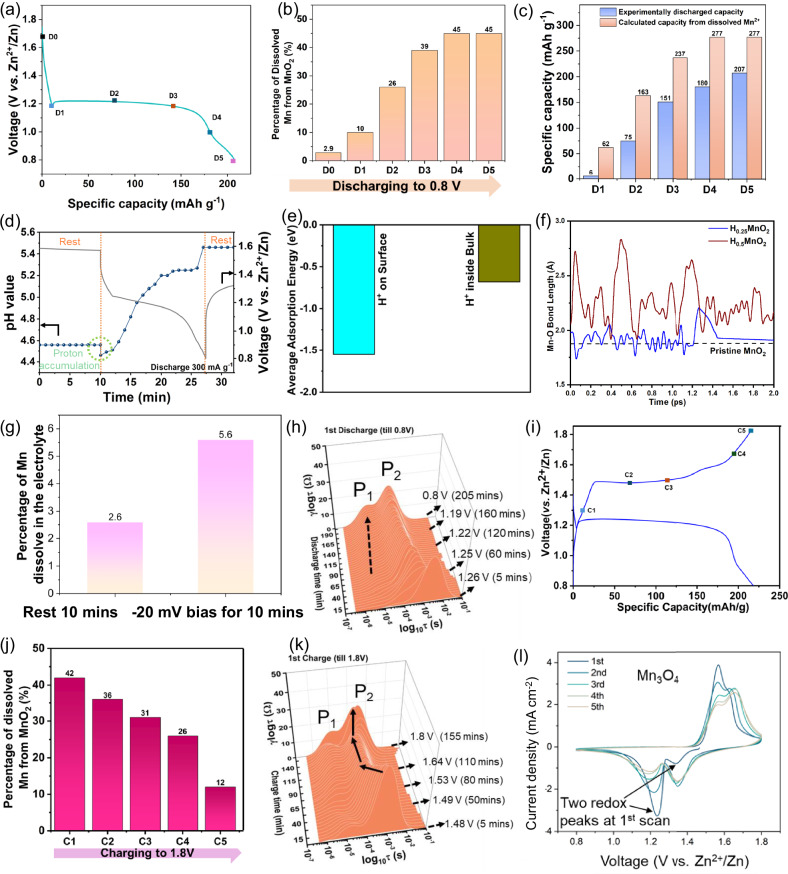
Voltage-resolved mapping of dissolution and proton redox. **a** Voltage profile (100 mA g^−1^) of 1^st^ discharge with marked sampling points (D0–D5) at which the cell was disassembled and the separator collected for ICP-OES analysis. **b** Quantification of dissolved Mn^2+^ in the electrolyte (normalized to active material mass) at each discharge stage and **c** corresponding theoretical vs. actual discharge capacities. **d** In-situ pH monitoring during the first discharge (300 mA g^−1^). **e** DFT calculation of average H⁺ adsorption energy on the α-MnO_2_ surface or bulk. **f** The evolution of surface Mn–O bond lengths from MD simulations on α-MnO_2_ at two different proton coverages (H_0.25_MnO_2_ and H_0.5_MnO_2_). **g** The ICP-OES analysis for the cell after resting or bias-holding for 10 min. **h** DRT spectra at various discharge states (0.8–1.8 V, 50 mA g^−1^). **i** Voltage profile (100 mA g^−1^) of 1^st^ charge with marked sampling points (C1–C5) at which the cell was disassembled and the separator collected for ICP-OES analysis. **j** dissolved Mn^2+^ quantification by ICP-OES, indicating redeposition. **k** DRT spectra at selected charge states (0.8–1.8 V, 50 mA g^−1^). **l** The CV scans of the as-prepared Zn||Mn_3_O_4_ cell at a scan rate of 0.2 mV s^−1^.

Based on the preceding analysis, MnO_2_ exhibits a complex interplay of dissolution–deposition and proton-coupled redox. These processes do not occur in isolation; rather, they overlap significantly throughout cycling, making it difficult to assign distinct voltage windows to each. To probe their relative contributions, we sampled the electrolyte at different discharge stages (Fig. 5a) and quantified dissolved Mn using ICP-OES. Interestingly, a measurable amount of Mn^2+^ appears dissolved in the electrolyte at the initial voltage dip (D1, Fig. 5b), accounting for nearly 10% of the Mn in the pristine electrode (Fig. 5c), even though the delivered capacity at this point (6 mAh g^−1^) is negligible. In contrast, before cycling (D0, after 1 h resting), only about 2.9% of MnO_2_ dissolves, indicating that spontaneous chemical dissolution contributes only a minor fraction relative to the early-discharge dissolution (D1). This observation indicates that there is non-faradaic MnO_2_ dissolution well before substantial discharge capacity is realized. In-situ pH monitoring corroborates this interpretation: at the onset of discharge, the potential drops rapidly to the plateau, while the pH shows a pronounced dip followed by recovery (Fig. 5d), reflecting proton accumulation at the interface and its subsequent consumption during chemical MnO_2_ dissolution. Density functional theory (DFT) calculations on the α-MnO_2_ (110) reveal that protons preferentially adsorb at surface sites rather than intercalate into the bulk lattice at the onset of discharge (Figs. S37, 5e). A pseudo-binary grand-potential convex-hull for H_x_MnO_2_ further shows that the earliest uptake (x < 0.02) occurs at high potential (Fig. S38), consistent with strong initial surface binding. As x increases (0.02 < x < 0.25), the average insertion voltage falls toward ~1.0 V (vs Zn^2+^/Zn) and then rises again toward ~1.5 V for x > 0.25, consistent with local proton accumulation and proton-primed weakening of Mn–O coordination^49,50^. Molecular dynamics (MD) simulation further confirms that increasing proton coverage lengthens and destabilizes Mn–O bonds (Fig. 5f). While in H_0.25_MnO_2_ the bonds fluctuate near the pristine value, in H_0.5_MnO_2_ they remain persistently elongated with larger excursions (often > 2.4–2.6 Å). Thus, surface protonation acts as a preconditioning step that facilitates Mn–O bond scission and triggers Mn dissolution. A plausible explanation is that under the proton primed condition, Mn^3+^ domains in the surface and sub-surface layers undergo disproportionation or short-range internal electron exchange with Mn^4+^, to lead to Mn^2+^ dissolution before any measurable faradaic current is passed; although our data support this pathway, we cannot entirely rule out a minor contribution from water oxidation under these highly protonated conditions, providing electrons for MnO_2_ reduction. The resulting defective and under-coordinated surface further facilitates proton adsorption and even near-surface insertion, rationalizing the calculated step-up in insertion voltage for x > 0.25. Notably, this later near-surface insertion is distinct from the initial protonation event that drives non-faradaic dissolution. Consistent with a proton-triggered pathway, applying a small cathodic bias (–25 mV vs open circuit potential, 10 min)—chosen to avoid faradaic currents—produces a measurably high Mn^2+^ concentration in the electrolyte (Fig. 5g). SEM and XRD already reveal ZSH precipitation at the D1 stage (Figs. S39, S40), confirming the expected pH-coupled byproduct formation.

As discharge proceeds through the main plateau (D1–D3), extensive MnO_2_ dissolution is observed alongside a commensurate rise in capacity (Fig. 5b, c). During this interval, XRD and electron microscopy show uniform ZSH coverage across the electrode (Figs. S39, S40). Notably, in the D2–D4 region, the measured capacity lags the capacity implied by the dissolved Mn by ~70–80 mAh g^−1^, primarily due to earlier non-faradaic dissolution at D1. Entering the low-voltage region (D4–D5, 1.0–0.8 V), the cell still delivers ~27 mAh g^−1^ without an increase in dissolved Mn^2+^, signaling a mechanistic transition wherein proton intercalation dominates over further MnO_2_ dissolution.

To further disentangle the concurrent processes, DRT (distribution of relaxation times) analysis was performed on the electrochemical impedance spectroscopy (EIS) data, collected in situ during discharge (Fig. S41). Two distinct features become apparent (Fig. 5h) in the DRT profiles: a mid-frequency peak at ~10^−3^ s (denoted P_2_) and a high-frequency peak at ~10^−5^ s (denoted P_1_). To more rigorously assign these peaks, we tuned Mn^2+^ activity and electrode porosity (affects interfacial accessibility) by adding 0.2 M MnSO_4_ in the electrolyte and by calendering the electrode, respectively. Increasing Mn^2+^ selectively suppressed P2, whereas calendering enhanced both peaks. These trends indicate that P1 originates from a proton-driven surface process, while P2 reflects dissolution–deposition charge-transfer kinetics (see Fig. S42 for detailed discussion). Notably, P2 is pronounced at the onset of discharge, consistent with a sizable kinetic barrier for initial Mn–O bond scission. Its subsequent attenuation and stabilization indicate that electrochemical MnO_2_ dissolution becomes progressively more facile as the surface evolves under operation. The emergence of a high-frequency (P_1_) process toward the end of discharge is also consistent with the transition from dissolution-dominated to proton intercalation-dominated behavior, as supported by ICP-OES and EELS analysis. The increase in insulating ZSH coverage on the positive electrode surface with increasing depth of discharge is expected to increase the kinetic barrier to proton intercalation, which explains the rise in the P_1_ intensity.

The progressive decrease in Mn^2+^ concentration in the electrolyte throughout charging (Fig. 5i, j) reflects the continuous electrodeposition of Mn^2+^, coupled with proton generation, that drives ZSH dissolution (Fig. S43). Owing to ZSH’s buffering effect, the bulk electrolyte pH remains near 5.8 during charging (Fig. S44) and recovers to ~5.4 upon resting. Given that the interfacial pH is likely higher than the measured value, MnO_x_ formation may proceed via a Mn(OH)_2_ intermediate (Fig. S45). In the EIS-DRT analysis (Fig. 5k, Fig. S46), the P_2_ peak exhibits high intensity at the early stage, reflecting sluggish Mn^2+^ oxidation-deposition hindered by ZSH-related passivation at the surface. As ZSH dissolves, P_2_ declines and stabilizes, indicating improved accessibility of active sites. Near full charge, however, P_2_ intensifies again, likely due to newly deposited MnO_x_ masking surface sites and impeding further deposition. P_1_ peak, corresponding to the de-intercalation of a proton, rises at the end of charge, indicating the completion of the ion-extraction process. This parallel evolution suggests that both Mn^2+^ deposition and proton de-intercalation are governed by dynamic changes in surface accessibility during charging.

Given that the deposited MnO_x_ phase displays an Mn valence state (mixture of Mn^2+^/Mn^3+^, + 2.56) closely resembling Mn_3_O_4_, this phase was chemically synthesized (Fig. S47) and evaluated as a positive electrode to deconvolute the voltage profile. Consistent with prior reports^51,52^, Mn_3_O_4_ displays two distinct redox peaks during the first cathodic scan in CV (Fig. 5l). The same is reflected in the GCD profile **(**Fig. S48). A similar dual-peak signature emerges for α-MnO_2_, but from the second cycle onward. This shift reflects a mechanistic transition: the initial discharge primarily involves dissolution of Mn^4+^ from the parent α-MnO_2_, producing a single plateau around 1.13 V (Fig. 1c). The two plateaus in the later cycles correspond to the dissolution of newly formed MnO_x_ (~1.38 V) and the continued dissolution of the parent MnO_2_ (~1.13 V), accompanied by proton intercalation, particularly within the residual MnO_x_ phase. This pathway is not exclusive to α-MnO_2_; similar trends in β- and δ-MnO_2_ (Figs. S49–S51) confirm its universality in mildly acidic electrolytes, where proton storage plays a noteworthy role. Notably, although the deposited product exhibits an average Mn oxidation state and electrochemical profile similar to Mn_3_O_4_, and a prior study has reported the formation of spinel Mn_3_O_4_ after long-term cycling^53^, its nanocrystalline disordered character, as observed in this study, precludes an unambiguous assignment. We therefore refer to the deposited phase as simply MnO_x_ or mixed-valent MnO_x_ throughout and use Mn_3_O_4_ only as a valence benchmark for comparison.

Overall, the in-depth multi-modal mechanistic analyses establish that the MnO_2_ charge storage involves a proton-triggered dissolution–deposition pathway intertwined with proton (de)intercalation-mediated redox (Fig. 1). The discharge process initiates with proton adsorption that primes Mn–O bond scission, followed by MnO_2_ dissolution and minor surface-limited proton intercalation in parent unreacted MnO_2_. Charging reverses this sequence through deposition of nanocrystalline disordered MnO_x_, characterized by a mixed valence state (Mn^3+^/Mn^2+^), and proton de-intercalation from H_x_MnO_2_. ZSH, formed as a consequence of interfacial pH shifts during dissolution and proton intercalation, contributes to the passivating layer that limits redox accessibility and capacity utilization. Over cycling, MnO_x_ becomes the primary redox-active phase, while the parent MnO_2_ continues to slowly transform to MnO_x_, which accumulates owing to irreversibility in electrochemical dissolution-deposition, and serves as an active host for proton (de)intercalation, contributing nearly a third of the observed discharge capacity. The mechanistic steps are summarized by the governing reactions in Supplementary Note 1.

### Facilitating electrochemical reactions through surface and architectural design

**Fig. 6:**
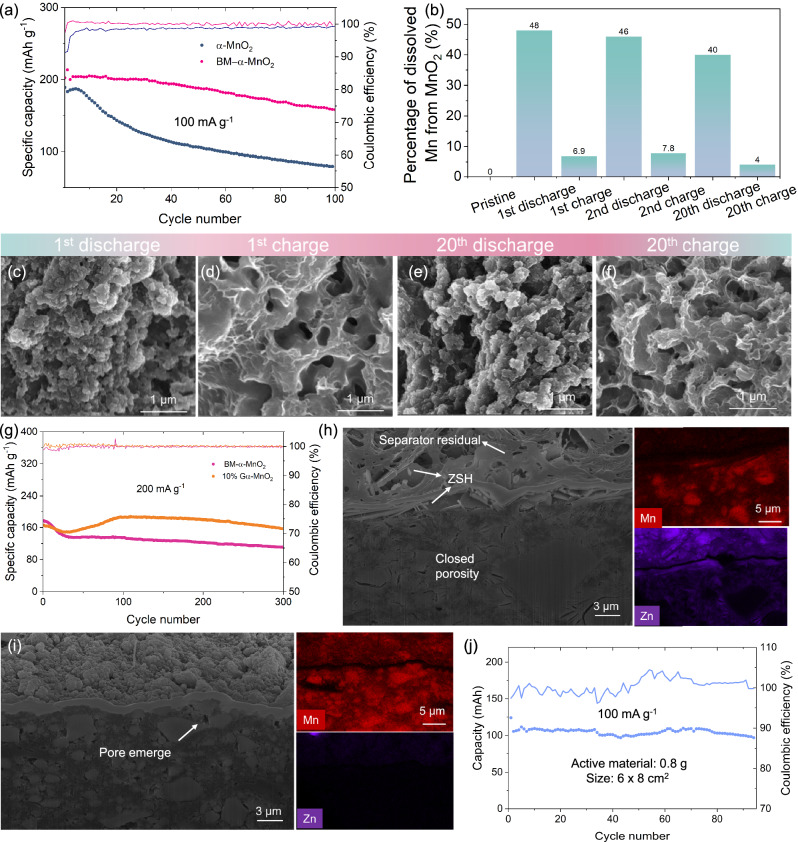
Surface and architecture design enable improved long-term reversibility. **a** The comparison of long-term cyclability between BM-α-MnO_2_ and pristine α-MnO_2_ in 1 M ZnSO_4_ electrolyte at 100 mA g^−1^. **b** ICP-OES results evaluating the extent and reversibility of the dissolution-deposition chemistry in BM-α-MnO_2._ The SEM images to elucidate the morphology evolution of BM-α-MnO_2_ between **c**, **d** 1^st^ cycle and **e**, **f** 20^th^ cycle. **g** Long-term performance comparison between 10% Gra-α-BM-MnO_2_ and BM-α-MnO_2_ electrode at 200 mA g^−1^. The FIB-SEM image for 10% Gra-BM-α-MnO_2_ after the first **h** discharge and **i** charge, accompanied by corresponding EDS mappings for Zn and Mn. **j** The 100-mAh 10% Gra-BM-α-MnO_2_||Zn pouch cell cycled under 100 mA g^−1^ to confirm the efficacy of graphite additive.

Given the surface-dependent nature of both proton (de)intercalation and dissolution-deposition processes, α-MnO_2_ was subjected to high-energy ball milling (denoted as BM-α-MnO_2_) to break down the nanorod morphology and construct a composite with integrated conductive networks (Fig. S52). This treatment introduced more accessible active sites and defective surface structures, thereby facilitating both Mn^2+^ deposition/dissolution and proton-coupled redox reactions. It is worth mentioning that, typically, MnO_2_ electrodes are tested at low active mass loadings (~1 mg cm^−2^) to mitigate polarization, which stems not only from MnO_2_’s poor electronic conductivity but also from sluggish ionic transport and interfacial charge-transfer limitations, particularly under high-rate conditions. When combined with electrolytes containing Mn^2+^ additives (commonly ~0.2 M), this practice can artificially inflate the apparent electrode capacity (Fig. S53). To avoid such effects, the electrochemistry of BM-α-MnO_2_ was first evaluated with a moderate active loading of ~3-4 mg cm^−2^ at 100 mA g^−1^ with 1 M ZnSO_4_ electrolyte without any Mn^2+^ additive. As indicated by Fig. S54, the BM-α-MnO_2_ retains the characteristic voltage profile of pristine α-MnO_2_ but delivers a slightly higher first-cycle capacity of 213 mAh g^−1^, attributed to enhanced proton intercalation. More importantly, BM-α-MnO_2_ exhibits markedly improved cycling stability (Fig. 6a), enabled by its higher surface area and enhanced electrochemical activity. ICP-OES analysis (Fig. 6b) confirms that ~48% of active Mn participates in dissolution during the first discharge, with substantial Mn^2+^ redeposition during charging—even after prolonged cycling—highlighting the effectiveness of the surface design. Morphological evolution of the electrode further corroborates the pronounced reversibility (Fig. 6c–f). Unlike the pristine α-MnO_2_, the discharged BM-α-MnO_2_ retains its characteristic granular morphology even after 20 cycles without being obscured by residual MnO_x_ deposits (Fig. 6e**)**. Upon subsequent charging (Fig. 6f), the redeposited MnO_x_ nanosheets uniformly cover the positive electrode surface, reflecting highly efficient and reversible dissolution-deposition behavior.

While enhancing the surface-active sites is critical to improving the electrochemical reversibility of MnO_2_, the dynamic formation of a secondary phase (ZSH) during cycling necessitates a well-optimized electrode architecture. In particular, engineered porosity is critical for accommodating ZSH and maintaining reaction kinetics over long-term operation. To this end, 10 wt% battery-grade graphite (particle size: 1–3 μm) was incorporated into the BM-α-MnO_2_ electrode (denoted 10% Gra-BM-α-MnO_2_) as a partial replacement for conductive carbon as a porosity regulator. Focused ion beam (FIB)–SEM imaging (Fig. S55) reveals well-defined voids around graphite particles, providing internal reservoirs for ZSH precipitation. Leveraging this tailored porous architecture, the 10% Gra-BM-α-MnO_2_ electrode achieves markedly improved cycling stability, retaining 157 mAh g^−1^ after 300 cycles at 200 mA g^−1^ (94.5% retention from an initial 166 mAh g^−1^, Fig. 6g). Post-discharge analysis shows extensive ZSH deposits across the surface (Fig. 6h) and within internal pores, as confirmed by pronounced EDS signals of Zn within the electrode interior coupled with the observable filling of internal pores. Upon charging, these deposits dissolve completely, restoring the pore network (Fig. 6i). In contrast to the micron-sized hexagonal ZSH byproducts, the MnO_x_ deposited during charging exhibits a nanosheet morphology, which allows the internal pore structure to remain largely preserved. This tailored architecture thus mitigates ZSH-induced kinetic barriers, enabling efficient phase accommodation and delivering improved long-term cycling stability compared to BM-α-MnO_2_ alone. As a proof of concept, the Gra-BM-α-MnO_2_ positive electrode was assembled into a 6 × 8 cm^2^ pouch cell and cycled at 100 mA g^−1^. Consistent with the improved performance at the small scale, the pouch cell delivers 100 mAh of capacity and maintains it for nearly 100 cycles without significant capacity fading (Fig. 6j) before failing owing to the zinc depletion-mediated polarization.

Taken together, this report resolves the long-standing mechanistic ambiguity surrounding MnO_2_ electrochemistry in aqueous zinc-ion batteries by establishing a unified mechanism governed by coupled dissolution–deposition and reversible proton-intercalation redox. Our results unequivocally rule out Zn^2+^ insertion or associated conversion pathways, as well as bulk proton intercalation in the parent MnO_2_ as dominant contributors. Discharge initiates via proton-primed Mn–O bond destablization and chemical Mn^2+^ dissolution; thereafter, faradaic dissolution governs the first discharge. During charge, Mn^2+^ redeposits as disordered nanocrystalline mixed-valent (Mn^2+/3+^) MnO_x_, which subsequently becomes the dominant redox-active phase.

As cycling progresses, capacity is largely dominated by two processes: dissolution–deposition of MnO_x_ and proton intercalation within the residual MnO_x_, with the latter contributing nearly one-third of the obtained capacity, while dissolution–deposition and shallow surface proton redox of the remaining parent MnO_2_ have only limited contributions. Interfacial pH–driven ZSH precipitation and its associated byproducts form part of the passivating layer, which restricts surface accessibility, limits dissolution depth, and ultimately constrains capacity utilization. Taken together, these evolutions highlight that long-term reversibility is governed by interfacial accessibility rather than intrinsic redox limitations, underscoring the need for thoughtful structural and interfacial engineering. By integrating surface activation and tailored porosity, we demonstrate strategies that mitigate these bottlenecks and deliver stable performance at high active loadings. These findings bridge fundamental understanding with design principles, offering a blueprint for further optimization, potentially involving carbon networks and binder chemistry, to enable high-loading electrode rolls and pouch cells without sacrificing durability. Such advances will bridge the gap between laboratory-scale performance and practical deployment, positioning Mn-oxide positive electrodes as a cornerstone for safe, affordable, and sustainable grid-scale energy storage.

## Methods

### Materials

Potassium permanganate (KMnO_4_, ≥99%), zinc sulfate heptahydrate (ZnSO_4_·7H_2_O, ≥99%), manganese(II) sulfate monohydrate (MnSO_4_·H_2_O, ≥99%), Mn(NO_3_)_2_·4H_2_O ( ≥ 99.9%), Mn(Ac)_2_·4H_2_O, manganese(II) oxide (MnO, ≥99%), manganese dioxide (MnO_2_, ≥99%), N_2_H_4_·H_2_O ( ≥ 97%), ethylenediaminetetraacetic acid disodium salt dihydrate (EDTA-2Na, ≥99%) were all purchased from Sigma-Aldrich and used directly without further treatment. Sulfuric acid (H_2_SO_4_, 98%) and nitric acid (HNO_3_, 65%) were purchased from RCI LABSCAN. The conductive carbon (Super P) and battery-grade graphite were obtained from MSE Supplies LLC, AZ, USA. These chemicals are all used as received without further purification.

### Synthesis of α-MnO_2_ nanorods

To synthesize α-MnO_2_ nanorods via the hydrothermal method, 12 mmol of KMnO_4_ was dissolved in 24 mL of water, followed by the addition of 1 mL of concentrated H_2_SO_4_. The resulting solution was then transferred into a 46 mL Teflon^TM^ vessel, which was placed inside a stainless-steel autoclave, heated at 150 °C for 12 h, and then cooled naturally to room temperature. The synthesized α-MnO_2_ nanorods were collected by filtration, washed with deionized water, and dried in air at 80 °C, yielding a brown powder.

### Synthesis of β-MnO_2_ nanorods

The β-MnO_2_ was prepared by directly calcining Mn(NO_3_)_2_·H_2_O at 350 °C for 5 h.

### Synthesis of Mn(OH)_2_

In a typical synthesis, 4 mmol of Mn(Ac)_2_·4H₂O was dissolved in 40 mL of a mixed solvent of ethylene glycol (EG) and water under vigorous stirring for 5 h to achieve a homogeneous solution. Subsequently, 5 mL of N_2_H_4_·H_2_O was added at room temperature, and the mixture was stirred for an additional 4 min. The resulting solution was then transferred into a 125 mL Teflon-lined autoclave, sealed, and heated at 180 °C for 12 h. After natural cooling to room temperature, the precipitate was collected, washed three times with absolute ethanol, vacuum-dried for 3 h, and stored for further characterization.

### Synthesis of Mn_2_O_3_

To obtain Mn_2_O_3_ powder, the commercial MnO_2_ powder (Sigma-Aldrich) was thermally treated at 700 °C for 5 h in a muffle furnace with a controlled heating rate of 5 °C min^−1^.

### Synthesis of Mn_3_O_4_

2 mL of 0.5 M EDTA-2Na solution and 5 mL of 0.2 M KMnO_4_ aqueous solution were combined in a 50 mL Teflon vessel. DI water was then added to the vessel until 70% of the total volume was reached. The solution’s pH was adjusted to approximately 6.0 using 2 M HNO_3_ and vigorously stirred with a magnetic stirrer. The Teflon-lined autoclave was then securely sealed and heated at 180 °C for 4 h, followed by natural cooling to room temperature. The resulting precipitates were collected by filtration, thoroughly washed with distilled water and ethanol, and finally dried at 60 °C overnight.

### Synthesis of BM-α-MnO_2_

The hydrothermally synthesized α-MnO₂ nanorods were filled into a ball milling jar and milled with zirconia balls (Ø = 3 mm) using a Pulverisette 7 (Fritsch) at 500 rpm for 3 h to break the nanorods. Afterward, the resulting powder was collected from the bowl and was ready to make the electrode.

### Characterizations

For ex situ characterization, cycled electrodes were rinsed sequentially with deionized water and ethanol to remove residual electrolyte. Where removal of ZSH surface deposits was required, electrodes were immersed in 0.1 M H_2_SO_4_, followed by a second rinse with deionized water and ethanol. All ex situ XRD data were collected using PANalytical Xpert Multipurpose X-ray Diffraction System (MPD) with Cu Kα radiation (λ = 1.54178 Å). Operando XRD was conducted on a PANalytical Empyrean II diffractometer in Bragg−Brentano geometry using Co K_α_ radiation (λ = 1.788965 Å) and a Pixel detector with a Ni K_β_ filter. The diffraction data were collected in the reflection mode from 5° to 50° (2θ) with a collection time of 15 min. The working electrodes were prepared using glassy carbon as the current collector, and the active mass loading was around 10 mg cm^−2^. For operando synchrotron X-ray diffraction, homemade coin cells with Kapton tape windows were assembled and measured at the Powder Diffraction Beamline of the Australian Synchrotron (λ = 0.5904 Å).

The field-emission scanning electron microscopy (FESEM) images were taken on an FEI Nova NanoSEM 450 with an operating voltage of 5 kV, equipped with an EDS attachment. The TEM images were taken on JEOL2100 F with an acceleration voltage of 200 kV, equipped with an energy dispersive spectrometer.

For ICP-OES analysis, the cycled separator was disassembled from the cell and soaked in 5 mL DI H_2_O for half an hour. This diluted solution was then subjected to ICP-OES testing using an Optima 8000 instrument.

Soft X-ray Absorption Spectroscopy (NEXAFS) was performed at the soft X-ray beamline of the Australian Synchrotron (ANSTO). These measurements focused on the Mn L-edge. Spectra were acquired in Auger electron yield (AEY), partial electron yield (PEY), total electron yield (TEY), and fluorescence yield (FY) modes, with an energy step size of 0.1 eV. All NEXAFS spectra were normalized and processed using the QANT software made available by ANSTO. The LCF (linear combination fitting) was performed over the Mn L_3_ and L_2_ edges using reference spectra for Mn(II), Mn(III), mixed-valent Mn, and Mn(IV), collected and processed under identical conditions. The average Mn oxidation state was obtained as the coefficient-weighted sum of the nominal oxidation states.”

### Electrochemical measurements

Swagelok cells (Perfluoroalkoxy alkane or PFA-based body with 12.7 mm internal diameter) with Ti current collectors were used for all the galvanostatic cycling. The working electrode was fabricated by blending active materials (including α, β and δ-MnO_2_ and Mn_3_O_4_), conductive carbon, and polyvinylidene fluoride (PVDF) in mortar with pestle in a weight ratio of 70:25:5 using N-methyl-2-pyrrolidone (NMP, ≥99.7%, Sigma-Aldrich) as the solvent. Similarly, 10%GBM-α-MnO_2_ was prepared by mixing BM-α-MnO_2_ with graphite powder, conductive carbon and PVDF binders in a weight ratio of 70:10:15:5 using NMP as the solvent. The slurry was doctor-bladed onto clean graphite foil under ambient conditions, and vacuum-dried at 80 ˚°C for 12 h. The cast foil was die-cut into 11 mm diameter disks with an active positive electrode mass loading of 4–6 mg cm^−2^. A glass fiber filter paper (420 μm thick, 12.7 mm diameter, Whatman, GF/F) and zinc foil (50 μm thick, 11 mm diameter, 99.9%, Gelonlib) were employed as the separator and negative electrode, respectively, while aqueous 1 M ZnSO_4_ solution (50 µL) without MnSO_4_ additive was used as the electrolyte. For pouch cell preparation, the positive electrode slurry was cast on the double side of carbon-coated stainless steel with a mass loading of ~7–8 mg cm^−2^ for a single layer. The cast stainless steel foil was cut into 48 cm^2^ sheets and soldered with nickel tabs for external connection. The electrode stack was sealed in a laminated aluminum pouch bag under vacuum after injecting 1 M ZnSO_4_ electrolyte (35 μL cm^−2^). An external pressure of ~200 kPa was applied during cycling. All electrochemical measurements were conducted at a controlled room temperature of 20 ± 2 °C. The assembled Swagelok cells were galvanostatically charged/discharged in a 0.8–1.8 V window using a LAND CT2001A cycler. All cycling performance assessments were conducted across at least three independent cells. Cyclic voltammetry was measured in a two-electrode setup in a 0.8–1.8 V window at a scan rate of 0.2 mV s^−1^ (VMP-3, BioLogic). To probe the dissolution behavior, Swagelok cells were disassembled after discharge, and the cycled separator was replaced with a fresh one along with 1 M ZnSO_4_ electrolyte before subjecting the cell to the subsequent charging process. Potentiostatic EIS data were collected in the 1 MHz – 0.1 Hz frequency range by applying a 10 mV signal amplitude, acquiring 10 points per decade. For DRT analysis, the EIS is obtained after reaching equilibrium state (resting for ~3–4 h) after every 5 min charge/ discharge. The obtained EIS data were further analyzed by Python-based pyDRTtools software (developed by Ciucci’s lab) to deconvolute the charge transfer process. For mathematical deconvolution, the second-order Gaussian radial basis functions (RBF) was employed with shape factor control between 0.75 to 1.0 and a regularization parameter of 0.001^54–56^. In-situ pH measurements were performed using a three-electrode Swagelok-type cell configuration specifically designed to minimize electrolyte usage. The working and counter electrodes were affixed to two parallel titanium rods, while a micro-pH electrode was inserted from the top to monitor interfacial pH changes. The distance between the WE and CE electrodes was maintained at 5 mm, and the electrolyte volume was restricted to 500 μL.

### Computational methods

Electronic structure calculations were performed using Density Functional Theory (DFT), as implemented in the plane-wave VASP^57,58^ code. Core-valence electron interactions were treated using the Projector Augmented Wave^59^(PAW) method, and the Perdew-Burke-Ernzerhof^[ 60^(PBE) exchange-correlation functional was employed to solve the Kohn-Sham equations^61^. A Г-centered k-point mesh of 1 x 1 x 1 was used for the surface calculations, and the plane-wave was expanded with a kinetic energy cutoff of 500 eV. The electronic relaxation criterion was set to 10^−5^ eV for all the calculations. The bulk MnO_2_ structure was obtained from the Materials Project^62^ database and fully relaxed using appropriate k-point sampling. The MnO_2_ (110) surface was generated by cleaving the optimized bulk structure using the Atomistic Simulation Environment^63^ (ASE) package. To address the excessive delocalization of the 3 d electrons in Mn, the Dudarev^64^ et al. formalism with an effective U value of 3.9 eV was employed. To simulate both surface- and bulk-like regions in the slab, the central layer was fixed at its optimized positions, whereas the top and bottom layers were allowed to relax.

Following geometric relaxation of the pristine MnO_2_ (110) surface (see Supplementary Data 1, 2), the optimized structure was used to probe H^+^ adsorption on the surface and within the bulk (H_0.02_MnO_2_, see Supplementary Data 3–6)

The adsorption energy of the proton on the substrate was calculated using the equation:

1 $${E}_{{ads}}^{{Proton}+{Surface}}={E}_{{Total}}^{{Proton}+{Surface}}-({E}_{{Total}}^{{Proton}}+{E}_{{Total}}^{{Surface}})$$

Where ${E}_{{ads}}^{{Proton}+{Surface}}$ is the adsorption energy of the cluster on the surface, ${E}_{{Total}}^{{Proton}+{Surface}}$ is the total energy of the combined molecule/surface system, ${E}_{{Total}}^{{Proton}}$ and ${E}_{{Total}}^{{Surface}}$ are the total energies of the individual molecules and the surface slab, relaxed to their optimized geometries, respectively.

The intercalation voltage profile is quantitatively calculated by the following relation and then transposed to that with respect to the Zn/Zn^2+^ voltage:

2 $$V=-\frac{E\left({H}_{{x}_{2}}{Mn}{O}_{2}\right)-E\left({H}_{{x}_{1}}{Mn}{O}_{2}\right)-\left({x}_{2}-{x}_{1}\right){E}_{A}\,}{\left({x}_{2}-{x}_{1}\right)e}$$

where *V* represents the average voltage, *x*_*1*_ and *x*_*2*_ (*x*_*2*_ > *x*_*1*_) are the number of H^+^ ions intercalated/de-intercalated, while *E* and *E*_*A*_ denote the total energy and energy per atom in the corresponding ionic phase, respectively. The H_0.25_MnO_2_ and H_0.5_MnO_2_ configurations (see Supplementary Data 7–10) were fully relaxed and used to construct the voltage profile, alongside surface H^+^ adsorption states (see Supplementary Data 3, 4)

Ab initio MD (AIMD) simulations of proton-intercalated structures were performed in the NVT ensemble at 300 K. The temperature was regulated using a Nosé-Hoover chain thermostat with a time step of 2 fs. AIMD simulations were carried out to evaluate the stability of the H_0.25_MnO_2_ and H_0.5_MnO_2_ configurations (see Supplementary Data 11–14).

## Supplementary information

Supplementary Information

Description of Additional Supplementary Files

Supplementary Data 1–14

Transparent Peer Review file

## Source data

Source Data

## Data Availability

The data supporting the findings of this study are included within the ‘Source Data’ file, accessible with this paper Source data are provided with this paper.

## References

[1] Li, C., Jin, S., Archer, L. A. & Nazar, L. F. Toward practical aqueous zinc-ion batteries for electrochemical energy storage. *Joule* **6**, 1733–1738 (2022).

[2] Shang, Y. & Kundu, D. A path forward for the translational development of aqueous zinc-ion batteries. *Joule* **7**, 244–250 (2023).

[3] Chao, D. et al. Roadmap for advanced aqueous batteries: From design of materials to applications. *Sci. Adv.* **6**, eaba4098 (2020). 32494749 10.1126/sciadv.aba4098 PMC7244306

[4] Wang, F. et al. Highly reversible zinc metal anode for aqueous batteries. *Nat. Mater.* **17**, 543–549 (2018). 29662160 10.1038/s41563-018-0063-z

[5] Ma, L. et al. Highly reversible Zn metal anode enabled by sustainable hydroxyl chemistry. *Proc. Nat. Acad. Sci.* **119**, e2121138119 (2022). 35675422 10.1073/pnas.2121138119 PMC9214537

[6] Li, C. et al. Highly reversible Zn anode with a practical areal capacity enabled by a sustainable electrolyte and superacid interfacial chemistry. *Joule* **6**, 1103–1120 (2022).

[7] Cao, L. et al. Fluorinated interphase enables reversible aqueous zinc battery chemistries. *Nat. Nanotech.* **16**, 902–910 (2021). 10.1038/s41565-021-00905-4 33972758

[8] Chen, R. et al. Rational design of an in-situ polymer-inorganic hybrid solid electrolyte interphase for realising stable Zn metal anode under harsh conditions. *Angew. Chem. Int. Ed.,* **136**, e202401987 (2024). 10.1002/anie.202401987 PMC11497294 38526053

[9] Li, C. et al. Enabling selective zinc-ion intercalation by a eutectic electrolyte for practical anodeless zinc batteries. *Nat. Commun.* **14**, 3067 (2023). 37244907 10.1038/s41467-023-38460-2 PMC10224959

[10] Wang, S. et al. A parts-per-million scale electrolyte additive for durable aqueous zinc batteries. *Nat. Commun.* **16**, 1800 (2025). 39979314 10.1038/s41467-025-56607-1 PMC11842810

[11] Zheng, J. & Archer, L. A. Controlling electrochemical growth of metallic zinc electrodes: Toward affordable rechargeable energy storage systems. *Sci. Adv.* **7**, eabe0219 (2021). 33523975 10.1126/sciadv.abe0219 PMC7787491

[12] Li, C. et al. Scalable high-voltage Zn||MnO2 batteries achieved by mild amphiphilic hydrogel electrolytes. *Proc. Nat. Acad. Sci.* **122**, e2501935122 (2025). 40815628 10.1073/pnas.2501935122 PMC12377733

[13] Liu, S. et al. Zinc ion batteries: bridging the gap from academia to industry for grid-scale energy storage. *Angew. Chem. Int. Ed.,* **63**, e202400045 (2024). 10.1002/anie.202400045 38385624

[14] Liu, J., Shen, Z. & Lu, C.-Z. Research progress of Prussian blue and its analogues for cathodes of aqueous zinc ion batteries. *J. Mater. Chem. A* **12**, 2647–2672 (2024).

[15] Xu, C., Li, B., Du, H. & Kang, F. Energetic zinc ion chemistry: the rechargeable zinc ion battery. *Angew. Chem. Int. Ed.,* **51**, 933–935 (2012). 10.1002/anie.201106307 22170816

[16] Zhang, N. et al. Rechargeable aqueous zinc-manganese dioxide batteries with high energy and power densities. *Nat. Commun.* **8**, 405 (2017). 28864823 10.1038/s41467-017-00467-x PMC5581336

[17] Lee, B. et al. Electrochemically-induced reversible transition from the tunneled to layered polymorphs of manganese dioxide. *Sci. Rep.* **4**, 6066 (2014). 25317571 10.1038/srep06066 PMC5377529

[18] Pan, H. et al. Reversible aqueous zinc/manganese oxide energy storage from conversion reactions. *Nat. Energy* **1**, 16039 (2016).

[19] Li, Y. et al. Reaction mechanisms for long-life rechargeable Zn/MnO2 batteries. *Chem. Mater.* **31**, 2036–2047 (2019).

[20] Sun, W. et al. Zn/MnO2 battery chemistry with H+ and Zn2+ coinsertion. *J. Am. Chem. Soc.* **139**, 9775–9778 (2017). 28704997 10.1021/jacs.7b04471

[21] Gao, X. et al. H+-insertion boosted α-MnO2 for an aqueous Zn-ion battery. *Small* **16**, 1905842 (2020). 10.1002/smll.201905842 31916666

[22] Oberholzer, P., Tervoort, E., Bouzid, A., Pasquarello, A. & Kundu, D. Oxide versus nonoxide cathode materials for aqueous Zn batteries: an insight into the charge storage mechanism and consequences thereof. *ACS Appl. Mater. Interfaces* **11**, 674–682 (2019). 30521309 10.1021/acsami.8b16284

[23] Yuan, Y. et al. Understanding intercalation chemistry for sustainable aqueous zinc–manganese dioxide batteries. *Nat. Sustain.* **5**, 890–898 (2022).

[24] Moon, H. et al. Direct proof of the reversible dissolution/deposition of Mn2+/Mn4+ for mild-acid Zn-MnO2 batteries with porous carbon interlayers. *Adv. Sci.* **8**, 2003714 (2021). 10.1002/advs.202003714 PMC7967064 33747744

[25] Wu, D. et al. Simultaneous elucidation of solid and solution manganese environments via multiphase operando extended X-ray absorption fine structure spectroscopy in aqueous Zn/MnO2 batteries. *J. Am. Chem. Soc.* **144**, 23405–23420 (2022). 36513373 10.1021/jacs.2c09477 PMC9801424

[26] Ye, X. et al. Unraveling deposition/dissolution chemistry of MnO2 for high-energy aqueous batteries. *Energy Environ. Sci.* **16**, 1016–1023 (2023).

[27] Xiao, X. et al. Ultrahigh-loading manganese-based electrodes for aqueous batteries via polymorph tuning. *Adv. Mater.* **35**, 2211555 (2023). 10.1002/adma.202211555 37149287

[28] Liao, Y. et al. Unveiling performance evolution mechanisms of MnO2 polymorphs for durable aqueous zinc-ion batteries. *Energy Storage Mater.* **44**, 508–516 (2022).

[29] Li, Y. et al. Revealing the dominance of the dissolution-deposition mechanism in aqueous Zn−MnO2 batteries. *Angew. Chem. Int. Ed.,* **63**, e202318444 (2024). 10.1002/anie.202318444 38116912

[30] Cui, S., Zhang, D., Zhang, G. & Gan, Y. Reaction mechanism for the α-MnO2 cathode in aqueous Zn ion batteries revisited: elucidating the irreversible transformation of α-MnO2 into Zn–vernadite. *J. Mater. Chem. A* **10**, 25620–25632 (2022).

[31] Li, L. et al. Functioning mechanism of the secondary aqueous Zn-β-MnO2 battery. *ACS Appl. Mater. Interfaces* **12**, 12834–12846 (2020). 32091201 10.1021/acsami.9b22758

[32] Liu, C. et al. Unveiling capacity limitations of MnO2 in rechargeable Zn chemistry. *Energy Environ. Sci.* **18**, 9611–9622 (2025).

[33] Kankanallu, V. R. et al. Elucidating a dissolution-deposition reaction mechanism by multimodal synchrotron X-ray characterization in aqueous Zn/MnO2 batteries. *Energy Environ. Sci.* **16**, 2464–2482 (2023).

[34] Garvie, L. A. J. & Craven, A. J. High-resolution parallel electron energy-loss spectroscopy of Mn L2,3-edges in inorganic manganese compounds. *Phys. Chem. Minerals* **21**, 191–206 (1994).

[35] Tan, H., Verbeeck, J., Abakumov, A. & Van Tendeloo, G. Oxidation state and chemical shift investigation in transition metal oxides by EELS. *Ultramicroscopy* **116**, 24–33 (2012).

[36] Yang, W. et al. Comparative study of α-, β-, γ- and δ-MnO2 on toluene oxidation: oxygen vacancies and reaction intermediates. *Appl. Catal. B: Environ.* **260**, 118150 (2020).

[37] Xiong, T. et al. Defect engineering of oxygen-deficient manganese oxide to achieve a high-performing aqueous zinc ion battery. *Adv. Energy Mater.* **9**, 1803815 (2019).

[38] Wu, L. et al. Revisiting the charging mechanism of α-MnO2 in mildly acidic aqueous zinc electrolytes. *Small* **20**, 2404583 (2024). 10.1002/smll.202404583 39077979

[39] Lee, B. et al. Critical role of pH evolution of electrolyte in the reaction mechanism for rechargeable zinc batteries. *ChemSusChem* **9**, 2948–2956 (2016). 27650037 10.1002/cssc.201600702

[40] Guo, X. et al. Zn/MnO2 battery chemistry with dissolution-deposition mechanism. *Mater. Today Energy* **16**, 100396 (2020).

[41] Li, H. et al. Interface-regulated MnO2/Mn2+ redox chemistry in aqueous Zn ion batteries. *Chem. Eng. J.* **446**, 137205 (2022).

[42] Wu, D. et al. Quantitative temporally and spatially resolved X-ray fluorescence microprobe characterization of the manganese dissolution-deposition mechanism in aqueous Zn/α-MnO2 batteries. *Energy Environ. Sci.* **13**, 4322–4333 (2020).

[43] Siamionau, U. et al. Rechargeable zinc-ion batteries with manganese dioxide cathode: How critical is the choice of manganese dioxide polymorphs in aqueous solutions? *J. Power Sources* **523**, 231023 (2022).

[44] Lin, F. et al. Synchrotron X-ray analytical techniques for studying materials electrochemistry in rechargeable batteries. *Chem. Rev.* **117**, 13123–13186 (2017). 28960962 10.1021/acs.chemrev.7b00007

[45] Qiao, R., Chin, T., Harris, S. J., Yan, S. & Yang, W. Spectroscopic fingerprints of valence and spin states in manganese oxides and fluorides. *Curr. Appl. Phys.* **13**, 544–548 (2013).

[46] Qiao, R. et al. Revealing and suppressing surface Mn(II) formation of Na0.44MnO2 electrodes for Na-ion batteries. *Nano Energy* **16**, 186–195 (2015).

[47] Xi, L. et al. In situ L-edge XAS study of a manganese oxide water oxidation catalyst. *J. Phys. Chem. C* **121**, 12003–12009 (2017).

[48] Asakura, D. et al. Material/element-dependent fluorescence-yield modes on soft X-ray absorption spectroscopy of cathode materials for Li-ion batteries. *AIP Adv.* **6**, 035105 (2016).

[49] Yamaguchi, A. et al. Regulating proton-coupled electron transfer for efficient water splitting by manganese oxides at neutral pH. *Nat. Commun.* **5**, 4256 (2014). 24977746 10.1038/ncomms5256 PMC4083427

[50] Liu, Y. et al. Photo-stimulated anoxic reduction of birnessite (δ-MnO2) by citrate and its fine structural responses: insights on a proton-promoted photoelectron transfer process. *Chem. Geol.* **561**, 120029 (2021).

[51] Yin, C., Chen, J., Pan, C.-L., Pan, Y. & Hu, J. MOF-derived Mn3O4@C hierarchical nanospheres as cathodes for aqueous zinc-ion batteries. *ACS Appl. Energy Mater.* **5**, 14144–14154 (2022).

[52] Deng, S. et al. Cationic vacancy modulation of Mn3O4 as a superior cathode for durable aqueous zinc-ion batteries. *Adv. Funct. Mater.* **35**, 2413711 (2025).

[53] Wu, L. et al. Unraveling the charge storage mechanism of β-MnO2 in aqueous zinc electrolytes. *ACS Energy Lett.* **9**, 5801–5809 (2024).

[54] Maradesa, A., Py, B., Wan, T. H., Effat, M. B. & Ciucci, F. Selecting the regularization parameter in the distribution of relaxation times. *J. Electrochem. Soc.* **170**, 030502 (2023).

[55] Saccoccio, M., Wan, T. H., Chen, C. & Ciucci, F. Optimal regularization in distribution of relaxation times applied to electrochemical impedance spectroscopy: ridge and lasso regression methods - a theoretical and experimental study. *Electrochimica Acta* **147**, 470–482 (2014).

[56] Wan, T. H., Saccoccio, M., Chen, C. & Ciucci, F. Influence of the discretization methods on the distribution of relaxation times deconvolution: implementing radial basis functions with DRTtools. *Electrochimica Acta* **184**, 483–499 (2015).

[57] Kresse, G. & Hafner, J. Ab initio molecular-dynamics simulation of the liquid-metal–amorphous-semiconductor transition in germanium. *Phys. Rev. B* **49**, 14251–14269 (1994). 10.1103/physrevb.49.14251 10010505

[58] Kresse, G. & Furthmüller, J. Efficient iterative schemes for ab initio total-energy calculations using a plane-wave basis set. *Phys. Rev. B* **54**, 11169–11186 (1996). 10.1103/physrevb.54.11169 9984901

[59] Kresse, G. & Joubert, D. From ultrasoft pseudopotentials to the projector augmented-wave method. *Phys. Rev. B* **59**, 1758–1775 (1999).

[60] Perdew, J. P., Burke, K. & Ernzerhof, M. Generalized gradient approximation made simple. *Phys. Rev. Lett.* **77**, 3865–3868 (1996). 10062328 10.1103/PhysRevLett.77.3865

[61] Kohn, W. & Sham, L. J. Self-consistent equations including exchange and correlation effects. *Phys. Rev.* **140**, A1133–A1138 (1965).

[62] Jain, A. et al. Commentary: The Materials Project: a materials genome approach to accelerating materials innovation. *APL Mater.* **1**, 011002 (2013).

[63] Hjorth Larsen, A. et al. The atomic simulation environment—a Python library for working with atoms. *J. Phys.: Condens. Matter.* **29**, 273002 (2017). 28323250 10.1088/1361-648X/aa680e

[64] Dudarev, S. L., Botton, G. A., Savrasov, S. Y., Humphreys, C. J. & Sutton, A. P. Electron-energy-loss spectra and the structural stability of nickel oxide: An LSDA+U study. *Phys. Rev. B* **57**, 1505–1509 (1998).

