# Supplementary material for: Decoding MnO2 redox chemistry from mechanistic ambiguity to design principles for aqueous Zn-ion batteries
Source: Nat Commun. 2026 Jun 11;17:7433. doi: 10.1038/s41467-026-74350-z (PMC13408579; doi:10.1038/s41467-026-74350-z)
Supplement: Supplementary file 1 — Supplementary Information [file 41467_2026_74350_MOESM1_ESM.pdf]

Decoding MnO_2 Redox Chemistry From Mechanistic Ambiguity to Design Principles for Aqueous Zn-ion Batteries

Yuan Shang[1,], Sankhadip Saha[1,±], Haotian Wen[2,±], Qihui Zhang[1], Xinyuan Wu[3], Bram Hoex[3], Mingyue Wan[4], Nana Wang[5], Tongjun Luo[6], Sougat Purohit[7], Gopalakrishnan Sai Gautam[7], Wesley M. Dose[6], Lars Thomsen[8], Shery Chang[2,*] Priyank Kumar[9,*], Dipan Kundu[1,*]*

[1]*LBRI, School of Chemical Engineering, UNSW Sydney, Kensington, NSW 2052, Australia*

[2]*School of Materials Science and Engineering, UNSW Sydney, Kensington, NSW 2052, Australia*

[3]*School of Photovoltaic and Renewable Energy Engineering, UNSW Sydney, Kensington, NSW 2052, Australia*

[4]*Institute for Superconducting and Electronic Materials, Faculty of Engineering and Information Sciences, University of Wollongong, North Wollongong, NSW 2500, Australia*

[5]*Centre for Clean Energy Technology, School of Mathematical and Physical Sciences, Faculty of Science, University of Technology Sydney, Sydney, NSW, 2007 Australia*

[6]*School of Chemistry, The University of Sydney, Sydney, NSW 2006, Australia*

[7]*Department of Materials Engineering, Indian Institute of Science, Bengaluru, 560012, India*

[8]*Australian Synchrotron, ANSTO, Clayton, VIC, 3168 Australia*

[9]*School of Chemical Engineering, UNSW Sydney, Kensington, NSW 2052, Australia*

±Equal contributions

**Corresponding author*

Emails:

Yuan Shang; *yuan.shang@unsw.edu.au*,

Shery Chang; *shery.chang@unsw.edu.au*,

Priyank Kumar; *priyank.kumar@unsw.edu.au*,

Dipan Kundu; *d.kundu@unsw.edu.au*

Supplementary Note 1

$$\mathbf{1st\ discharge}: \ MnO_2(s) + H^+ \rightarrow MnO_2(s) - H^+(ads) \ \rightarrow \ Mn^{2+} + \ H_2O\ (l) \qquad (1)$$

$$(chemical\ adsorption\ of\ proton - chemical\ dissolution)$$

$$MnO_2(s) - H^+(ads) + 3H^+ + 2e^- \rightarrow Mn^{2+}(aq) + 2H_2O(l) \qquad (2)$$

$$MnO_2(s) + xH^+ + xe^- \rightarrow H_xMnO_2(s) \qquad (3)$$

$$4Zn^{2+}(aq) + SO_4{}^{2-}(aq) + 5H_2O(l) \ + \ 6OH^-(aq) \rightarrow Zn_4SO_4(OH)_6 \cdot 5H_2O(s) \qquad (4)$$

$$\mathbf{1st\ charge}: \qquad H_xMnO_2(s) \ \rightarrow \ MnO_2(s) + xH^+ \ + xe^- \qquad (5)$$

$$Mn^{2+}(aq) + xH_2O(l) \rightarrow MnOx(s) + 2xH^+ + (2x-2)e^- \qquad (6)$$

$$(Mixture\ Mn^{3+} and\ Mn^{2+}\ in\ MnOx)$$

$$Zn_4SO_4(OH)_6 \cdot 5H_2O(s) + 6H^+ \rightarrow \ 4Zn^{2+}(aq) + SO_4{}^{2-}(aq) + 11H_2O(l) \qquad (7)$$

$$\mathbf{2nd\ discharge\ onward}:$$

$$Mn^{3+} reduction - 1st\ plateau$$

$$MnO_x(s) + 2xH^+ + (2x-2)e^- \ \rightarrow Mn^{2+}(aq) + xH_2O(l) \qquad (8)$$

$$Pristine\ MnO_2\ reduction - 2nd\ plateau$$

$$MnO_2(s) + H^+ \rightarrow MnO_2(s) - H^+(ads) \ \rightarrow \ Mn^{2+} + \ H_2O\ (l) \qquad (9)$$

$$(chemical\ adsorption\ of\ proton - chemical\ dissolution)$$

$$MnO_2(s) - H^+(ads) + 3H^+ + 2e^- \rightarrow Mn^{2+}(aq) + 2H_2O(l) \qquad (10)$$

$$MnO_2(s) + xH^+ \rightarrow H_xMnO_2(s) \qquad (11)$$

$$MnO_x(s) + yH^+ \rightarrow H_yMnO_x(s) \qquad (12)$$

$$4Zn^{2+}(aq) + SO_4{}^{2-}(aq) + 5H_2O(l) \ + \ 6OH^-(aq) \rightarrow Zn_4SO_4(OH)_6 \cdot 5H_2O(s) \qquad (13)$$

$$\mathbf{2nd\ charge\ onward}: \ Mn^{2+}(aq) + xH_2O(l) \rightarrow MnOx(s) + 2xH^+ + (2x-2)e^- \qquad (14)$$

$$H_xMnO_2(s) \ \rightarrow \ MnO_2(s) + xH^+ \qquad (15)$$

$$H_yMnO_x(s) \ \rightarrow \ MnO_x(s) + yH^+ \qquad (16)$$

$$Zn_4SO_4(OH)_6 \cdot 5H_2O(s) + 6H^+ \rightarrow \ 4Zn^{2+}(aq) + SO_4{}^{2-}(aq) + 11H_2O(l) \qquad (17)$$

Table S1. The summary of Mn L_3 and Mn L_2 peak position and ΔE for pristine electrode and different regions of α-MnO_2 during 1st cycle.

Sample	Mn-L_3	Mn-L_2	ΔE between L_3 and L_2
Pristine	642.1 eV	652.6 eV	10.5 eV
1st discharge-inner region	641.9 eV	652.4 eV	10.5 eV
1st discharge-surface region	640.0 eV	652.0 eV	12.0 eV
1st charge-inner region	642.2 eV	652.8 eV	10.3 eV
1st charge-surface region	641.9 eV	652.5 eV	10.6 eV
1st charge-deposited phase	639.8 eV	651.7	11.9 eV

Table S2. The summary of Mn L_3 and Mn L_2 peak position and ΔE for pristine electrode and different regions of α-MnO_2 during 2nd cycle.

Sample	Mn-L_3	Mn-L_2	ΔE between L_3 and L_2
Pristine	642.1 eV	652.6 eV	10.5 eV
2nd discharge-inner region	642.2 eV	652.5 eV	10.3 eV
2nd discharge-surface region	641.2 eV	652.8 eV	11.6 eV
2nd charge-inner region	642.4 eV	652.8 eV	10.4 eV
2nd charge-surface region	642.4 eV	652.6 eV	10.2 eV
2nd charge-deposited phase	640.4 eV	652.1 eV	11.7 eV

Table S3. The summary of Mn L3 and Mn L2 peak position and ΔE for pristine electrode and different regions of α-MnO_2 during 20th cycle.

Sample	Mn-L_3	Mn-L_2	ΔE between L_3 and L_2
Pristine	642.1 eV	652.6 eV	10.5 eV
20th discharge-inner region	642.5 eV	652.9 eV	10.3 eV
20th discharge-surface region	640.6 eV	652.3 eV	11.7 eV
20th discharge-deposited phase	640.2 eV	651.6 eV	11.4 eV
20th charge-inner region	642.5 eV	653.2 eV	10.7 eV
20th charge-surface region	641.9 eV	653.0 eV	11.1 eV
20th charge-deposited phase	640.8 eV	651.6 eV	10.8 eV

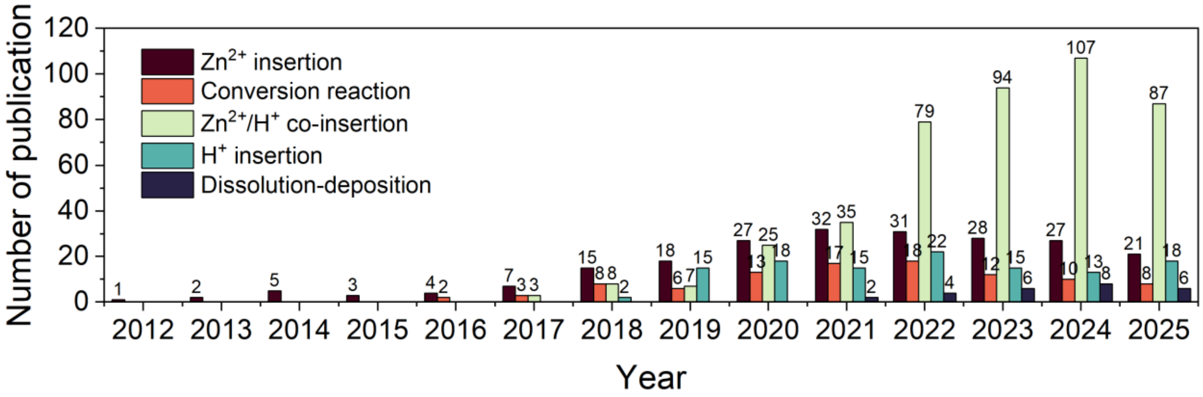

Fig. S1. Annual number of publications (2012-2025) on aqueous Zn||MnO_2 batteries classified by the dominant charge-storage mechanism proposed.

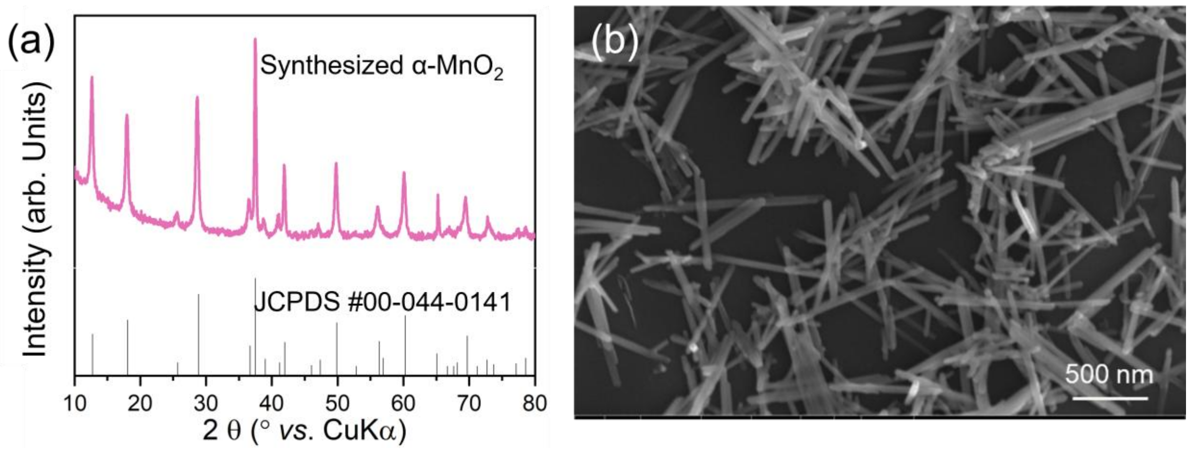

Fig. S2. (a) The XRD pattern of the as-synthesized α-MnO_2. All peaks are well aligned with the tetragonal structure (JCPDS #00-044-0141), corroborating a high purity of the synthesized material. (b) A typical SEM image of the as-synthesized α-MnO_2.

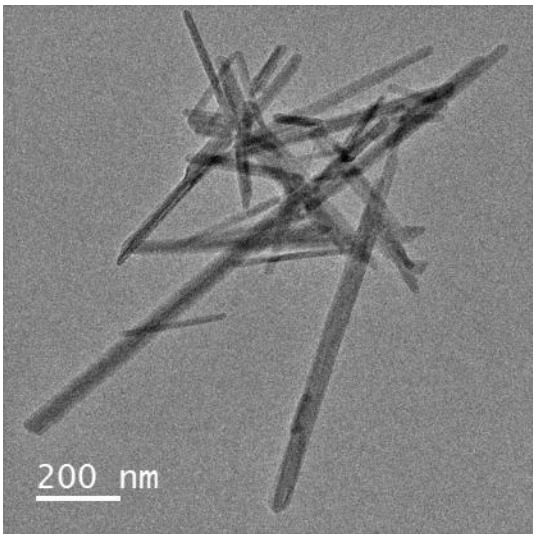

Fig. S3. A representative TEM image of the as-synthesized α-MnO_2 nanorod.

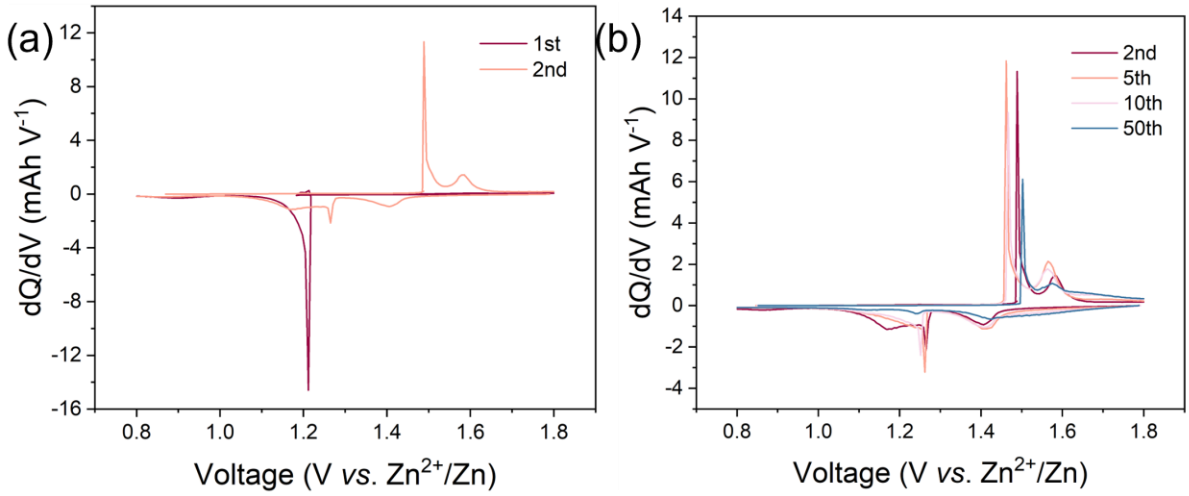

Fig. S4. Differential charge-discharge capacity versus potential (dQ/dV) profile for α-MnO_2 electrode during (a) first two cycles and (b) following cycles.

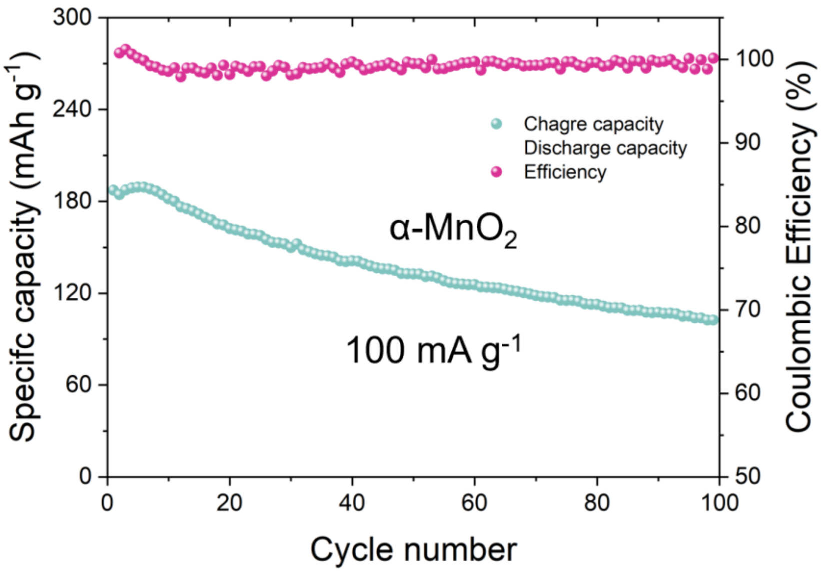

Fig. S5. The long-term cyclability evaluation for α-MnO_2 cathode at 100 mA g^{-1}.

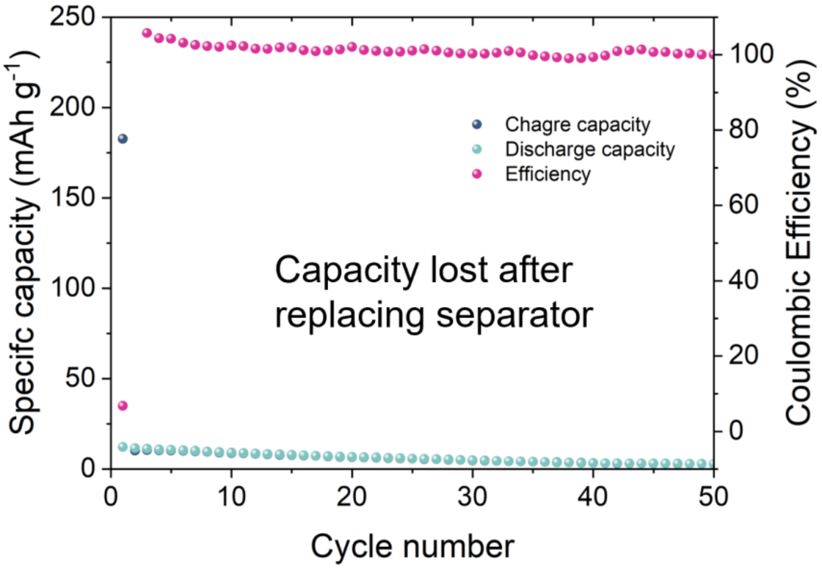

Fig. S6. Zn||MnO_2 cell capacity evolution with cycling after the separator replacement.

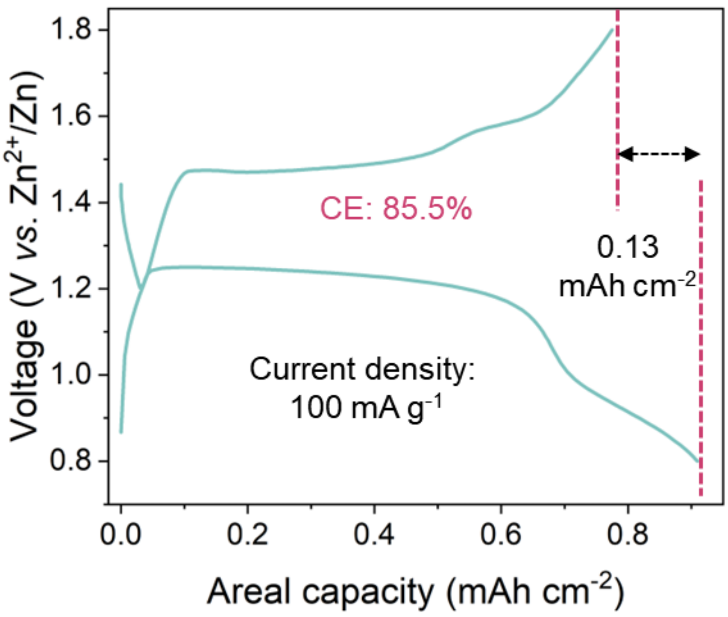

Fig. S7. The 1st cycle GCD profile of the Zn||MnO_2 cell used for ICP-OES analysis of the electrolyte following the first discharge and charge.

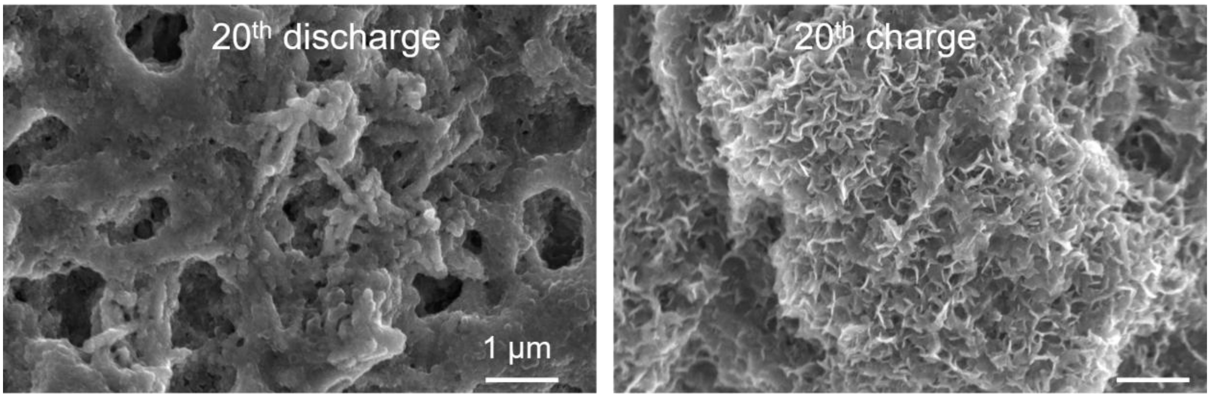

Fig. S8. The SEM images of the α-MnO_2 electrode after 20th discharge and charge.

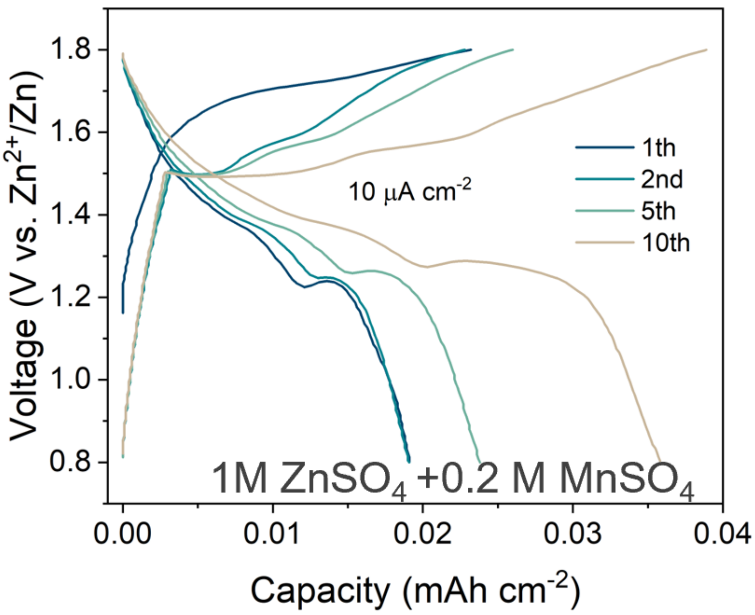

Fig. S9. GCD profile of graphite-Zn cell using 1M $ZnSO_4$-0.2 M $MnSO_4$ electrolyte at a current of 10 μA cm^{-2}.

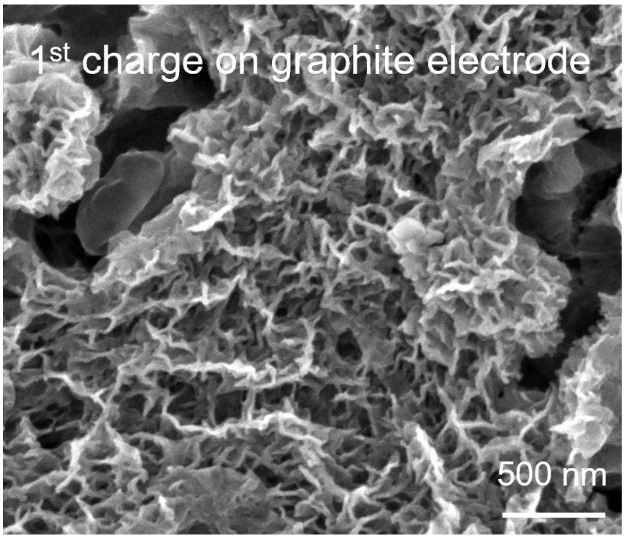

Fig. S10. The SEM image of the graphite electrode after 1st charge with 1M $ZnSO_4$-0.2 M $MnSO_4$ electrolyte at a current of 10 μA cm^{-2}.

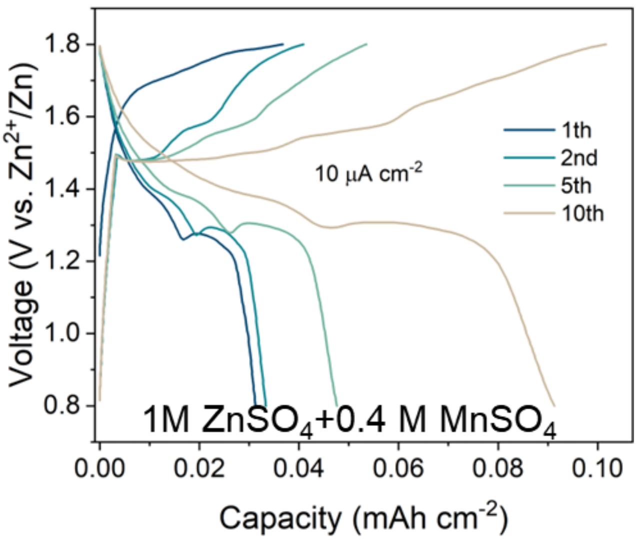

Fig. S11. GCD profile for the graphite-Zn cell with 1M $ZnSO_4$-0.4 M $MnSO_4$ electrolyte at a current of 10 μA cm^{-2}.

The Zn||graphite cell demonstrates voltage profiles similar to those of Zn||MnO_2 after the first cycle (**Fig. S9** and **Fig. 1d**), accompanied by comparable nanosheet morphology formed upon charging (**Fig. S10**). The amount of $MnSO_4$ (0.2 M) in the electrolyte for the Zn||graphite cell is not fully utilized in the deposition reaction, as it only exhibits a capacity of 0.018 mAh cm^{-2} in the 1st discharge (**Fig. S9**). Even when $MnSO_4$ increases to 0.4 M, the capacity is still substantially low (**Fig. S11**). Intriguingly, it should be noted that the capacity gradually increases in the following cycles for both $MnSO_4$-containing electrolytes. It is likely that with progressive coverage of the deposited nanosheets on the graphite substrate, these deposits function as a seed and subsequently promote further Mn^{2+} oxidation.

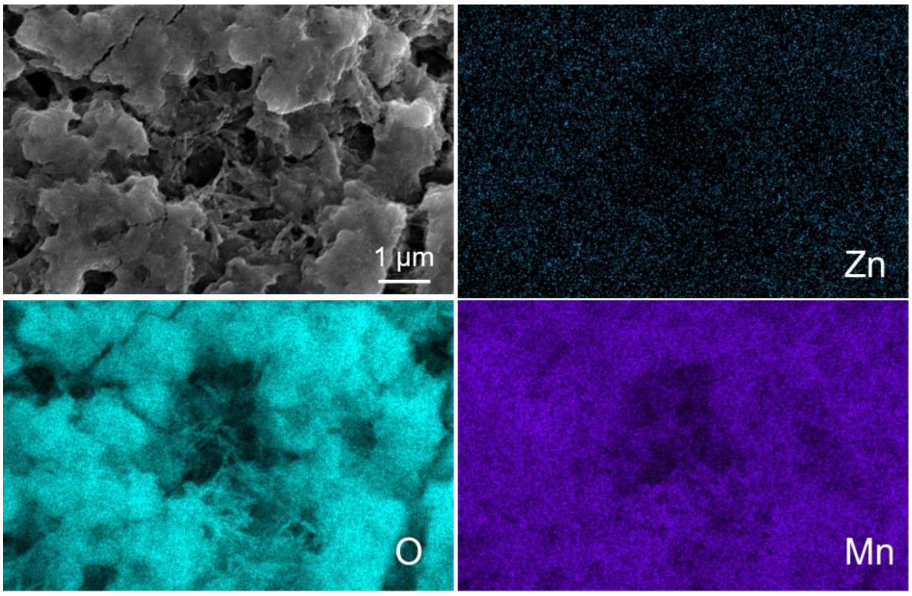

Fig. S12. SEM-EDS mappings of α-MnO_2 electrode after 20th discharge.

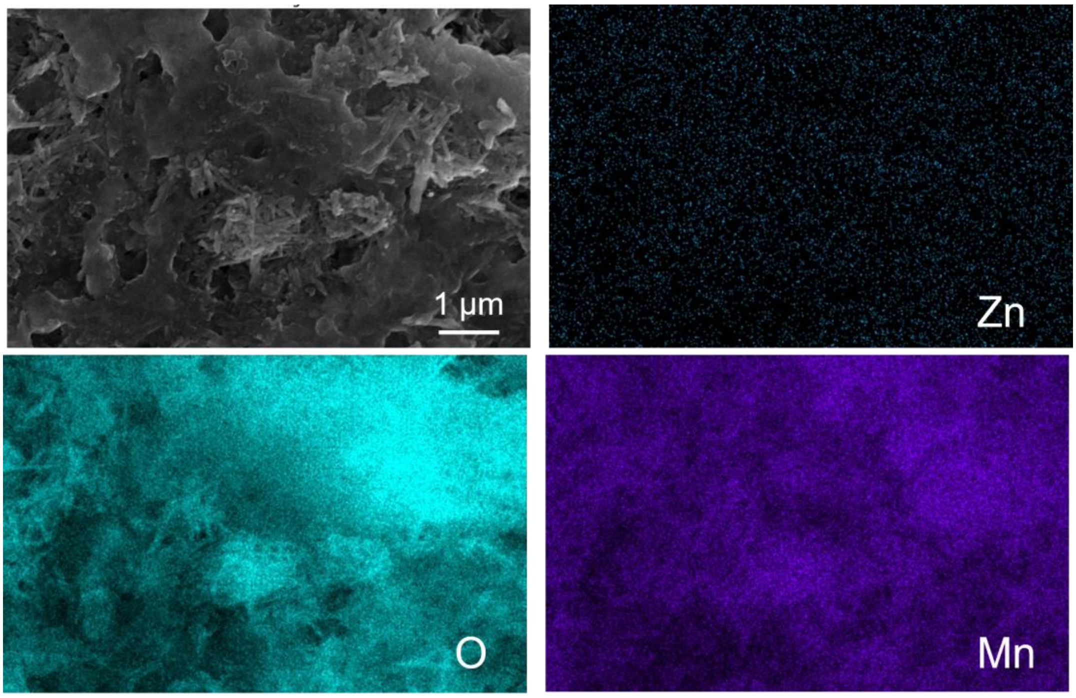

Fig. S13. SEM-EDS mappings of α-MnO_2 electrode after 20th charge.

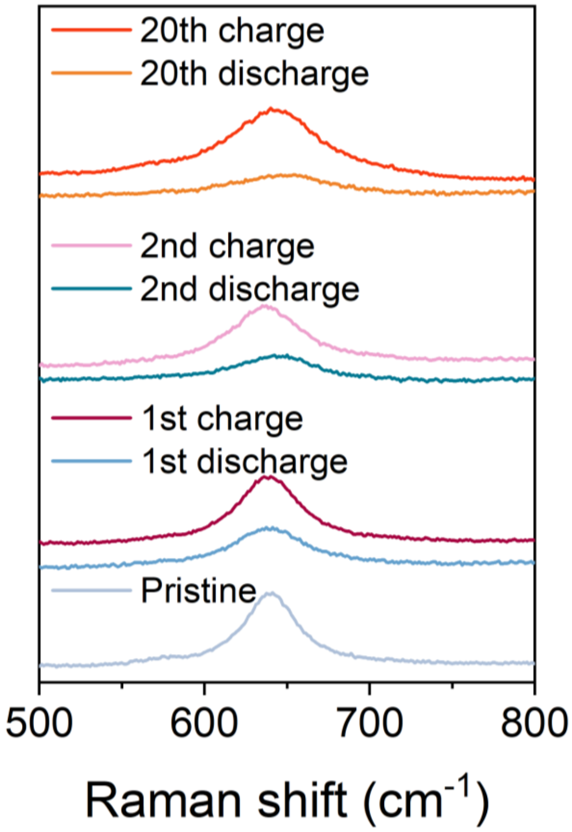

Fig. S14. The ex-situ Raman investigation of the α-MnO_2 electrode at various cycling stages.

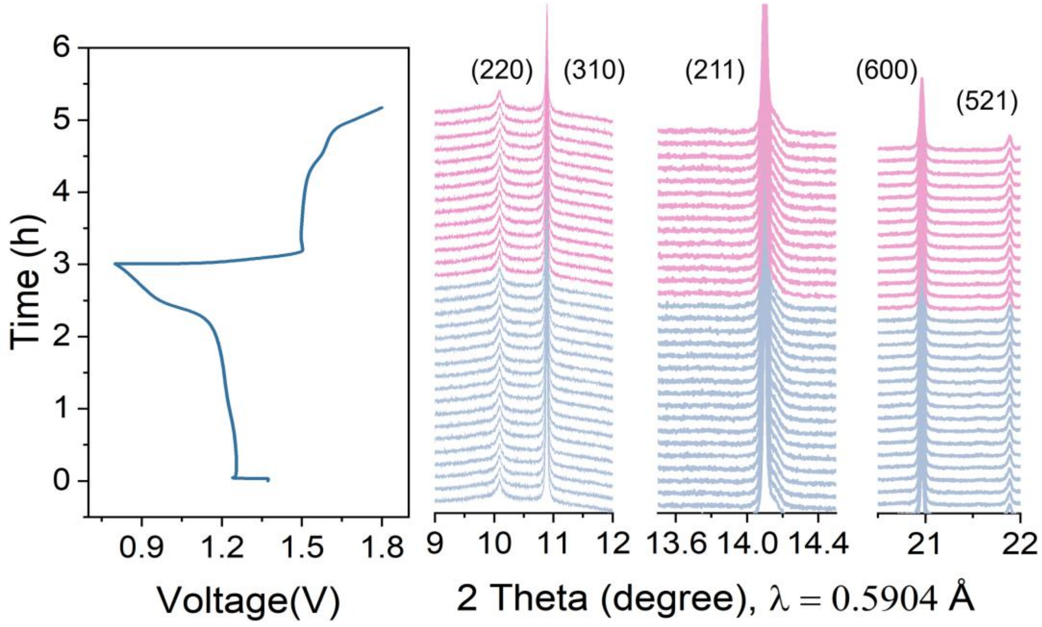

Fig. S15. Operando synchrotron XRD showing the phase evolution of the α-MnO_2 during the 1st cycle.

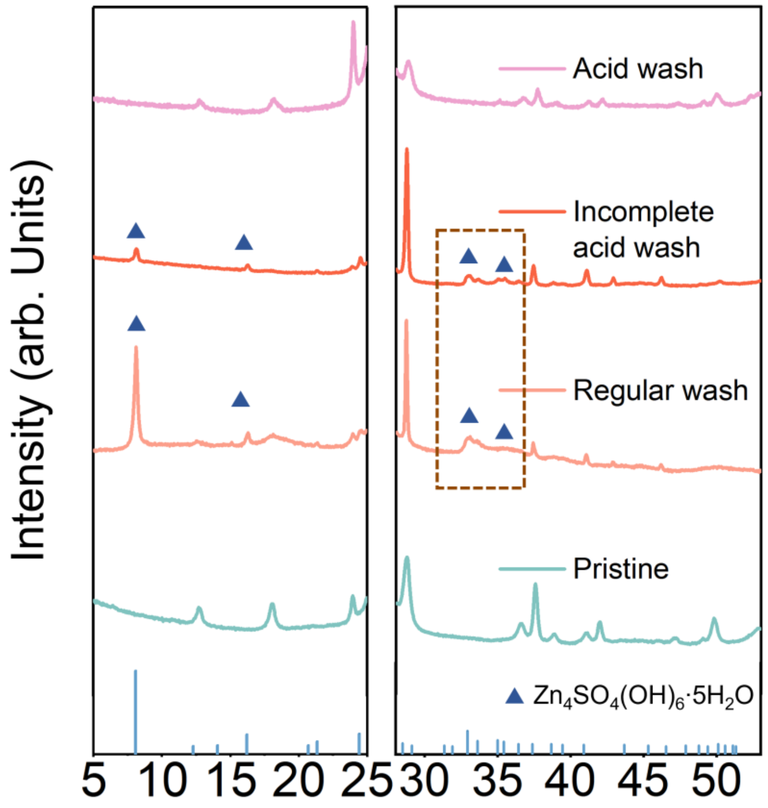

Fig. S16. Comparison of XRD patterns of the discharged α-MnO_2 after different washing treatments.

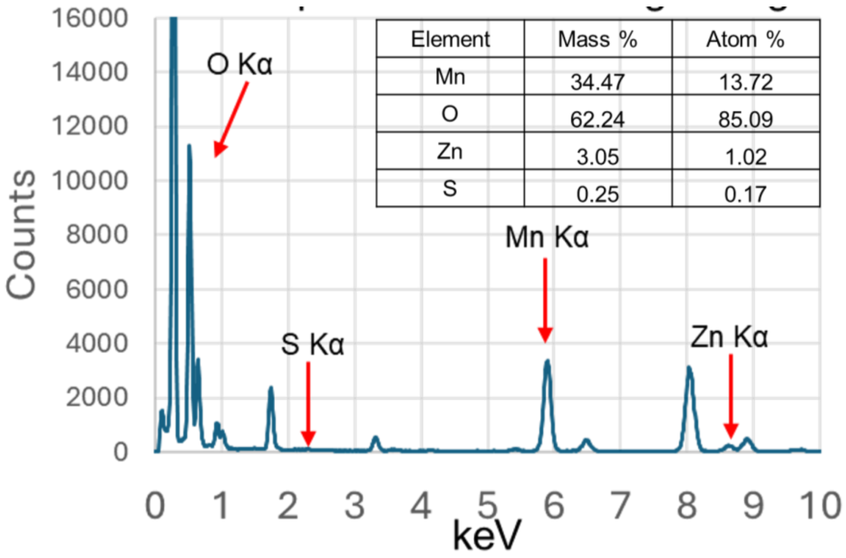

Fig. S17. The STEM-EDS spectrum of the α-MnO_2 nanorods after 1st discharge.

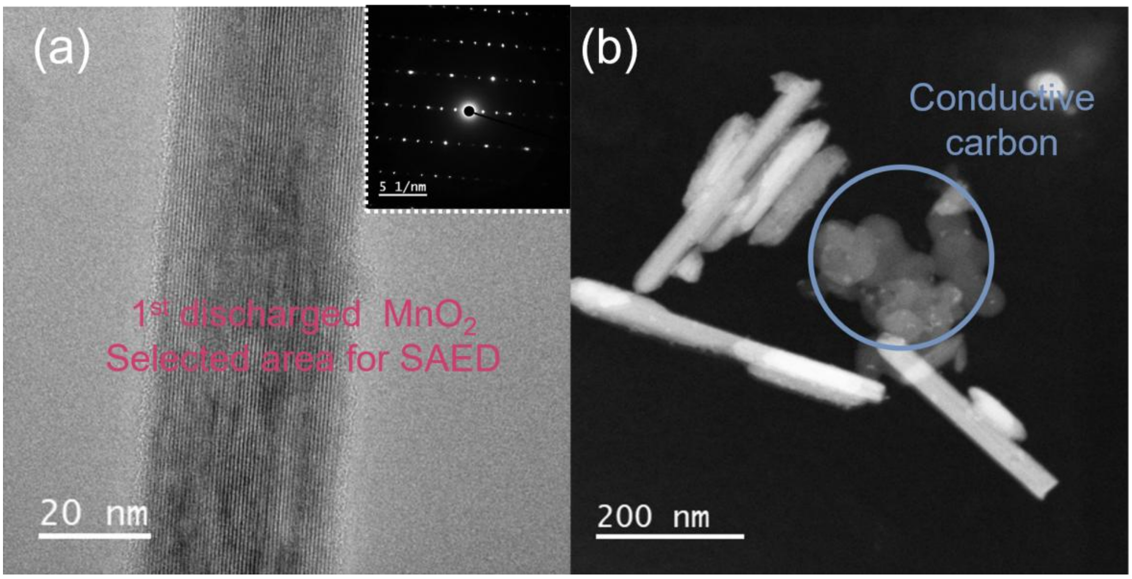

Fig. S18. (a) A representative TEM image of the α-MnO_2 nanorod after the 1st discharge with the SAED pattern shown in the inset. (b) Low-magnification TEM image of the 1st discharged MnO_2 electrode.

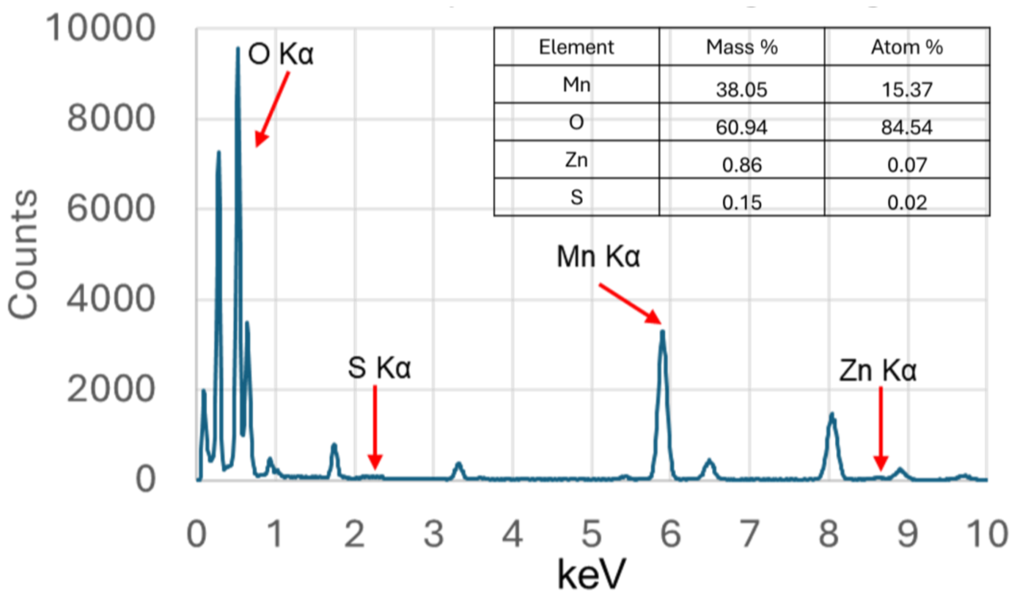

Element	Mass %	Atom %
Mn	38.05	15.37
O	60.94	84.54
Zn	0.86	0.07
S	0.15	0.02

Fig. S19. The STEM-EDS spectrum of the α-MnO_2 nanorods after 1st charge.

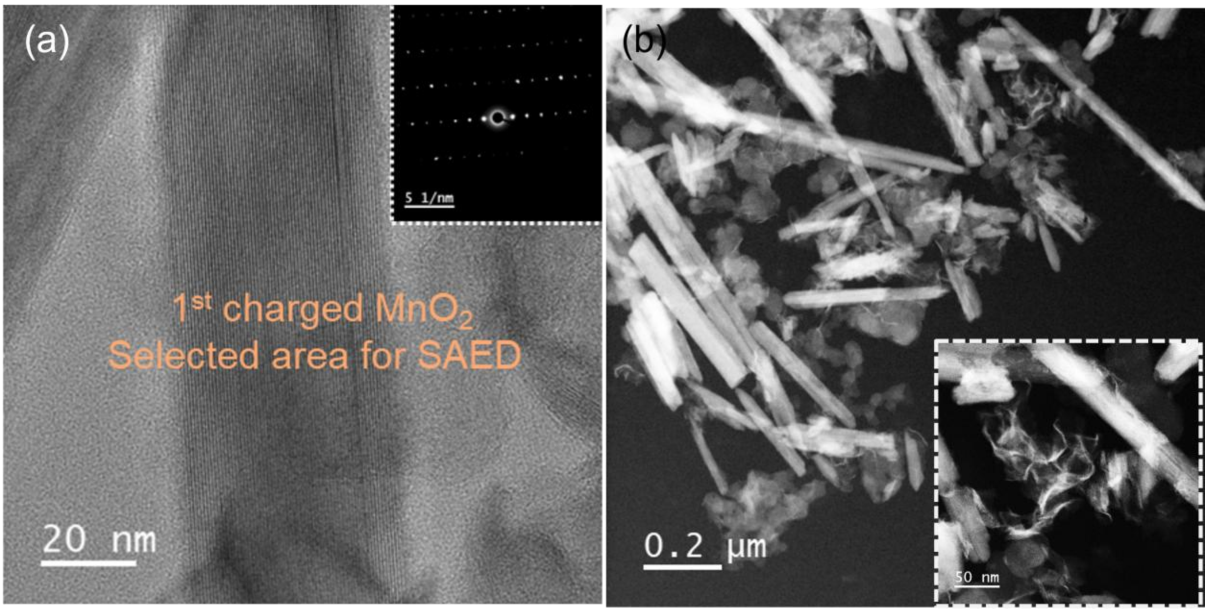

Fig. S20. (a) A representative TEM image of the α-MnO_2 nanorod with the SAED pattern shown in the inset. (b) Low-magnification TEM image of the charged (1st) MnO_2 electrode.

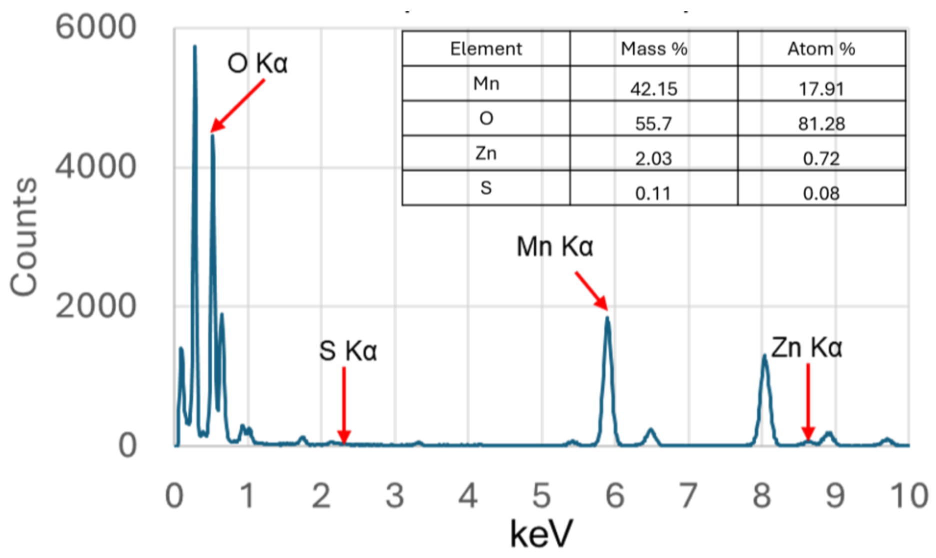

Fig. S21. The STEM-EDS spectrum of deposited phase after 1st charge.

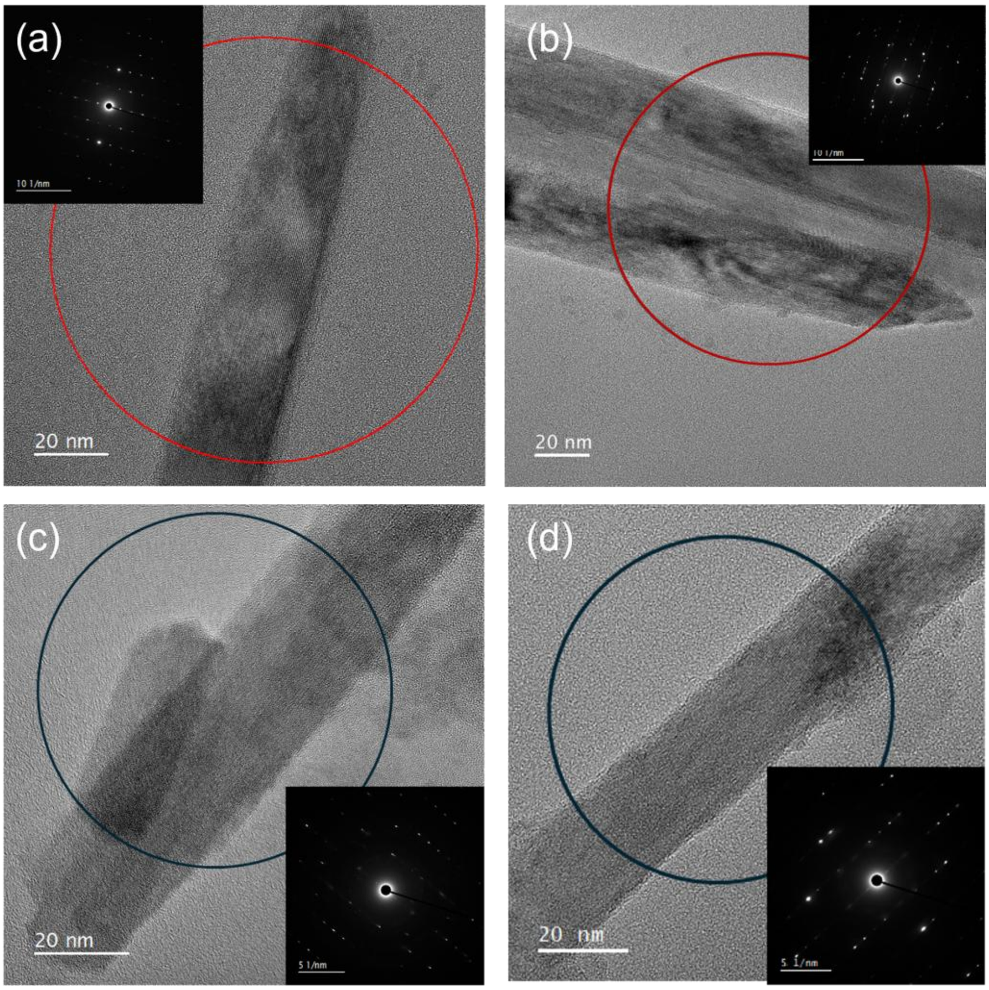

Fig. S22. Representative TEM image of the α-MnO_2 nanorod after (a) 2nd discharge, (b) 2nd charge, (c) 20th discharge and (d) 20th charge, the area selected for SAED analysis is highlighted by the circle and the corresponding SAED pattern is shown in the inset.

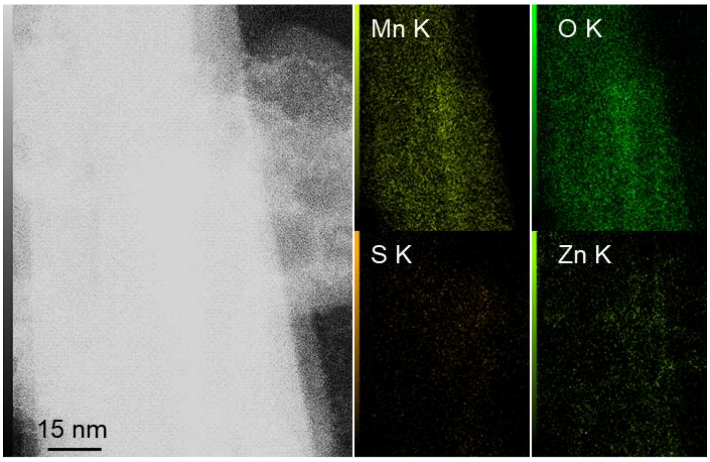

Fig. S23. STEM–EDS analysis on MnO_2 nanorod after 2nd discharge.

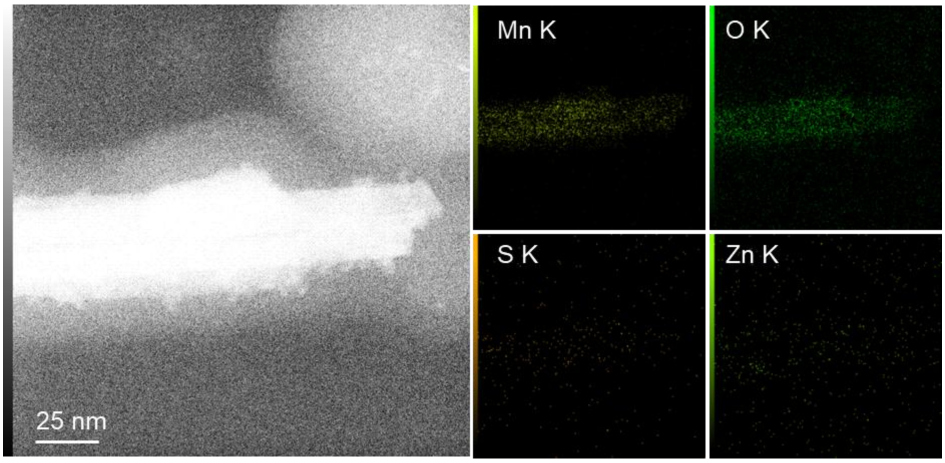

Fig. S24. STEM–EDS analysis on MnO_2 nanorod after 2nd charge.

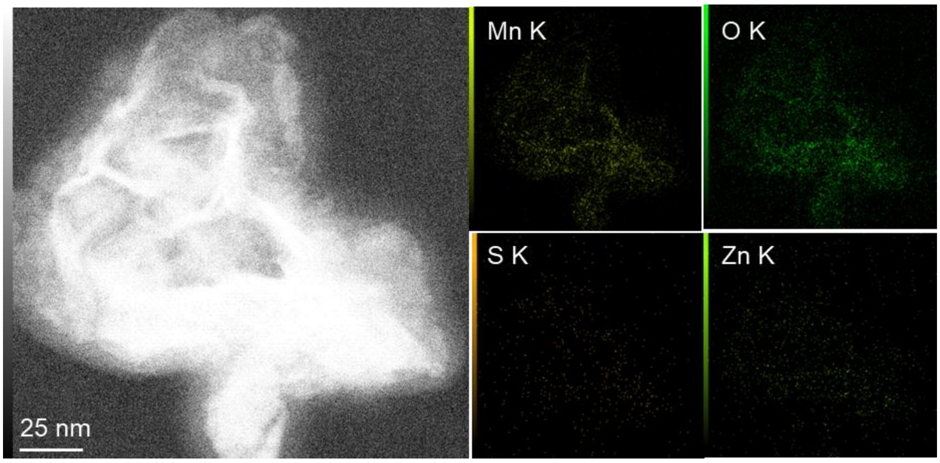

Fig. S25. STEM–EDS analysis of the deposited nanosheet after 2nd charge.

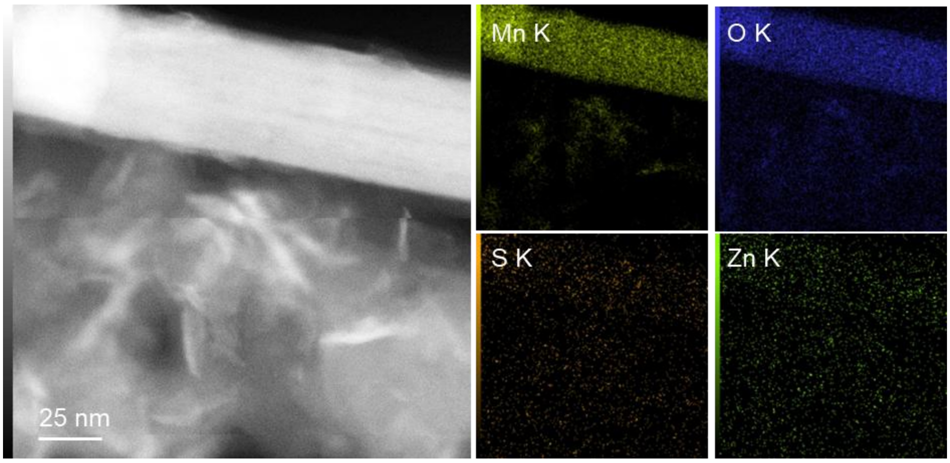

Fig. S26. STEM–EDS analysis on the MnO_2 nanorod and surrounding nanosheets after 20th discharge.

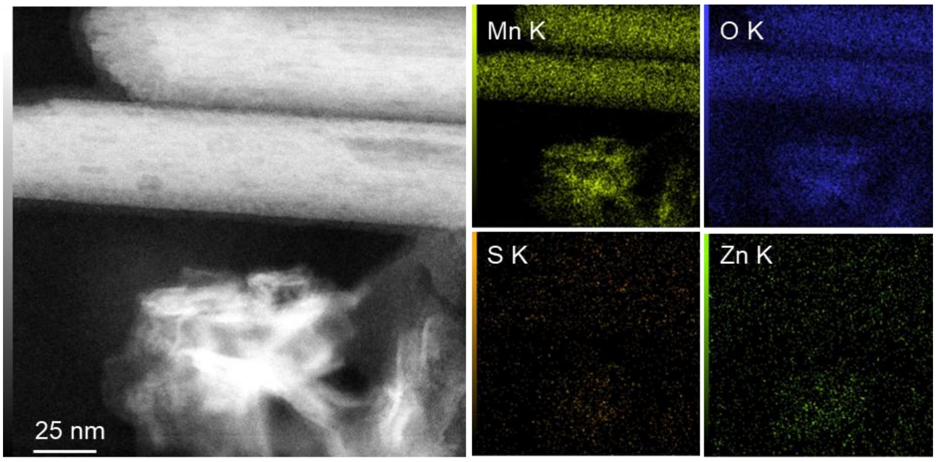

Fig. S27. STEM–EDS analysis on the MnO_2 nanorod and surrounding nanosheets after 20th charge.

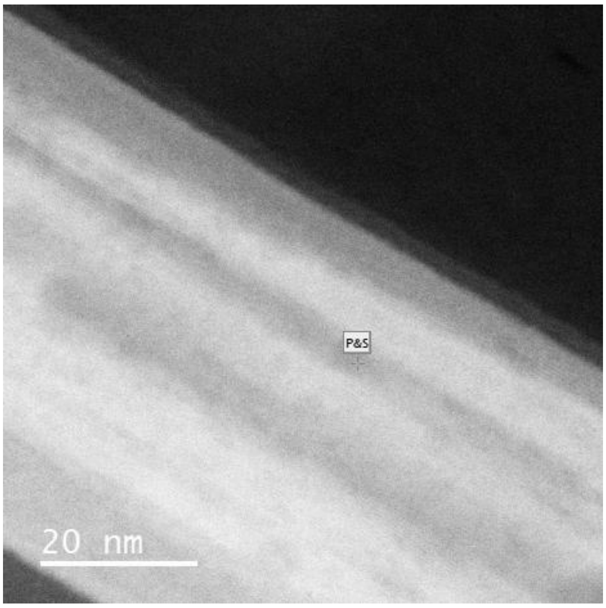

Fig. S28. Selected pristine MnO_2 nanorod for EELS analysis.

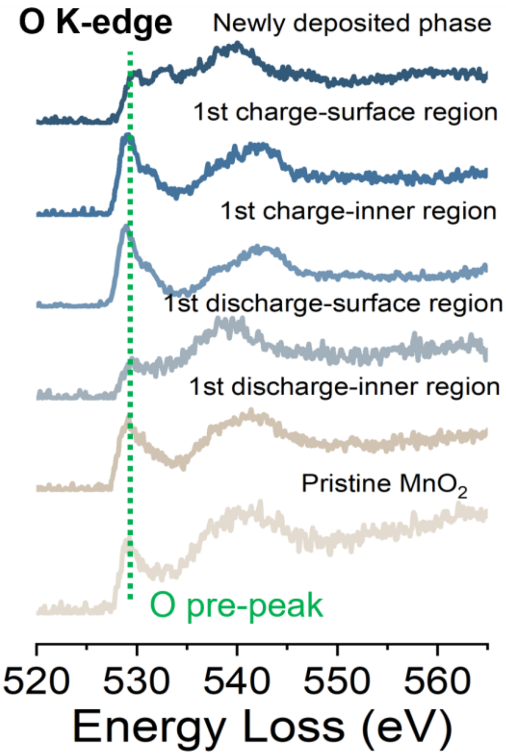

Fig. S29. EELS O K-edge spectra evolution as a function of discharge/charge during the first cycle.

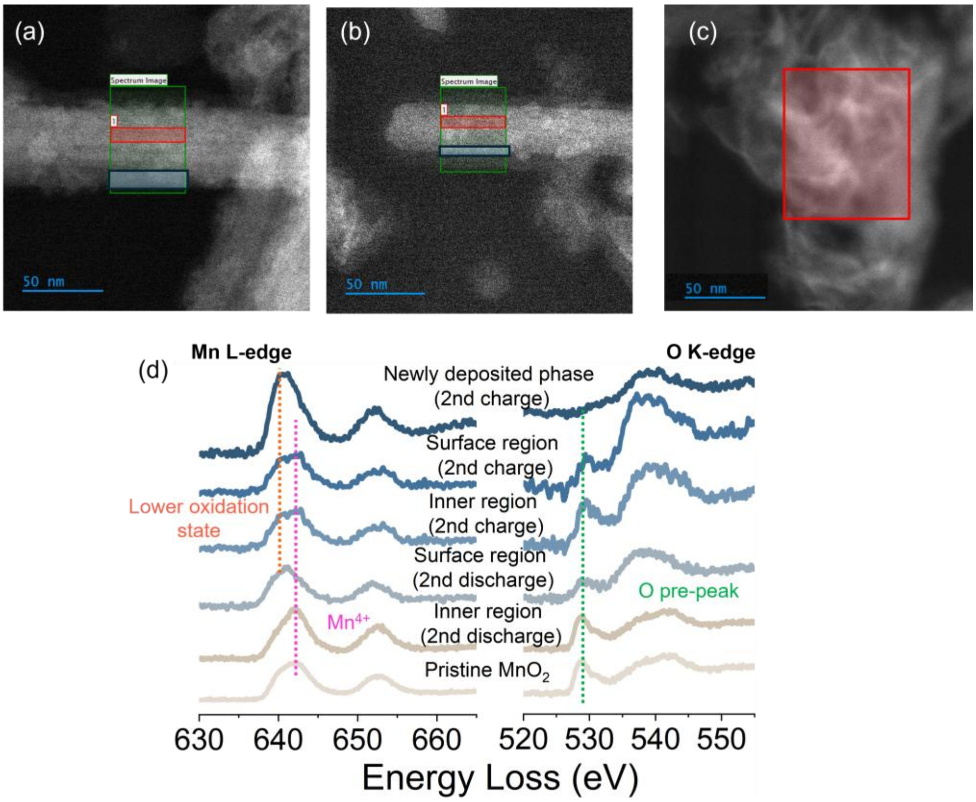

Fig. S30. Low-magnification TEM for (a) 2nd discharged and (b) 2nd charged MnO_2 nanorod and (c) the deposited phase formed during 2nd charge. (d) The EELS Mn L-edge and O K-edge spectra collected from the labelled region in a-c.

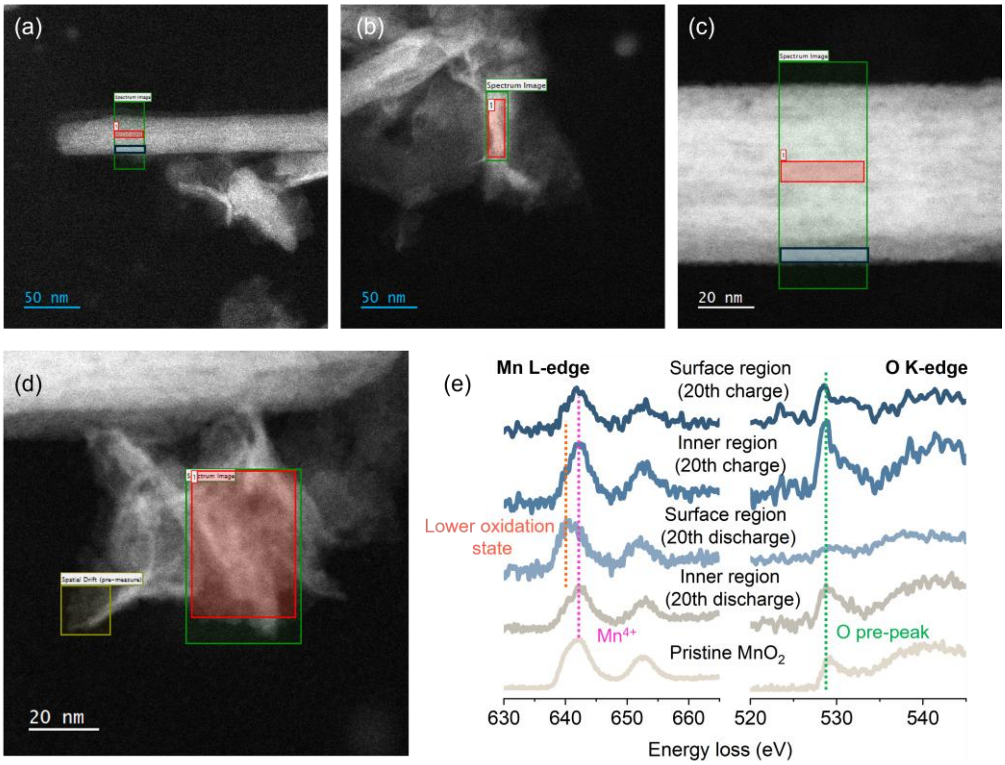

Fig. S31. The low-magnification TEM for (a) 20th discharged and (c) 20th charged MnO_2 nanorod and deposited phase formed during (b) 20th discharge and (d) charge. (e) The EELS Mn L-edge and O K-edge spectra collected from the labelled region in a-c.

The Mn L-edge spectra over subsequent cycles (2nd and 20th discharge/charge) exhibit trends consistent with the first cycle, with the surface oxidation state of MnO_2 nanorods reversibly decreasing upon discharge and increasing upon charge. The evolution of the peak intensity in O K-edge pre-peak also mirrors the trend in the 1st cycle; however, after long-term cycling (20th discharge, surface region), the pre-peak intensity completely vanishes, evidencing severe surface degradation and a highly defective structure induced by repeated intercalation and deintercalation.

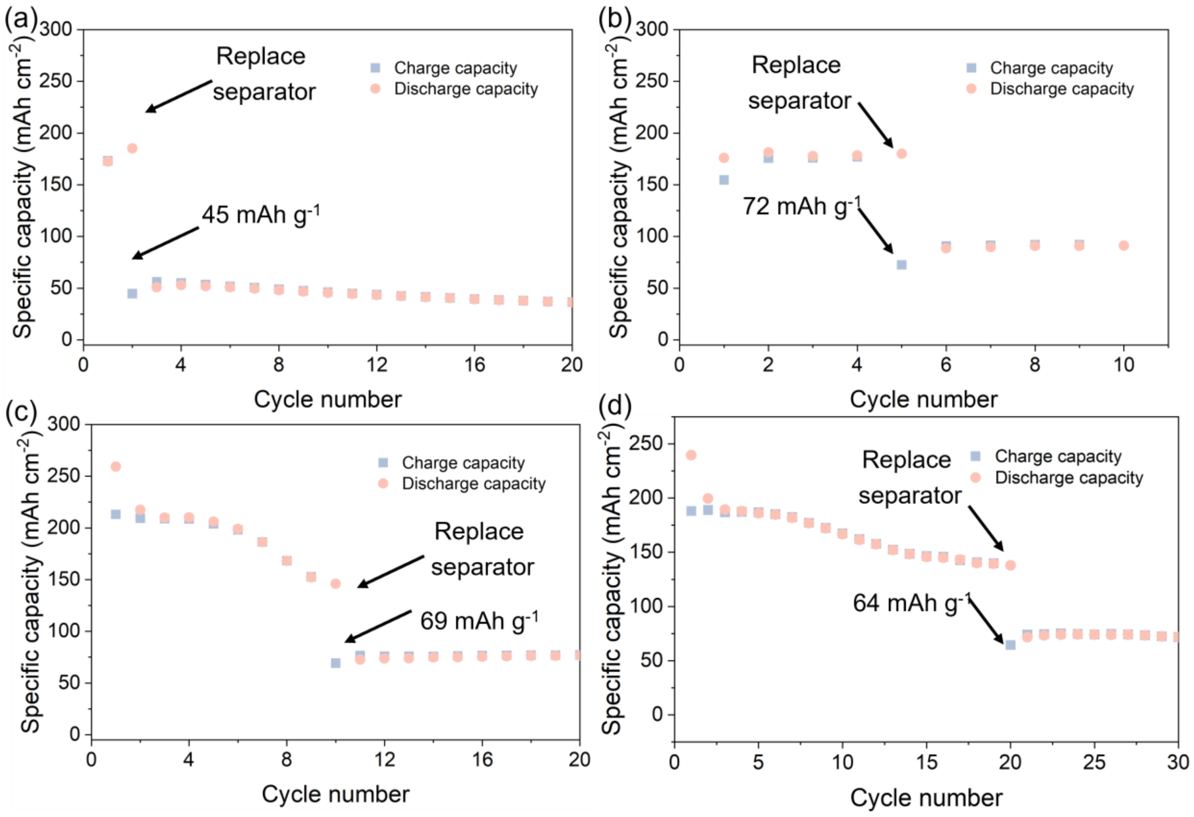

Fig. S32. The long-term cyclability of Zn||MnO_2 cell before and after replacing the separator after (a) 2nd, (b) 5th, (c) 10th and (d) 20th cycle.

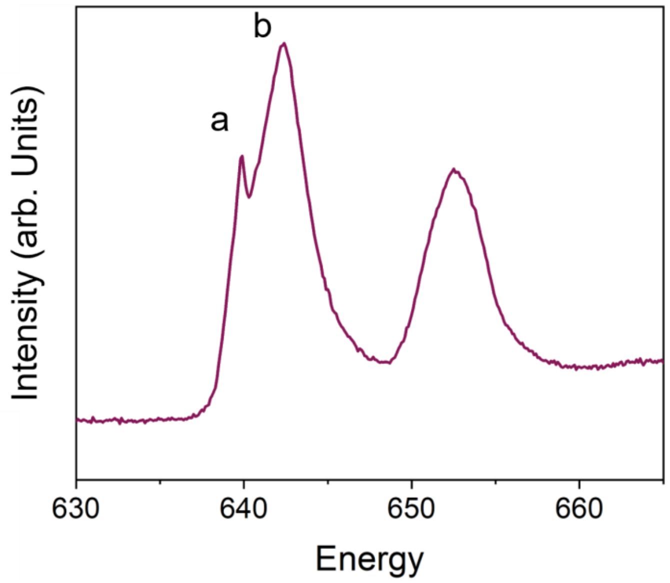

Fig. S33. Soft XAS Mn-L edge of pristine α-MnO_2 electrode collected in AEY mode. The two L_3 peaks, a and b, are identified as characteristic peaks for Mn^{4+}, arising from transitions to orbitals with t_{2g} and e_g, respectively.[1]

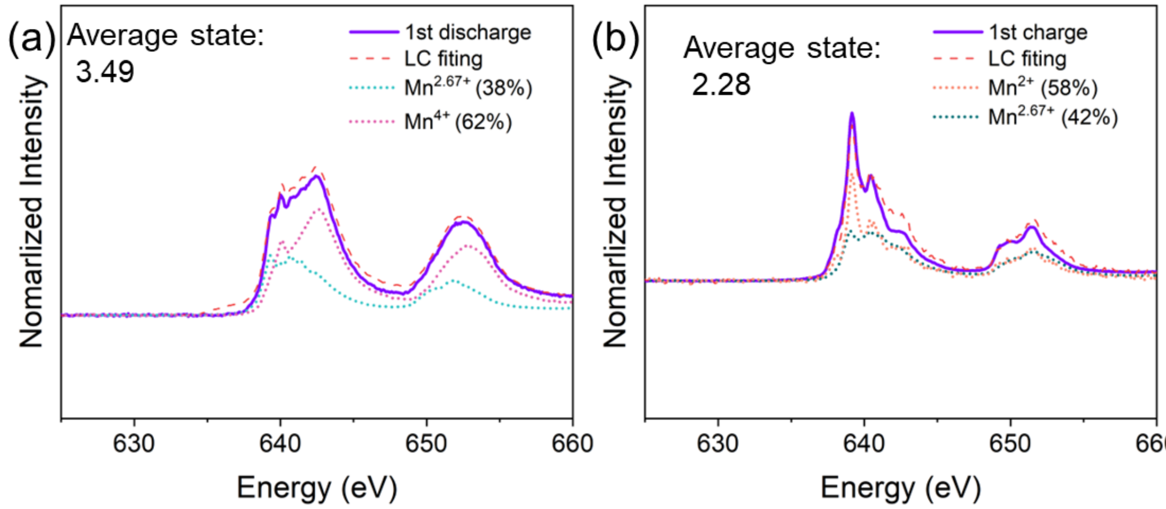

Fig. S34. The linear combination fitting of Mn L-edge AEY spectrum for α-MnO_2 electrode after (a) 1st discharge (b) 1st charge. Although the fitted average Mn valence of the deposited material is close to that of Mn_3O_4, this does not imply a spinel structure; TEM and XRD indicate an amorphous/disordered MnO_x.

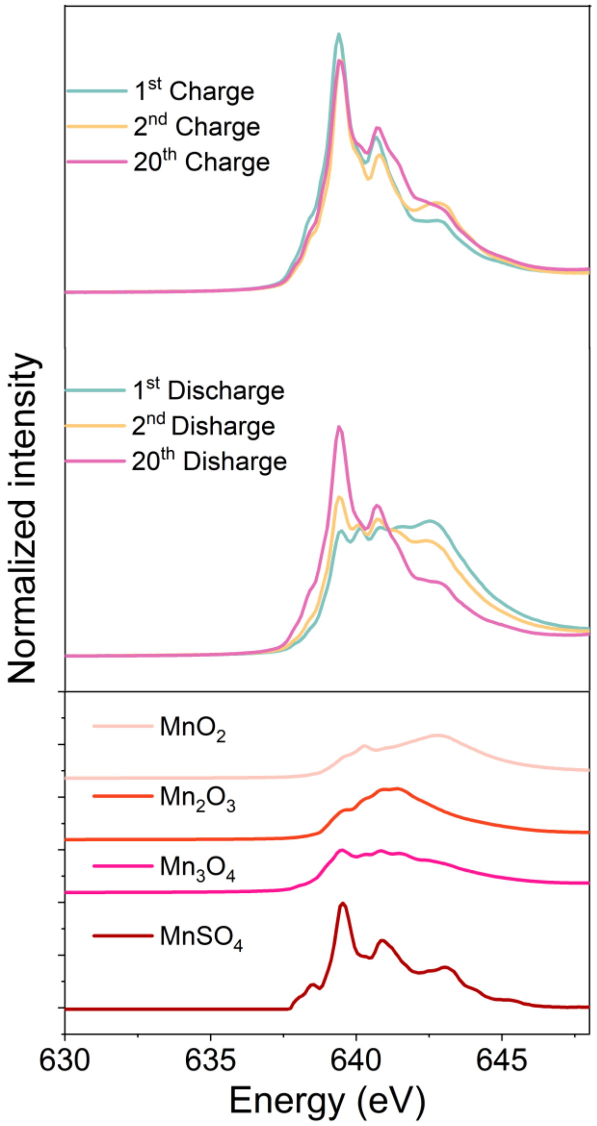

Fig. S35. The soft XAS Mn L-edge spectra collected in the TEY mode for different discharge and charge electrodes.

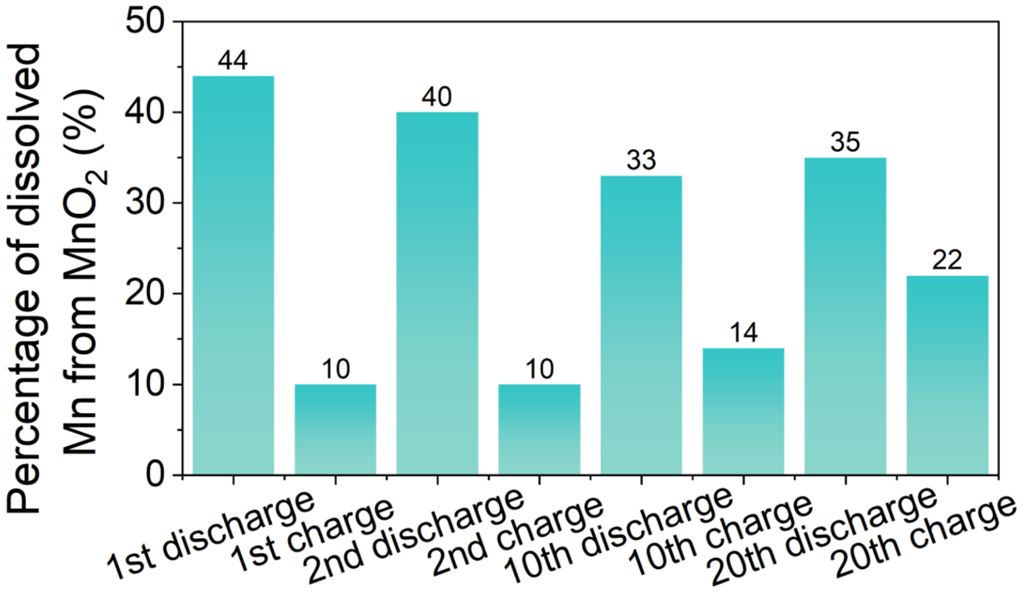

Fig. S36. The percentage of dissolved Mn (using pristine MnO_2 electrode mass as reference) in the electrolyte as measured by ICP-OES at different discharge/charge points during cycling.

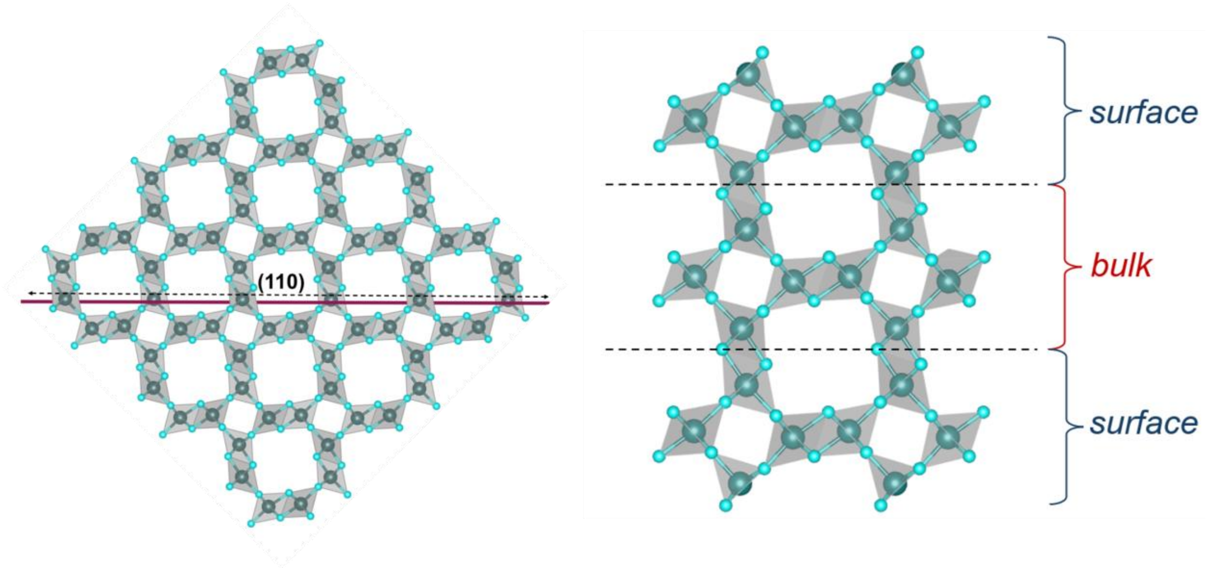

Fig. S37. Construction of the slab for the α-MnO_2 unit cell, specifying both the surface and bulk.

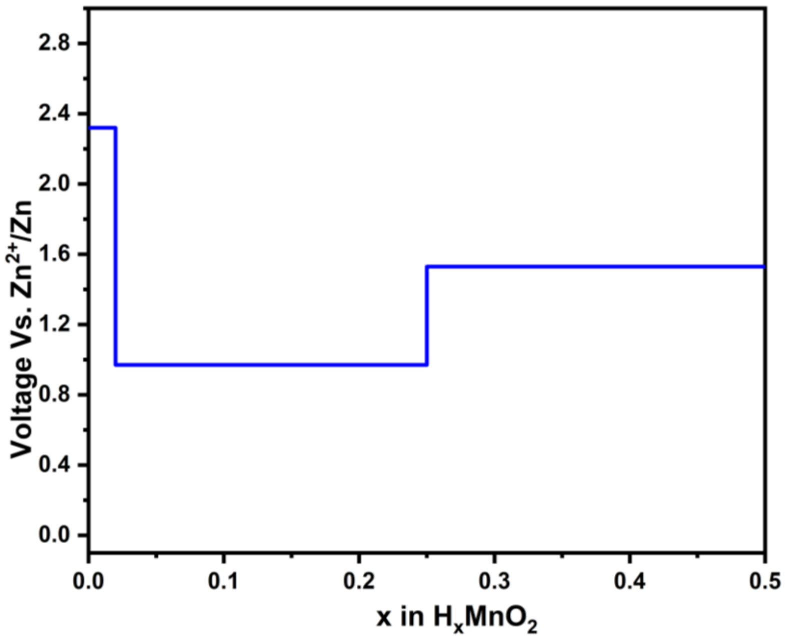

Fig. S38. Voltage–composition profile from a (pseudo-)binary grand-potential convex hull of H_xMnO_2 (0< x< 0.5).

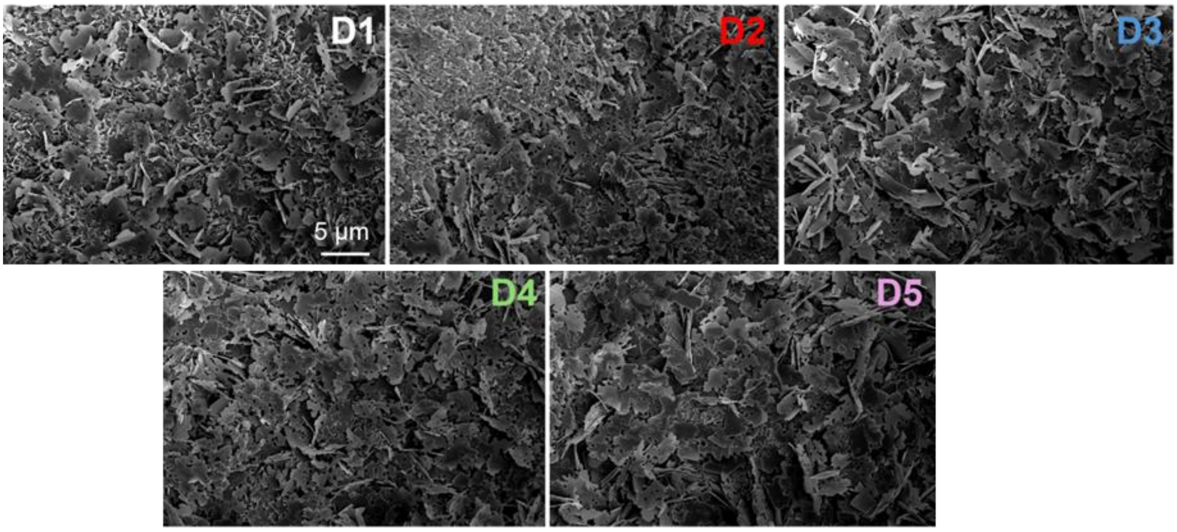

Fig. S39.The SEM images of α-MnO_2 at different discharged states without acid wash.

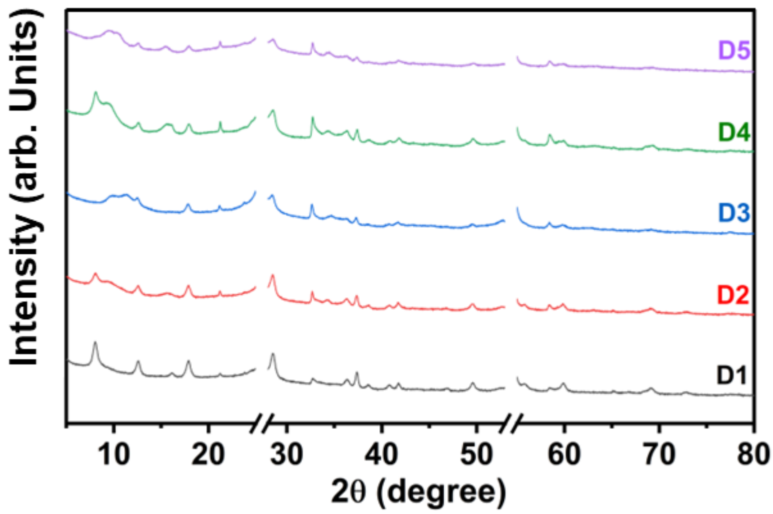

Fig. S40. The XRD patterns of α-MnO_2 at different discharged states.

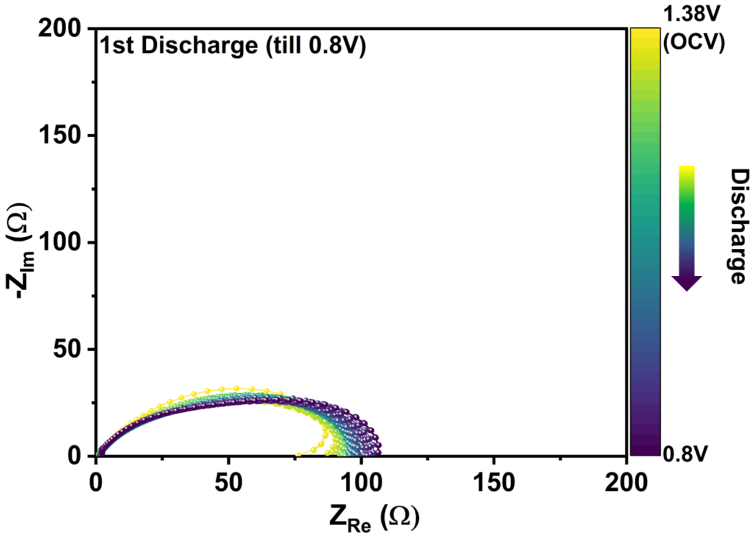

Fig. S41. *In-situ* EIS evolution of the MnO_2 electrode in a three-electrode configuration during 1st discharge, recorded every 15 mins at a current of 50 mA g^{-1}. The cell was rested for 3 h prior to impedance measurement at each point.

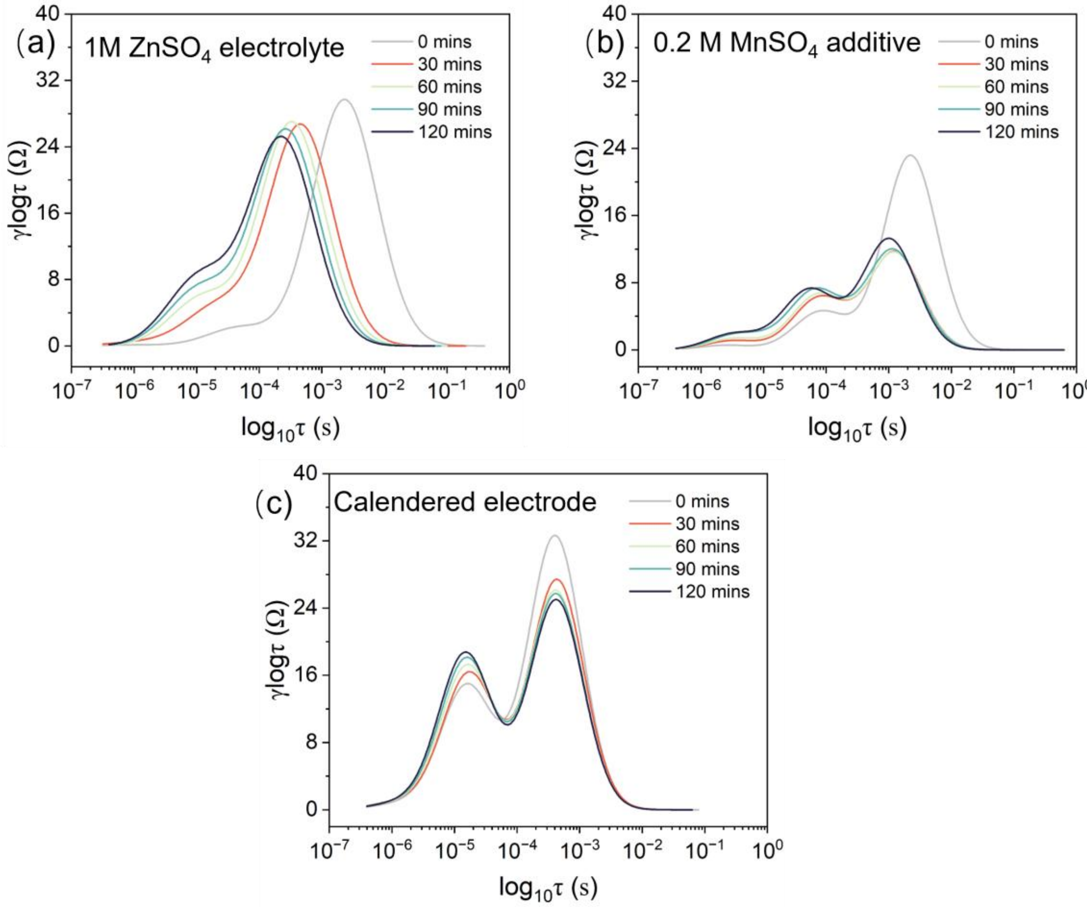

Fig. S42. SOC-resolved EIS–DRT data collected during MnO_2 discharge with (a) 1M $ZnSO_4$ electrolyte, (b) 1M $ZnSO_4$ electrolyte containing 0.2 M $MnSO_4$ additive and (c) calendered electrode and 1M $ZnSO_4$. Adding $MnSO_4$ selectively suppresses the mid-frequency P2 feature while leaving the high-frequency P1 largely unchanged. Because spectra are acquired after rest at each SOC, the peaks reflect small-signal interfacial kinetics around the open-circuit potential. The lower P2 amplitude with Mn^{2+} arises from a higher exchange current density of the MnO_2/Mn^{2+} interfacial redox, which reduces the charge-transfer resistance of the dissolution–deposition step; this should not be interpreted as accelerated net dissolution during galvanostatic discharge. Calendering increases both P1 and P2, consistent with reduced porosity and lower interfacial accessibility amplifying surface- and charge-transfer-related polarization. Taken together, these trends support assigning P1 to a proton-driven surface process and P2 to dissolution–deposition coupled charge transfer.

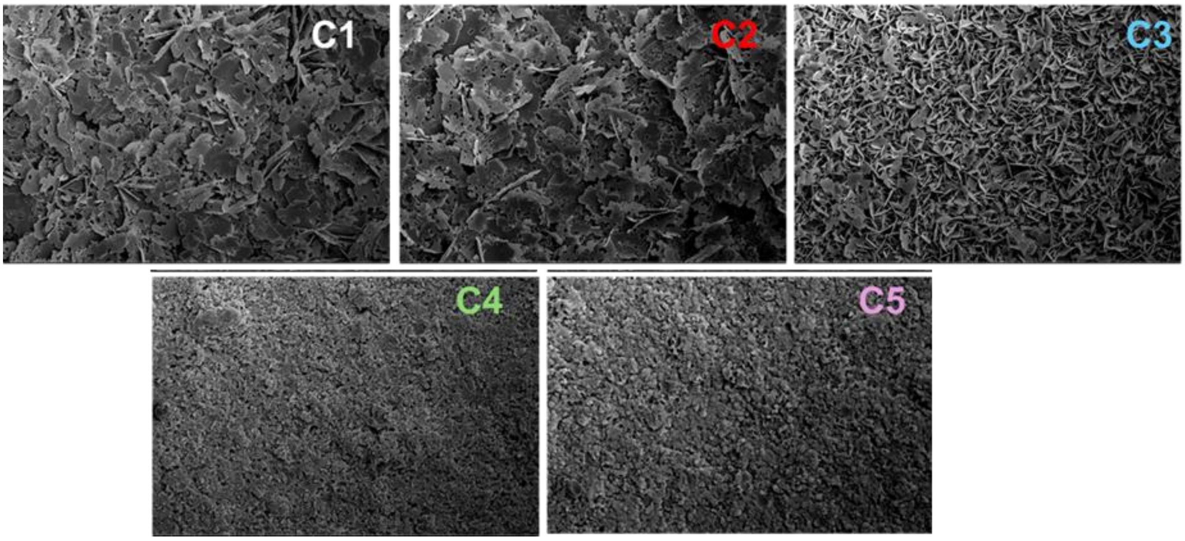

Fig. S43.The SEM images of α-MnO_2 at different charged states without acid wash.

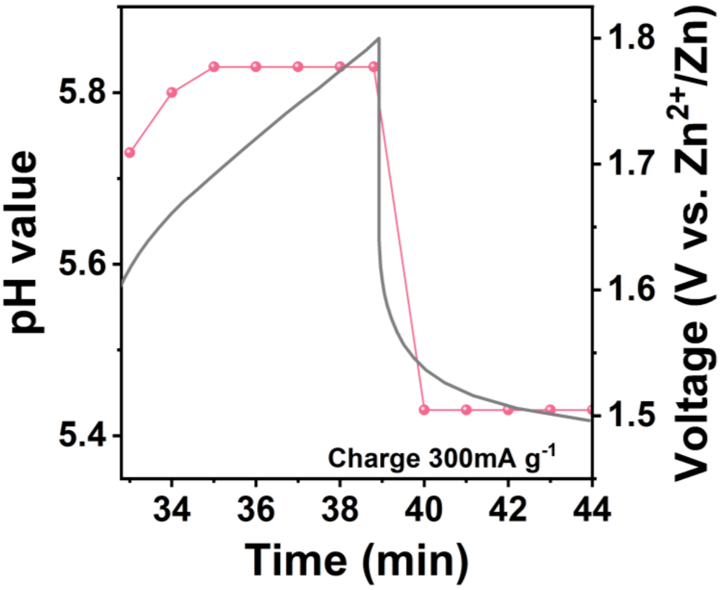

Fig. S44. The *in-situ* pH testing during charging of the Zn||MnO_2 cell at 300 mA g^{-1}. Notably, the charge capacity recorded under *in situ* pH monitoring is markedly lower than the corresponding discharge capacity, a discrepancy arising from the relatively high electrolyte volume required for reliable pH measurement, which dilutes Mn^{2+} and lengthens diffusion paths, thereby limiting redeposition efficiency.

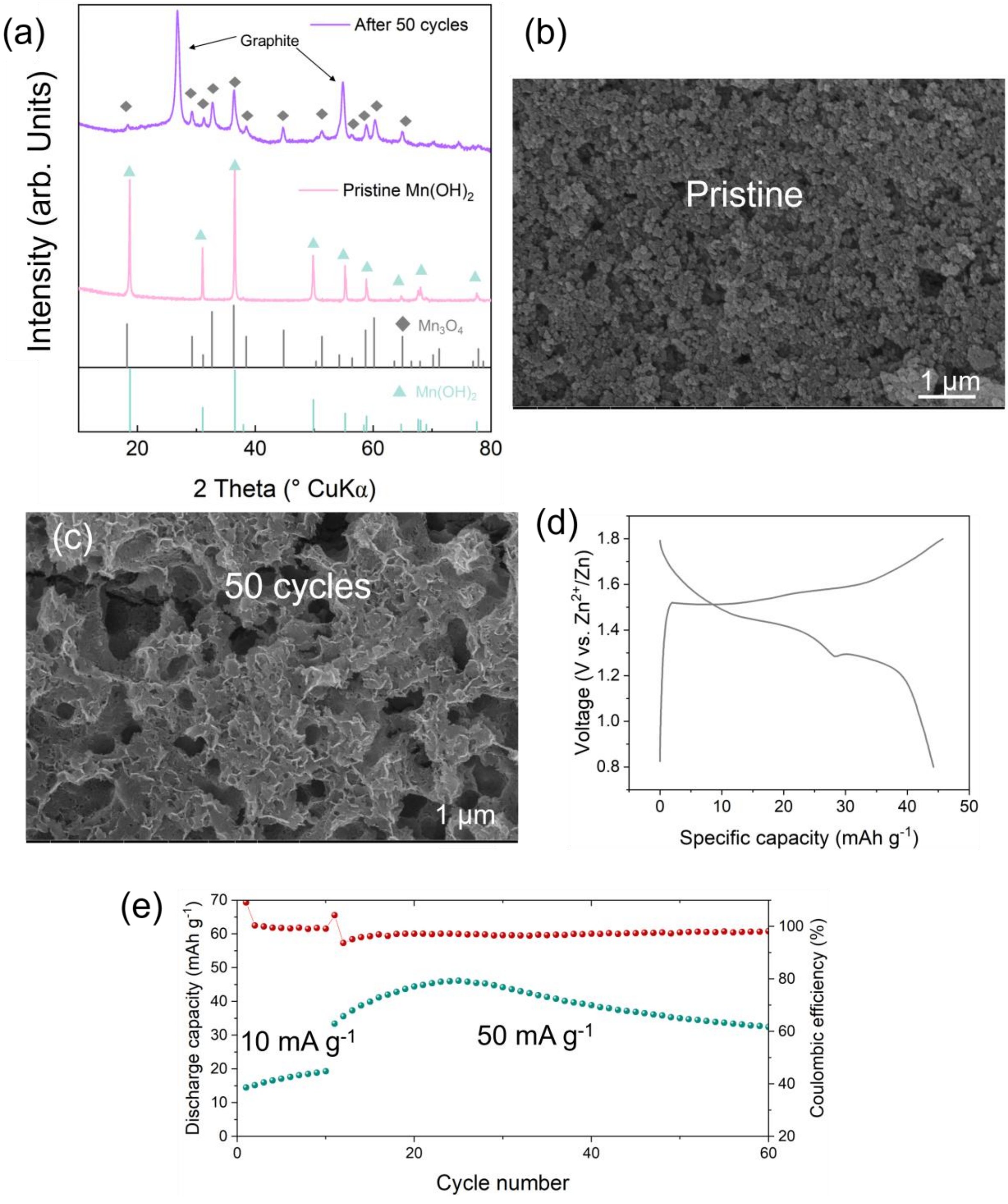

Fig. S45. (a) The XRD pattern and (b, c) SEM images for the synthesized $Mn(OH)_2$ and the corresponding phase transformation after cycling. (d) the corresponding voltage profile after 50 cycles and (e) the long-term cycling performance for the synthesized $Mn(OH)_2$.

According to the manganese Pourbaix diagram,[2] higher pH (near neutral) at the interface (Fig. S44) can promote the formation of $Mn(OH)_2$ as a metastable intermediate through the reaction of Mn^{2+} with OH^{-1}. Upon charging to a higher potential, this intermediate can undergo oxidation and deposit as Mn_3O_4 at the electrode surface. To substantiate this mechanism experimentally, we synthesized highly crystalline $Mn(OH)_2$, as shown in **Fig. S45a**, which

displays a granular morphology (**Fig. S45b**), and assembled it into a full cell paired with a zinc anode and 1M $ZnSO_4$ electrolyte. As anticipated, the cell exhibits limited initial capacity of 14 mAh g^{-1} (**Fig. S45e**) owing to the poor intrinsic conductivity of $Mn(OH)_2$.[3] Nevertheless, a progressive increase in capacity is observed over successive cycles, reaching a relatively stable performance after approximately 50 cycles, along with a similar voltage profile to that of the Zn||MnO_2 cell (**Fig. S45d**). *Ex-situ* XRD analysis of the cycled electrode confirmed a complete phase transition from $Mn(OH)_2$ to Mn_3O_4 (**Fig. S45a**). Interestingly, the charged product in the $Mn(OH)_2$||Zn cell forms as highly crystalline Mn_3O_4, in contrast to the disordered MnO_x deposited in the Zn||MnO_2 cell. This difference reflects the lower barrier for solid-state oxidation when starting from a pre-formed Mn–O(H) framework. Concurrently, morphology evolves from discrete particles to aggregated nanosheets (**Fig. S45c**), closely resembling the typical morphology of the deposition morphology of MnO_2 after charging. Such transformation indicates a likely mechanistic pathway, where the local interfacial pH becomes relatively high and hence fosters local Mn^{2+} to transiently form a metastable $Mn(OH)_2$, which is electrochemically converted to mixed-valent MnO_x.

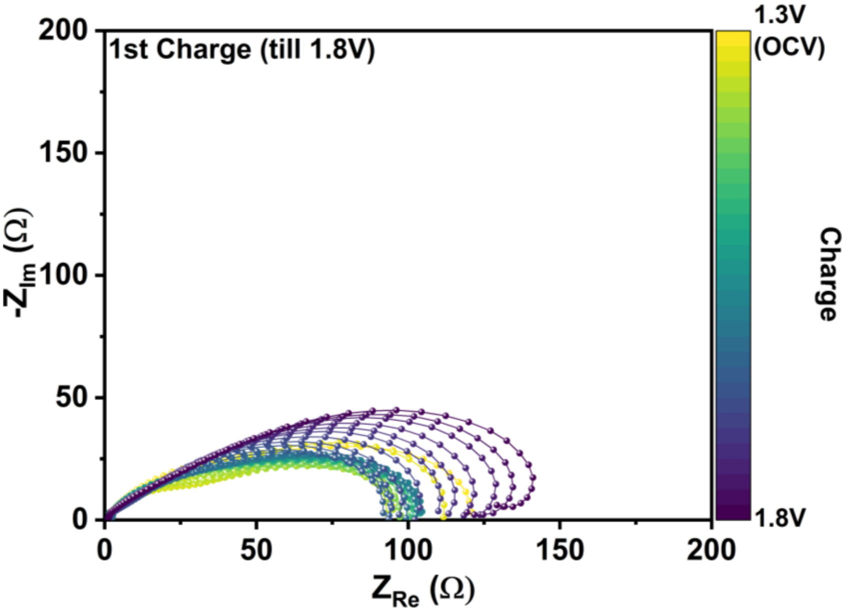

Fig.S46. *In-situ* EIS evolution of the MnO_2 electrode in a three-electrode configuration during 1st charge, recorded every 15 mins at a current of 50 mA g^{-1}. The cell was rested for 3 h prior to impedance measurement at each point.

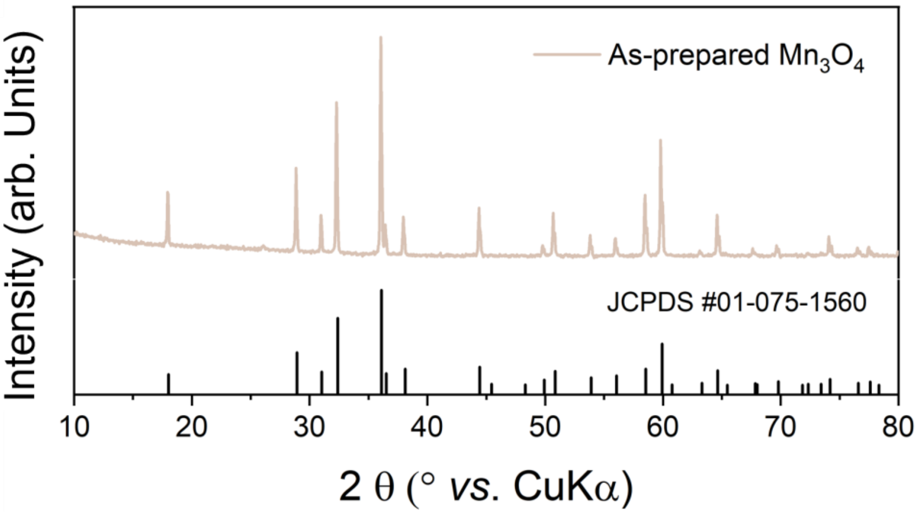

Fig.S47. The XRD pattern of the as-synthesized Mn_3O_4.

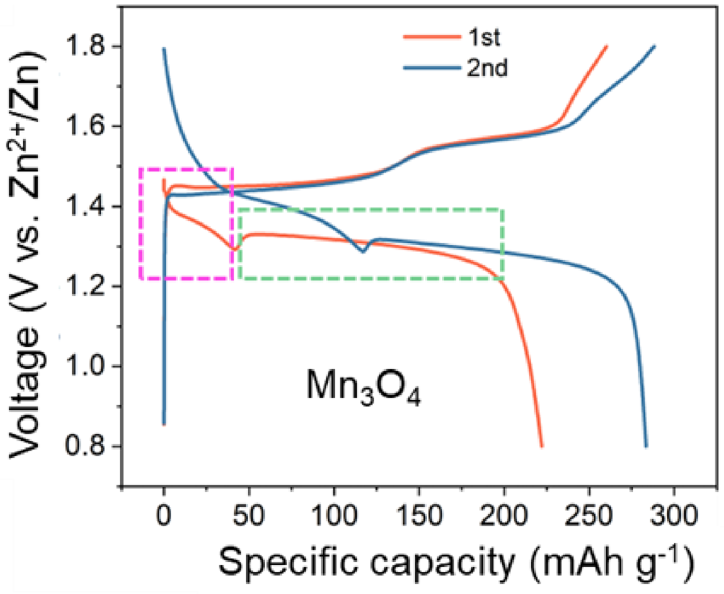

Fig. S48. The GCD profile of Zn||Mn_3O_4 cell for the first two cycles while cycling at 100 mA g^{-1} in the range of 0.8- 1.8 V vs. Zn^{2+}/Zn. These data are presented to benchmark electrochemical signatures of Mn_3O_4 against those observed for the deposited MnO_x. This comparison does not imply that the deposited phase is crystalline Mn_3O_4.

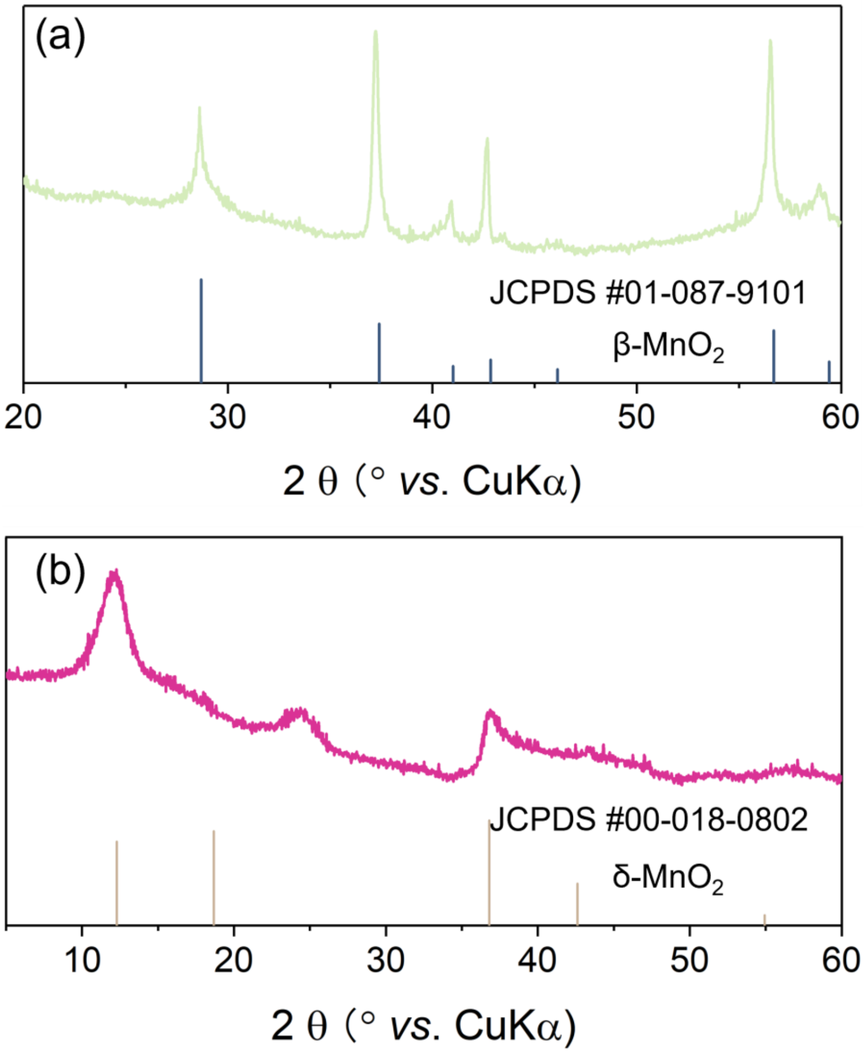

Fig. S49. The XRD pattern of the as-prepared (a) β-MnO_2 and (b) δ-MnO_2.

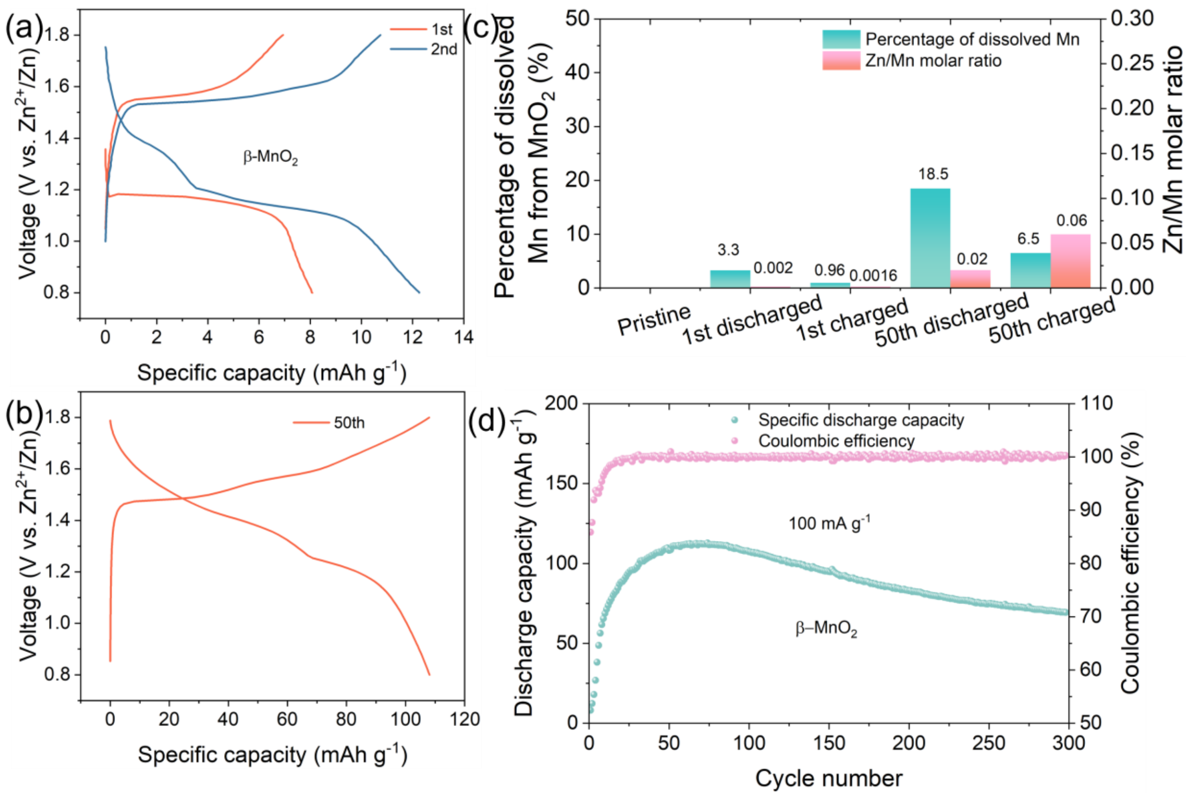

Fig. S50. The GCD profile of β-MnO_2 cycling at 100 mA g^{-1} for (a) initial two cycles and (b) 50th cycle. (c) The ICP-OES results for the electrolyte and β-MnO_2 electrode after 1st and 50th cycle. (d)The long-term stability of the Zn||β-MnO_2 cell at 100 mA g^{-1}.

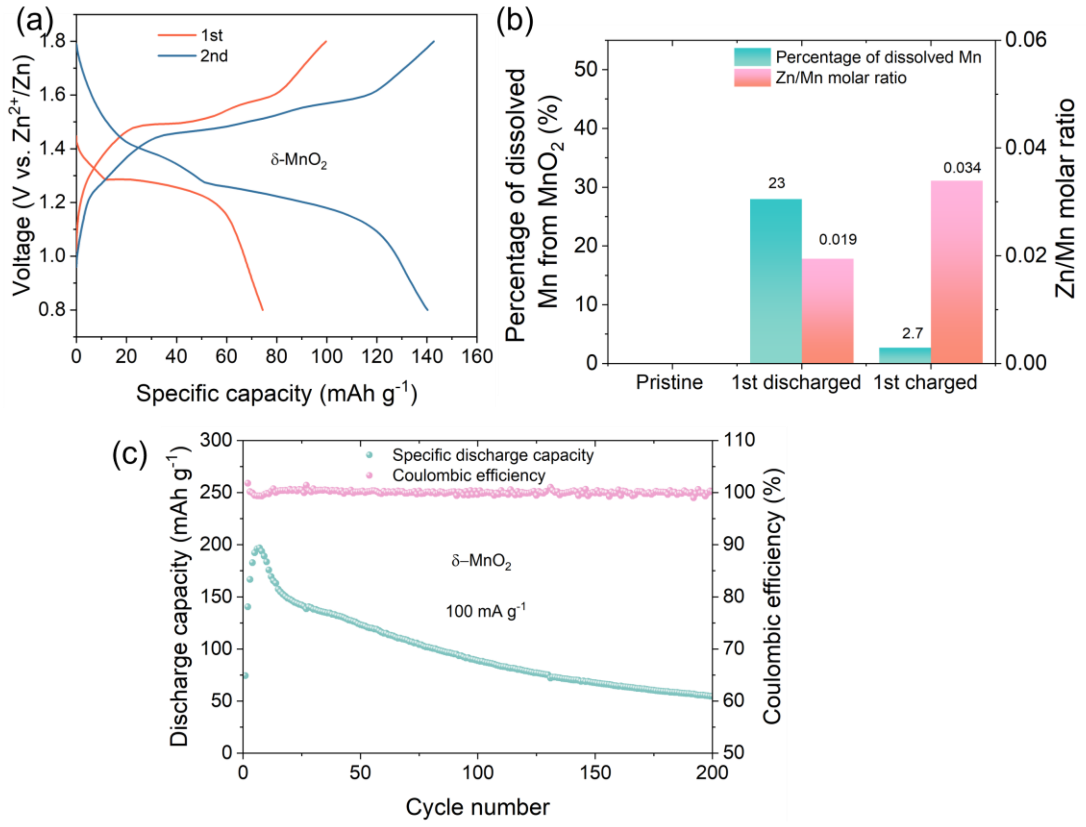

Fig. S51. (a) The GCD profile of β-MnO_2 cycling at 100 mA g^{-1} for the initial two cycles. (b) The ICP-OES results for the electrolyte and β-MnO_2 electrode after 1st and 50th cycle. (c) The long-term stability of the Zn||β-MnO_2 cell at 100 mA g^{-1}.

To investigate the universality of the dissolution-deposition chemistry for manganese-based oxide cathodes, other candidates, β-MnO_2 and δ-MnO_2, featuring tunnel and layered structures (**Fig. S49**), respectively, were employed as cathodes and analyzed by the same methodology. As shown in **Figs. S50a** and **S51a**, both β-MnO_2 and δ-MnO_2 exhibit a similar evolution in their respective voltage profiles: transitioning from a single-plateau profile to a two-plateau profile, akin to that observed for α-MnO_2. However, the β-MnO_2 electrode exhibits a low specific capacity during the initial stage, gradually increasing to 108 mAh g^{-1} after 50 cycles (**Fig. S50b**). The growth in capacity aligns well with the ICP-OES data, indicating that β-MnO_2 undergoes subtle dissolution-deposition chemistry during the first cycle and then 18.5% Mn in electrode dissolves after 50 cycles, without Zn^{2+}-involved reaction or Zn^{2+} intercalation (**Fig. S50c**). Likewise, the dissolution-deposition behavior for δ-MnO_2 is also confirmed by ICP-OES results (**Fig. S51b**), highlighting that dissolution-deposition chemistry is applicable to other MnO_2 polymorphs irrespective of their crystal structure differences. Not surprisingly, both β-MnO_2 and δ-MnO_2 electrode experience gradual capacity decay for long-term cycling (**Fig.S50d** and **Fig.S51c**).

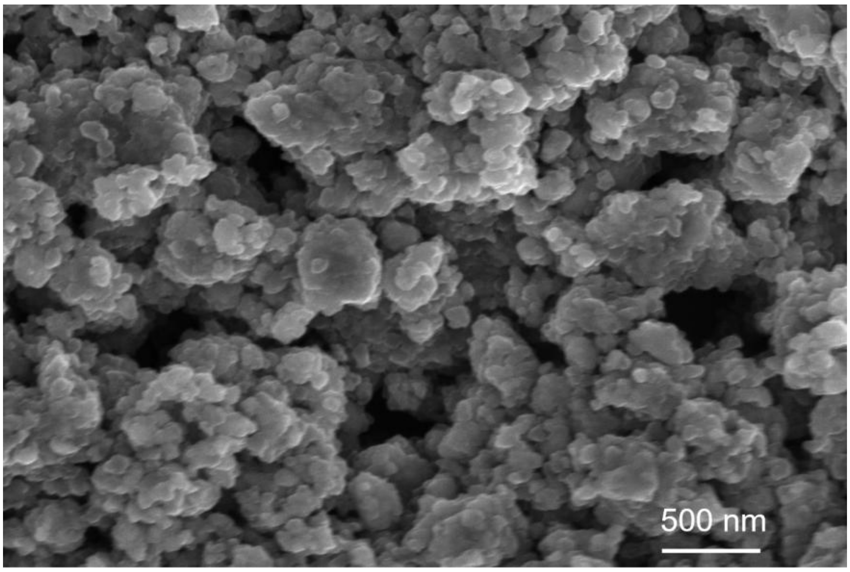

Fig. S52. The SEM image for BM-α-MnO_2.

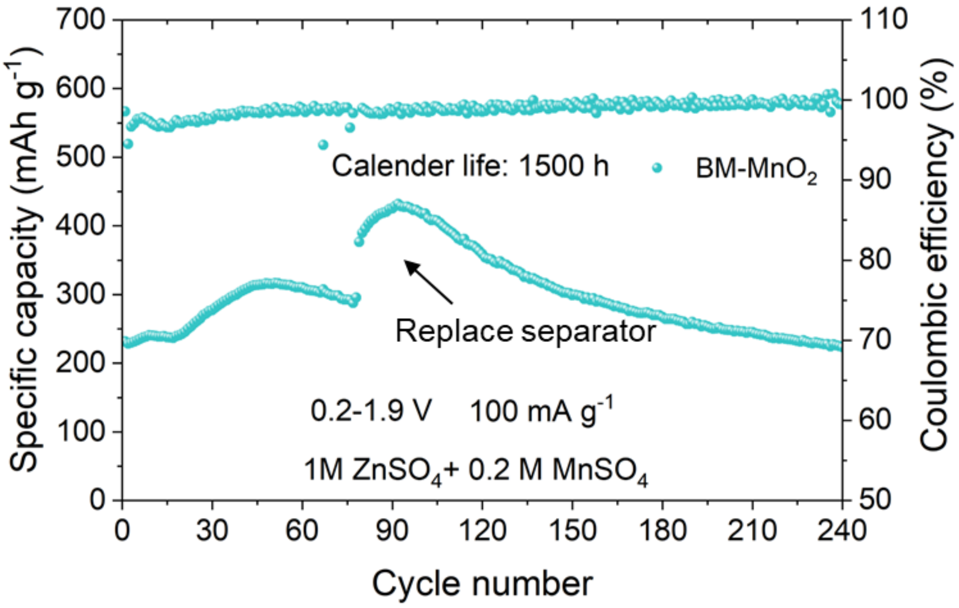

Fig. S53. The superior stability of Zn||BM-MnO_2 when employing 1M $ZnSO_4$-0.2 $MnSO_4$ electrolyte at 100 mA g^{-1}.

Leveraging the pre-incorporation of Mn^{2+} ions and the availability of more reactive sites in BM-MnO_2, the material exhibits a gradual increase in capacity, reaching 315 mAh g^{-1} after 50 cycles. Moreover, following the replacement of the separator with a fresh Mn-containing electrolyte at the 80th cycle, the specific capacity bounds to 376 mAh g^{-1} and further grows to 431 mAh g^{-1} at the 92nd cycle. This behavior highlights that the pre-added Mn^{2+} acts as a reservoir, contributing additional capacity from the electrolyte rather than suppressing dissolution. Benefiting this, the BM-MnO_2 can exhibit an ultra-long cyclability for 240 cycles, corresponding to over 1500 h.

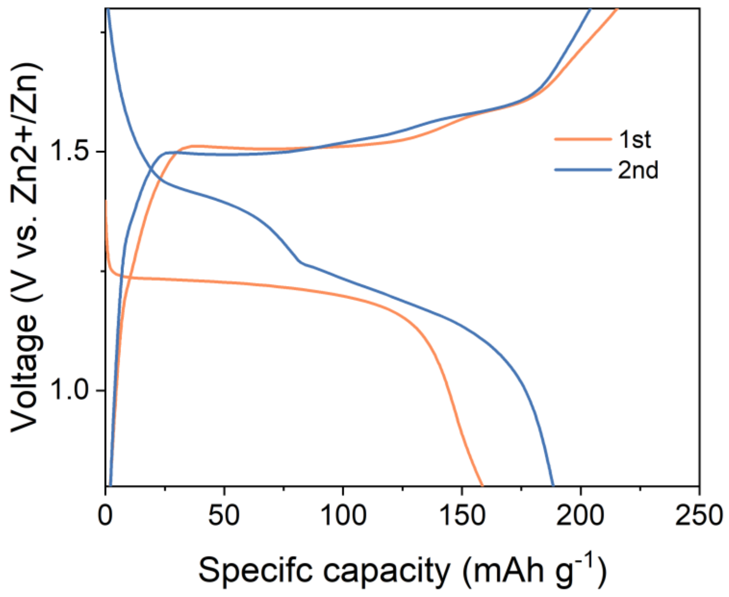

Fig. S54. The GCD profile for Zn||BM-α-MnO_2 cell during the first two cycles.

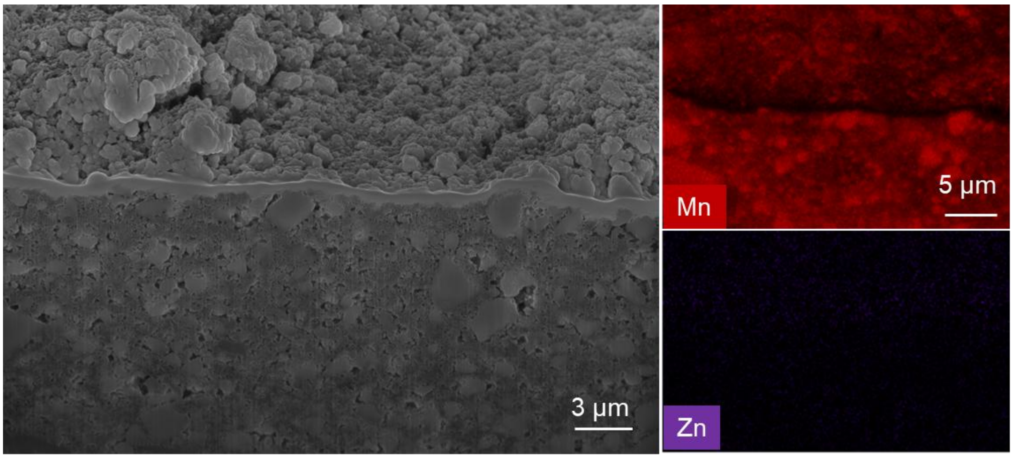

Fig. S55. The FIB-SEM and corresponding EDS image for 10%-Gra- BM-α-MnO_2 electrode before cycling.

Supplementary References

1. Garvie, L. A. J. & Craven, A. J. High-resolution parallel electron energy-loss spectroscopy of Mn L2,3-edges in inorganic manganese compounds. *Phys Chem Minerals* **1994**, *21*, 191–206.
2. Crapnell, R. D. & Banks, C. E. Electroanalytical overview: The determination of manganese. *Sensors and Actuators Reports* **2022**, *4*, 100110.
3. Ham, K., Lee, J., Lee, K. & Lee, J. Boosting the oxygen evolution reaction performance of wrinkled $Mn(OH)_2$ via conductive activation with a carbon binder. *J. Energy Chemistry* **2022**, *71*, 580–587.
