# Supplementary material for: Decoding MnO2 redox chemistry from mechanistic ambiguity to design principles for aqueous Zn-ion batteries
Source: Nat Commun. 2026 Jun 11;17:7433. doi: 10.1038/s41467-026-74350-z (PMC13408579; doi:10.1038/s41467-026-74350-z)
Supplement: Supplementary file 2 — Description of Additional Supplementary Files [file 41467_2026_74350_MOESM2_ESM.pdf]

Description of Additional Supplementary Files

File Name: Supplementary Data 1
Description: initial structure of MnO_2 (110) surface slab

File Name: Supplementary Data 2
Description: final structure of MnO_2 (110) surface slab

File Name: Supplementary Data 3
Description: initial structure of H+ on MnO_2 (110) surface slab

File Name: Supplementary Data 4
Description: final structure of H+ on MnO_2 (110) surface slab

File Name: Supplementary Data 5
Description: initial structure of H+ in MnO_2 (110) surface slab

File Name: Supplementary Data 6
Description: final structure of H+ in MnO_2 (110) surface slab

File Name: Supplementary Data 7
Description: initial structure of $H_{0.25}MnO_2$

File Name: Supplementary Data 8
Description: final structure of $H_{0.25}MnO_2$

File Name: Supplementary Data 9
Description: initial structure of $H_{0.5}MnO_2$

File Name: Supplementary Data 10
Description: final structure of $H_{0.5}MnO_2$

File Name: Supplementary Data 11
Description: initial structure of $H_{0.25}MnO_2$ AIMD

File Name: Supplementary Data 12
Description: final structure of $H_{0.25}MnO_2$ AIMD

File Name: Supplementary Data 13
Description: initial structure of $H_{0.5}MnO_2$ AIMD

File Name: Supplementary Data 14
Description: final structure of $H_{0.5}MnO_2$ AIMD
