# Supplementary material for: Decoding MnO2 redox chemistry from mechanistic ambiguity to design principles for aqueous Zn-ion batteries
Source: Nat Commun. 2026 Jun 11;17:7433. doi: 10.1038/s41467-026-74350-z (PMC13408579; doi:10.1038/s41467-026-74350-z)
Supplement: Supplementary file 4 — Transparent Peer Review file [file 41467_2026_74350_MOESM4_ESM.pdf]

nature portfolio

Peer Review File

Decoding MnO_2 Redox Chemistry From Mechanistic Ambiguity to Design Principles for Aqueous Zn-ion Batteries

Corresponding Author: Professor Dipan Kundu

This file contains all reviewer reports in order by version, followed by all author rebuttals in order by version.

Version 0:

Reviewer comments:

Reviewer #1

(Remarks to the Author)
The manuscript presents a comprehensive and carefully executed mechanistic study of MnO_2 cathodes in aqueous Zn-ion batteries. By combining operando synchrotron XRD, quantitative ICP-OES analysis, depth-resolved X-ray absorption spectroscopy, electron microscopy, electrochemical analysis, and theoretical calculations, the authors convincingly clarify the long-standing controversy regarding the charge storage mechanism of MnO_2.
Overall, I find the manuscript suitable for publication in Nature Communications. Below, I list several comments that are intended to improve the clarity and impact of the manuscript.
1. The separator replacement experiment is well designed and provides important evidence for distinguishing dissolution–deposition from non-dissolution contributions. In the main text, it is stated that the recovered capacity reaches ~45 mAh g^{-1} in the second cycle and stabilizes at ~70 mAh g^{-1} after five cycles (corresponding to ~40% of the discharge capacity). It would be helpful if the authors could clarify how this percentage is defined, i.e., whether it is normalized to the discharge capacity of the same cycle or to a reference cycle.
2. Several key conclusions rely on the average Mn oxidation states obtained from Mn L-edge linear combination fitting. As these values are central to the mechanistic discussion, it would strengthen the presentation if the authors could provide additional fitting details in the manuscript or experimental sections.
3. The manuscript uses the term “electrochemically inactive (dead) Mn^{2+}” to describe Mn species accumulated in the electrolyte during cycling. The authors need to further clarify why these Mn^{2+} ions do not redeposit onto the electrode, particularly given that a higher Mn^{2+} concentration in the electrolyte would, in principle, favor the deposition reaction.
4. In Figure S40, the ZSH-related peaks are not fully consistent across different samples. For example, the characteristic ZSH peak appears at approximately ~12º in D4, whereas in other samples it is located at around ~8º.
5. Figure S44 presents an interesting theoretical analysis. Based on this framework, the manuscript implies that $Mn(OH)_2$ preferentially transforms into crystalline Mn_3O_4 rather than an amorphous MnO_x phase. Regarding this discrepancy, the authors need to clarify why Mn_3O_4 is thermodynamically or kinetically favored under these conditions.

Reviewer #2

(Remarks to the Author)
The manuscript reports a large set of characterization data to describe the complex mechanism of Zn/MnO_2 batteries. Data includes synchrotron XRD and XAS, as well as extensive TEM-EELS, operando pH measurements, and ICP-OES plus DFT calculations and is valuable for insights on the composition of the electrodes and electrolytes. Although the discussion and conclusions are generally updated and well argumented, the scope appears eccessively broad, as most aspects of the multifaceted mechanism are addressed: the mechanism for first discharge, the limited first discharge capacity, the formation of the buffering ZSH precipitate, surface/interface alteration of the active material, the morphology and composition of the electrodeposited Mn oxide and its evolution along the first two full cycles as well as after tens of cycles.
This dispersion results in conclusions which are often anticipated by existing literature, without that established and novel points are duly differentiated.
While the data are valuable and its interpretation is usually convincing, a more compact structure focused on most relevant findings would certainly make the paper more valuable.
The following points should be also considered:
1) The comparison of literature trends on the main mechanism proposals in literature (scheme 1) is interesting. It shows a

timeline, mentions the first suggestion of the main concepts, and the number of papers supporting the respective models. A graphics or table with the distribution of the publications on a yearly basis would better illustrathe the evolution of their acceptance.
2) Intercalation/conversion as a main mechanism has already been conclusively ruled out in favor of the dissolution/precipitation mechanism by several publications (e.g. DOI: 10.1002/cssc.201600702; 10.1016/j.mtener.2020.100396; 10.1016/j.cej.2022.137205; 10.1039/D0EE02168G; 10.1002/anie.202318444). The mechanism is also found with Mn_2O_3 (10.1016/j.jpowsour.2022.231023). Although some observations are interesting, their description could be more summarized in the main text.
3) The discrepancy between experimental capacity and that expected for a dissolution mechanism has been recently studied in detail (DOI: 10.1039/D5EE03588K) ruling out the surface passivation by Zn compounds. These conclusions should be considerd and compared with the observations provided in the manuscript, which are otherwise largely confirming the other aspects reported earlier. Consequently, this section of the main text may also be shortened.
4) In line 354 ICP-OES data indicates steady increase of Mn^{2+} in solution. The discussion is not clear. If deposited MnO_2 only partially dissolve why Mn^{2+} increases in electrolyte? why Mn^{2+} increases solution should become electrochemically inactive?
5) Likewise, an excessive Mn^{2+} is found at early stages of discharge, at amounts that cannot be explained by an electrochemical process (line 368). The provided interpretation based on intercalation seems confusing.
6) line 468 mentions "proton de-intercalation from MnO_2". The chemical formula should contain H, or residual presence of protons mentioned.
7) sentence at line 486 "...with first evaluated with a moderate-low active loading..." should be checked

Reviewer #3

(Remarks to the Author)
1. The application of EIS–DRT analysis to deconvolute kinetic contributions is a strong aspect of this work. In the in situ EIS measurements, the manuscript indicates that the cell was rested for 3 h prior to impedance acquisition at each selected point. Although such a rest period may help the system approach a quasi-equilibrium condition, it also raises questions regarding how closely the measured impedance reflects the actual electrochemical state reached during galvanostatic discharge.
After a 3 h rest, the equilibrium potential may deviate significantly from the instantaneous discharge potential, particularly in a system involving Mn dissolution and interfacial re-equilibration. Providing the full E–t evolution during the discharge–rest–EIS sequence would help clarify how representative the impedance measurements are of the preceding electrochemical process. In addition, during such extended rest periods, self-discharge and spontaneous Mn dissolution are difficult to avoid and may contribute to the observed impedance response. A brief discussion of how these effects are considered, or how the results might differ if impedance were acquired immediately after a fixed discharge time or under potentiostatic conditions, would improve confidence in the interpretation of the EIS data and the subsequent DRT analysis.
At the same time, the attribution of specific DRT (P1 and P2 area) features to proton-related surface processes or to dissolution–redeposition pathways is inherently non-unique, as similar time constants may arise from changes in interphase resistance, porous transport, or surface morphology during cycling.
Framing the DRT results with a clearer discussion of these possible overlaps, or illustrating how the observed features respond to controlled variations in electrochemical or material parameters (such as Mn^{2+} concentration, electrode thickness, or electrolyte composition), would help readers better appreciate the physical meaning and limitations of the DRT-based assignments.
2. Throughout the manuscript, the redeposited phase is described as "Mn_3O_4-like," mainly based on average Mn valence and electrochemical characteristics. While this interpretation is reasonable, it is worth noting that Mn redeposition in aqueous systems often leads to structurally disordered, hydrated, or mixed-valence MnO_x phases, especially after extended cycling.
Placing this discussion in the context of recent studies that combine long-term structural analysis with techniques such as solid-state NMR would help clarify whether a Mn_3O_4-like description is uniquely supported in the present case, or whether a more general description of mixed-valent MnO_x would be more appropriate (for example, see recent analyses reported in 10.1021/acsenergylett.4c02559).
3. The detection of Mn^{2+} at early stages of discharge is an intriguing observation that supports the proposed reaction pathway. However, Mn dissolution can also occur during rest or originate from residual surface species, making it important to clearly distinguish electrochemically driven dissolution from spontaneous chemical processes. For example, add D0 point in Fig. 4a.
Clarifying whether similar Mn^{2+} release is observed under open-circuit conditions, or briefly discussing relevant control experiments, would help reinforce the interpretation that the early-stage dissolution observed here is intrinsically linked to the electrochemical process.
4. The manuscript emphasizes proton involvement and surface proton priming as key elements of the reaction mechanism. This discussion would benefit from being more explicitly situated within the broader context of recent work on proton insertion and H_xMnO_2 formation in α-MnO_2-based cathodes. In particular, recent studies employing techniques such as EQCM-D in combination with solid-state NMR have provided direct insight into proton insertion behavior and the stabilization of H_xMnO_2 phases. Discussing how the present findings align with, extend, or differ from these observations (e.g., 10.1002/smll.202404583) would further strengthen the mechanistic framing and highlight the contribution of the current work.

Version 1:

Reviewer comments:

Reviewer #1

(Remarks to the Author)
I thank the authors for their detailed and careful revisions. They have adequately responded to all my comments, and the manuscript has been improved accordingly. The data presented are now clear and support the conclusions. Therefore, I recommend the manuscript for publication.

Reviewer #2

(Remarks to the Author)
The authors maintained the extensive structure of their manuscript, although they did a significant effort to take specific comments into consideration. However, some points are still not satisfactorily addressed.
3) In spite of the fact that it is now recognized at page 8 that also other passivating mechanisms can be responsible for the MnO2 passivation, ZSH is still repeatedly indicated as the main responsible for kinetic and capacity limitation (lines 207, 367, 411, 446, 487, 548, 568). It is certainly difficult to discriminate which specific passivation effect is responsible for the hindered dissolution, but direct evidence for the ZHS alone does not seem strong enough. A more generic “passivation” term seems more correct.
4) it is unrealistic that during several cycles the contribution of parent MnO2 to capacity continues to be relevant. It remains unclear how Mn2+ in solution can become electrochemically inactive. Its accumulation must be rather related to increased difficulty of MnOx to form. The accumulation of insulating components on the electrode might be one possible cause.
5) dissolution of Mn(IV) to Mn2+ needs electrons, if the process is not faradaic, what chemical species will provide them?
The reported observations are certainly intriguing, even if some of the explanations can remain partial and open.

Reviewer #3

(Remarks to the Author)
The authors have addressed all the comments. I suggest it be accepted for publication.

Open Access This Peer Review File is licensed under a Creative Commons Attribution 4.0 International License, which permits use, sharing, adaptation, distribution and reproduction in any medium or format, as long as you give appropriate credit to the original author(s) and the source, provide a link to the Creative Commons license, and indicate if changes were made.
In cases where reviewers are anonymous, credit should be given to 'Anonymous Referee' and the source.
The images or other third party material in this Peer Review File are included in the article’s Creative Commons license, unless indicated otherwise in a credit line to the material. If material is not included in the article’s Creative Commons license and your intended use is not permitted by statutory regulation or exceeds the permitted use, you will need to obtain permission directly from the copyright holder.
To view a copy of this license, visit https://creativecommons.org/licenses/by/4.0/

Response letter to reviewers

We are grateful to the reviewers for their precious time in assessing our manuscript and providing critical assessment. Here, we are providing a detailed response to each comment. Reviewers' comments and suggestions are *italicized*, and our answers are in blue for clarity. Any changes in the manuscript are highlighted in yellow.

Reviewer #1 (Remarks to the Author): *The manuscript presents a comprehensive and carefully executed mechanistic study of MnO2 cathodes in aqueous Zn-ion batteries. By combining operando synchrotron XRD, quantitative ICP-OES analysis, depth-resolved X-ray absorption spectroscopy, electron microscopy, electrochemical analysis, and theoretical calculations, the authors convincingly clarify the long-standing controversy regarding the charge storage mechanism of MnO2. Overall, I find the manuscript suitable for publication in Nature Communications. Below, I list several comments that are intended to improve the clarity and impact of the manuscript.*

We thank the reviewer for the positive evaluation of our work and for the constructive comments. A point-by-point response to the reviewer's comments is provided below.

1. The separator replacement experiment is well designed and provides important evidence for distinguishing dissolution–deposition from non-dissolution contributions. In the main text, it is stated that the recovered capacity reaches ~45 mAh g-1 in the second cycle and stabilizes at ~70 mAh g-1 after five cycles (corresponding to ~40% of the discharge capacity). It would be helpful if the authors could clarify how this percentage is defined, i.e., whether it is normalized to the discharge capacity of the same cycle or to a reference cycle.

We appreciate the reviewer's observation. The reported percentage is defined as the recovered *charge* capacity normalized to the *first discharge* capacity, which is used as the reference. To avoid ambiguity, we have now stated this explicitly in the manuscript.

"Unlike the first cycle, where the charge capacity collapses post-replacement, subsequent cycles show notable recovery: 45 mAh g^{-1} after the second cycle and stabilization around 70 mAh g^{-1} after the fifth cycle (~40% relative to the first discharge capacity)."

2. Several key conclusions rely on the average Mn oxidation states obtained from Mn L-edge linear combination fitting. As these values are central to the mechanistic discussion, it would strengthen the presentation if the authors could provide additional fitting details in the manuscript or experimental sections.

We thank the reviewer for highlighting this point. We have now expanded the 'Methods' section to describe the LCF procedure more transparently. The added text specifies the reference spectra employed (Mn(II), Mn(III), mixed-valent Mn, and Mn(IV)), the identical data-collection modes used for references and samples, and the way the average oxidation state is computed.

"The LCF was performed over the Mn L_3 and L_2 edges using reference spectra for Mn(II), Mn(III), mixed-valent Mn, and Mn(IV), collected and processed under identical conditions.

The average Mn oxidation state was obtained as the coefficient-weighted sum of the nominal oxidation states."

3. The manuscript uses the term "electrochemically inactive (dead) Mn2+" to describe Mn species accumulated in the electrolyte during cycling. The authors need to further clarify why these Mn2+ ions do not redeposit onto the electrode, particularly given that a higher Mn2+ concentration in the electrolyte would, in principle, favor the deposition reaction.

We thank the reviewer for this insightful comment. While higher Mn^{2+} concentration in the electrolyte can, in principle, promote deposition kinetics, our data show that two processes prevent complete reversibility.

First, operando XRD demonstrates (Fig. 2a) that insulating byproduct ZSH does not fully dissolve during charging. Second, TEM (Figs. 2h and S26) shows that a fraction of the deposited MnO_x nanosheets also remains after discharge. These residual phases progressively occupy active interfacial sites, hindering accessibility and limiting the ability of dissolved Mn^{2+} to redeposit. Meanwhile, continued dissolution of the parent α-MnO_2 contributes additional Mn^{2+} to the electrolyte, consistent with the steady rise measured by ICP-OES. We have clarified this point and revised the manuscript accordingly, as follows:

"Collectively, this behavior arises from ongoing dissolution of the parent MnO_2 alongside progressive surface blockage by residual MnO_x and ZSH, which restrict accessible sites and kinetically hinder Mn^{2+} redeposition"

4. In Figure S40, the ZSH-related peaks are not fully consistent across different samples. For example, the characteristic ZSH peak appears at approximately ~12° in D4, whereas in other samples it is located at around ~8°.

We appreciate the reviewer's careful observation. The low-angle ZSH peak typically appears near 8° (2θ, Cu Kα) for highly hydrated $Zn_4(OH)_6SO_4{\cdot}5H_2O$ formed during discharge. However, partial dehydration can occur during sample drying, reducing the interlayer spacing and shifting the reflection to higher angles (DOI: 10.1039/c3dt51638e). This explains the ~12° peak in the D3 sample. The underlying conclusions are unaffected, as the MnO_2 reflections remain stable at the drying temperature (60 °C).

5. Figure S44 presents an interesting theoretical analysis. Based on this framework, the manuscript implies that Mn(OH)2 preferentially transforms into crystalline Mn3O4 rather than an amorphous MnOx phase. Regarding this discrepancy, the authors need to clarify why Mn3O4 is thermodynamically or kinetically favored under these conditions.

We appreciate the reviewer's insightful comment regarding the distinct deposition behaviour observed when using MnO_2 versus $Mn(OH)_2$ as electrodes.

As shown in the Pourbaix diagram (Fig. S45a), the thermodynamically favored manganese phases depend strongly on the local pH within the fixed cycling window. According to the pH evolution in Fig. S44, the interfacial pH rises to ~5.8 during charging and is likely higher at the electrode surface. This shifts the system towards the stability regime of $Mn(OH)_2$ and Mn_3O_4.

Based on this, we propose an $Mn(OH)_2$-mediated pathway, $Mn^{2+} \rightarrow Mn(OH)_2 \rightarrow MnO_x$, where $Mn(OH)_2$ precipitates as a metastable intermediate from Mn^{2+} at elevated local pH.

The key distinction between the two electrode systems lies in how this intermediate is generated and evolves. For the MnO_2 electrode, redeposition occurs via solution-phase nucleation under non-equilibrium conditions - local pH spikes, ZSH coverage, and rapid nucleation kinetics - all of which hinder long-range ordering, leading to disordered (near amorphous) MnO_x.

In contrast, when $Mn(OH)_2$ itself is used as the electrode, the system starts from a pre-existing Mn–O(H) solid framework. This lowers the kinetic barrier for solid-state oxidation to Mn_3O_4, allowing structural ordering during conversion. This mechanistic distinction is now clarified in the SI as follows:

"Interestingly, the charged product in the $Mn(OH)_2$//Zn cell forms as highly crystalline Mn_3O_4, in contrast to the disordered MnO_x deposited in the MnO_2//Zn cell. This difference arises because $Mn(OH)_2$ provides a pre-existing Mn–O(H) framework, which lowers the kinetic barrier for oxidation and enables structural ordering during the $Mn(OH)_2 \rightarrow Mn_3O_4$ conversion."

Reviewer #2 (Remarks to the Author):

The manuscript reports a large set of characterization data to describe the complex mechanism of Zn/MnO2 batteries. Data includes synchrotron XRD and XAS, as well as extensive TEM-EELS, operando pH measurements, and ICP-OES plus DFT calculations and is valuable for insights on the composition of the electrodes and electrolytes. Although the discussion and conclusions are generally updated and well argumented, the scope appears eccessively broad, as most aspects of the multifaceted mechanism are addressed: the mechanism for first discharge, the limited first discharge capacity, the formation of the buffering ZSH precipitate, surface/interface alteration of the active material, the morphology and composition of the electrodeposited Mn oxide and its evolution along the first two full cycles as well as after tens of cycles.

This dispersion results in conclusions which are often anticipated by existing literature, without that established and novel points are duly differentiated. While the data are valuable and its interpretation is usually convincing, a more compact structure focused on most relevant findings would certainly make the paper more valuable.

We thank the reviewer for the constructive assessment and for recognising the breadth of our characterization. To address the reviewer's broader concern around dispersion of results and differentiation from prior insights, we have now streamlined the narrative by condensing several sections, tightening mechanistic arguments, and rewriting the conclusions to emphasise the most consequential findings. These revisions make the manuscript more compact and sharpen the distinction between prior knowledge and the contributions of this work.

The following points should be also considered:

1) The comparison of literature trends on the main mechanism proposals in literature (scheme 1) is interesting. It shows a timeline, mentions the first suggestion of the main concepts, and the number of papers supporting the respective models. A graphics or table with the distribution of the publications on a yearly basis would better illustrate the evolution of their acceptance.

We thank the reviewer for this helpful suggestion. Building on our literature survey, we categorized reports according to the dominant mechanism proposed and compiled the annual publication trends (**Fig. R1/ Fig. S1**). This breakdown provides a clearer view of how different mechanistic interpretations have evolved. Notably, despite accumulating evidence against Zn^{2+} insertion, a significant fraction of recent studies still rely on Zn^{2+} insertion or Zn^{2+}/H^{+} co-insertion, whereas dissolution–deposition remains under-represented. The manuscript has been updated to reflect this expanded analysis.

"Our analysis of more than 1,100 publications from the past ten years reveals that these models have not converged: Zn^{2+} insertion and co-insertion still dominate the literature even in the recent years (**Fig. S1**), while only a small fraction acknowledge dissolution–deposition, despite growing experimental evidence."

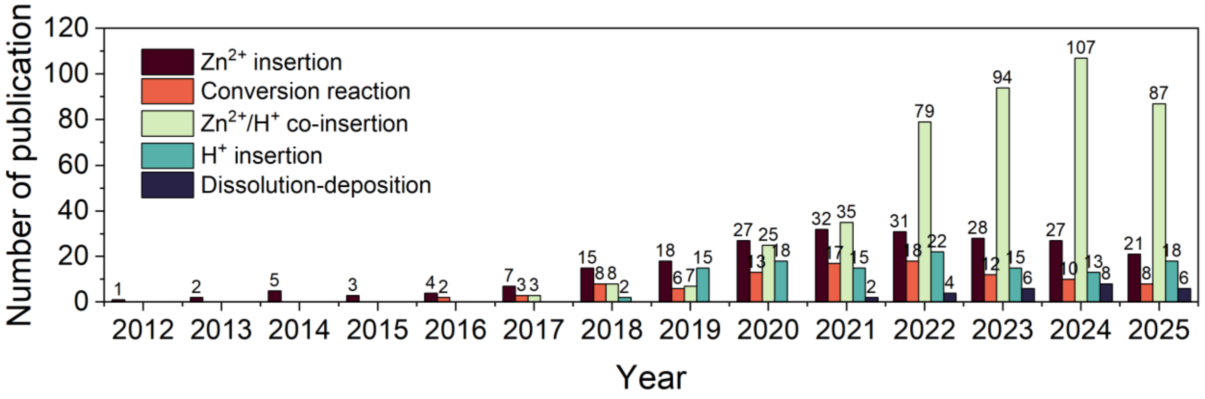

Fig. R1 (Fig. S1). Annual number of publications (2012-2025) on aqueous Zn-MnO_2 batteries classified by the dominant charge-storage mechanism proposed.

2) Intercalation/conversion as a main mechanism has already been conclusively ruled out in favor of the dissolution/precipitation mechanism by several publications (e.g. DOI: 10.1002/cssc.201600702; 10.1016/j.mtener.2020.100396; 10.1016/j.cej.2022.137205; 10.1039/D0EE02168G; 10.1002/anie.202318444). The mechanism is also found with Mn2O3 (10.1016/j.jpowsour.2022.231023). Although some observations are interesting, their description could be more summarized in the main text.

We sincerely thank the reviewer for highlighting this important point and for directing us to several highly relevant studies. As noted, interest in the dissolution–deposition mechanism in Zn/MnO_2 batteries has grown considerably in recent years, a trend also reflected in our overview in Figure 1. In the initial submission, we focused primarily on the most recent work

(10.1002/anie.202318444) due to space constraints, but we agree that the broader body of literature cited by the reviewer provides essential context.

Collectively, these studies trace the evolution of mechanistic understanding from early evidence of pH-coupled MnO_2/Mn^{2+} redox (10.1002/cssc.201600702), to birnessite–MnO_2/Mn^{2+} transformations (10.1016/j.mtener.2020.100396), Zn^{2+}/Mn^{2+} co-deposition and Zn–Mn compound formation (10.1016/j.cej.2022.137205; 10.1039/D0EE02168G), and more recently to frameworks involving complete dissolution and redeposition (10.1002/anie.202318444). Despite differences in the proposed pathways, these works generally interpret the charging step within a $Mn^{2+} \rightarrow Mn^{4+}$ oxidation framework.

In contrast, our combined synchrotron XAS and TEM-EELS analyses provide direct evidence that the redeposited phase does not evolve toward an Mn^{4+}-rich state. Instead, it forms as a nanocrystalline and disordered mixed-valent MnO_x containing Mn^{2+} and Mn^{3+} (Fig. 3i,j). While Mn_2O_3 (Mn^{3+} only) has previously been suggested as the deposited product (10.1016/j.jpowsour.2022.231023), that assignment relied primarily on Raman spectroscopy, which cannot unambiguously resolve oxidation state or phase identity. By integrating multiple element- and valence-sensitive probes, our study provides a more direct and constrained identification of the deposited Mn species.

Following the reviewer's suggestion, we have broadened the discussion in the revised manuscript to more clearly delineate established mechanistic interpretations from the new insights enabled by our multimodal analysis:

"While the dissolution–deposition pathway is being increasingly recognised, most studies[40–43] interpret the charging process predominantly within a $Mn^{2+} \rightarrow Mn^{4+}$ oxidation framework, often assuming the redeposited phase to be Mn^{4+}-rich (e.g., birnessite-MnO_2[41] or $ZnMn_3O_7$[43]). The oxidation state of the deposited Mn species remains challenging to establish unambiguously, as commonly used structure-sensitive or indirect probes do not uniquely resolve the valence state of the deposited products.[44]"

3) The discrepancy between experimental capacity and that expected for a dissolution mechanism has been recently studied in detail (DOI: 10.1039/D5EE03588K) ruling out the surface passivation by Zn compounds. These conclusions should be considerd and compared with the observations provided in the manuscript, which are otherwise largely confirming the other aspects reported earlier. Consequently, this section of the main text may also be shortened.

We appreciate the reviewer's point and have clarified the interpretation accordingly. Our acid-wash experiment shows that removing ZSH and other surface deposits recovers ~60 mAh g^{-1} of additional discharge capacity, indicating that surface blockage contributes a noteworthy kinetic limitation. However, the recovered capacity remains far from the two-electron limit, consistent with the recent hypothesis that initial discharge capacity - governed by dissolution - is also connectivity-dependent. Preferential removal of edge-sharing Mn–Mn units progressively enriches corner-sharing motifs, which are less reactive and ultimately limit full dissolution. We have revised and shortened the discussion to make it clear that surface

passivation is secondary, while the fundamental capacity shortfall originates from connectivity-dependent dissolution kinetics.

"Although the dissolution-deposition mechanism of MnO_2 is now relatively well established, an important question remains: why does the specific capacity of α-MnO_2 (only ~182 mAh g^{-1}) fall well below the two-electron theoretical capacity for dissolution reaction (616 mAh g^{-1})? Because the dissolution–deposition pathway is surface-mediated, the accessibility of electrochemically active surface area is expected to strongly influence the overall extent of reaction. Operando XRD (**Fig. 2a**) shows substantial formation of ZSH during discharge, which only partially dissolves upon charging. Given its insulating nature, the accumulation of ZSH on the electrode surface restricts access to reactive sites and slows dissolution–deposition kinetics. Consistent with this, acid washing to remove ZSH from the discharged alpha-MnO_2 electrode yields an additional 62 mAh g^{-1} in the following discharge (**Fig. 2c**), confirming that surface deposits impose a substantial kinetic barrier. However, the electrode still delivers far less than the two-electron capacity. Beyond surface accessibility, this limitation can be explained by the connectivity-dependent hypothesis,[33] where electron transfer is more favorable in edge-sharing than corner-sharing Mn–Mn sites. Preferential removal of edge-sharing octahedra during dissolution enriches corner-sharing Mn units at the surface, ultimately suppressing further dissolution and available capacity."

4) In line 354 ICP-OES data indicates steady increase of Mn2+ in solution. The discussion is not clear. If deposited MnO2 only partially dissolve why Mn2+ increases in electrolyte? why Mn2+ increases solution should become electrochemically inactive?

We thank the reviewer for this important point. Although the deposited MnO_x does not dissolve fully during discharge, the parent α-MnO_2 continues to dissolve over prolonged cycling, as evidenced by the progressive appearance of low-valence features in FY-mode Mn L-edge spectra. This continual consumption of pristine MnO_2 contributes fresh Mn^{2+} to the electrolyte. At the same time, residual MnO_x and ZSH persist on the surface and block active sites, limiting the extent of Mn^{2+} redeposition. Consequently, Mn^{2+} accumulates in the electrolyte as an electrochemically inactive species. We have clarified this point in the manuscript and streamlined the argument.

"With progressive cycling, the parent α-MnO_2 continues to dissolve, even as the capacity becomes increasingly dominated by redox reactions of MnO_x, leading to a gradual depletion of the parent MnO_2 phase. Consistent with this, extended cycling (20 cycles, **Fig. 3h**) leads to prominent low-valence features in both charged and discharged states - more pronounced after charge - indicating a progressive transformation of the crystalline electrode into disordered MnO_x and its accumulation on the surface. This underscores a limitation of dissolution–deposition electrochemistry: it is not fully reversible, and thus parent MnO_2 is continuously consumed over long-term cycling. Complementary ICP–OES analysis (**Fig. S36**) reveals a steady rise in Mn^{2+} concentration in the electrolyte after charging, signifying the accumulation of electrochemically inactive Mn^{2+} species that fail to redeposit. Collectively, this behavior arises from ongoing dissolution of the parent MnO_2 alongside progressive surface blockage by

residual MnO_x and ZSH, which restrict accessible sites and kinetically hinder Mn^{2+} redeposition."

5) Likewise, an excessive Mn2+ is found at early stages of discharge, at amounts that cannot be explained by an electrochemical process (line 368). The provided interpretation based on intercalation seems confusing.

We thank the reviewer for raising this important question. We agree that it may seem conceptually inconsistent to link a non-faradaic dissolution process with an intercalation mechanism. To clarify, in our manuscript, we do not attribute the early Mn dissolution to 'intercalation'; rather, we ascribe it to 'surface protonation'.

Based on our in-situ pH measurements (Fig. 4d), protons accumulate at the MnO_2 surface at the onset of discharge, leading to a pronounced local pH drop. DFT and molecular dynamics simulations (Figs. 4e,f) show that these protons preferentially adsorb on surface sites instead of entering the bulk lattice. Increasing proton coverage weakens and elongates Mn–O bonds, making the surface more susceptible to bond breaking and initiating Mn dissolution. This surface protonation therefore serves as a preconditioning step that triggers non-faradaic Mn^{2+} release during the early discharge stage.

To further support this interpretation, applying a small cathodic bias (–25 mV vs OCV for 10 minutes), chosen to avoid faradaic currents, produces a measurable increase in Mn^{2+} concentration in the electrolyte (Fig. 4g). This confirms that proton accumulation alone—without charge transfer—can promote MnO_2 dissolution. After some dissolution occurs, the resulting defective and under-coordinated surface can accommodate additional proton adsorption and even near-surface proton insertion, consistent with the calculated increase in insertion voltage for $x > 0.25$ (Fig. S38).

Taken together, we intend to emphasize that surface protonation and near-surface proton insertion are distinct processes. The former drives early non-faradaic dissolution, while the latter becomes possible only after interfacial reconstruction. To avoid conflating these two proton-driven events, we have revised the manuscript as follows to clearly separate them

"Thus, surface protonation acts as a preconditioning step that facilitates Mn–O bond scission and triggers Mn dissolution. The resulting defective and under-coordinated surface further facilitates proton adsorption and even near-surface insertion, rationalizing the calculated step-up in insertion voltage for $x > 0.25$. Notably, this later near-surface insertion is distinct from the initial protonation event that drives non-faradaic dissolution."

6) line 468 mentions "proton de-intercalation from MnO2". The chemical formula should contain H, or residual presence of protons mentioned.

We thank the reviewer for pointing this out. We have updated the chemical formula as H_xMnO_2 in the manuscript.

7) sentence at line 486 "...with first evaluated with a moderate-low active loading..." should be checked

We thank the reviewer for bringing this to our attention. We have corrected “moderate-low” to “moderate”.

Reviewer #3 (Remarks to the Author):

1. The application of EIS–DRT analysis to deconvolute kinetic contributions is a strong aspect of this work. In the in situ EIS measurements, the manuscript indicates that the cell was rested for 3 h prior to impedance acquisition at each selected point. Although such a rest period may help the system approach a quasi-equilibrium condition, it also raises questions regarding how closely the measured impedance reflects the actual electrochemical state reached during galvanostatic discharge.

After a 3 h rest, the equilibrium potential may deviate significantly from the instantaneous discharge potential, particularly in a system involving Mn dissolution and interfacial re-equilibration. Providing the full E–t evolution during the discharge–rest–EIS sequence would help clarify how representative the impedance measurements are of the preceding electrochemical process. In addition, during such extended rest periods, self-discharge and spontaneous Mn dissolution are difficult to avoid and may contribute to the observed impedance response. A brief discussion of how these effects are considered, or how the results might differ if impedance were acquired immediately after a fixed discharge time or under potentiostatic conditions, would improve confidence in the interpretation of the EIS data and the subsequent DRT analysis.

At the same time, the attribution of specific DRT (P1 and P2 area) features to proton-related surface processes or to dissolution–redeposition pathways is inherently non-unique, as similar time constants may arise from changes in interphase resistance, porous transport, or surface morphology during cycling. Framing the DRT results with a clearer discussion of these possible overlaps, or illustrating how the observed features respond to controlled variations in electrochemical or material parameters (such as Mn^{2+} *concentration, electrode thickness, or electrolyte composition), would help readers better appreciate the physical meaning and limitations of the DRT-based assignments.*

We thank the reviewer for this insightful question. We agree that in the MnO_2-Zn system, dissolution and interfacial re-equilibration can occur during resting, potentially altering the interfacial kinetics and thus the impedance/DRT response.

To evaluate this, we first quantified whether the 3-hour rest period introduces significant self-discharge. Using a symmetric MnO_2//MnO_2 cell, we determined the double-layer capacitance ($C_{dl} \approx 7.6$ mF cm^{-2}; **Figs. R2a** and **R2b**) and used the measured dE/dt during rest – at three different SOC states, i.e., at the beginning, mid-point, and fully discharged (as labeled in **Fig. R2c** and shown in **Figs. R2d**, **R2e** and **R2f**, respectively) – to estimate self-discharge currents, which were found to be several orders of magnitude smaller than the applied discharge current. This indicates that the system reaches a quasi-equilibrated state with minimal faradaic drift before EIS acquisition.

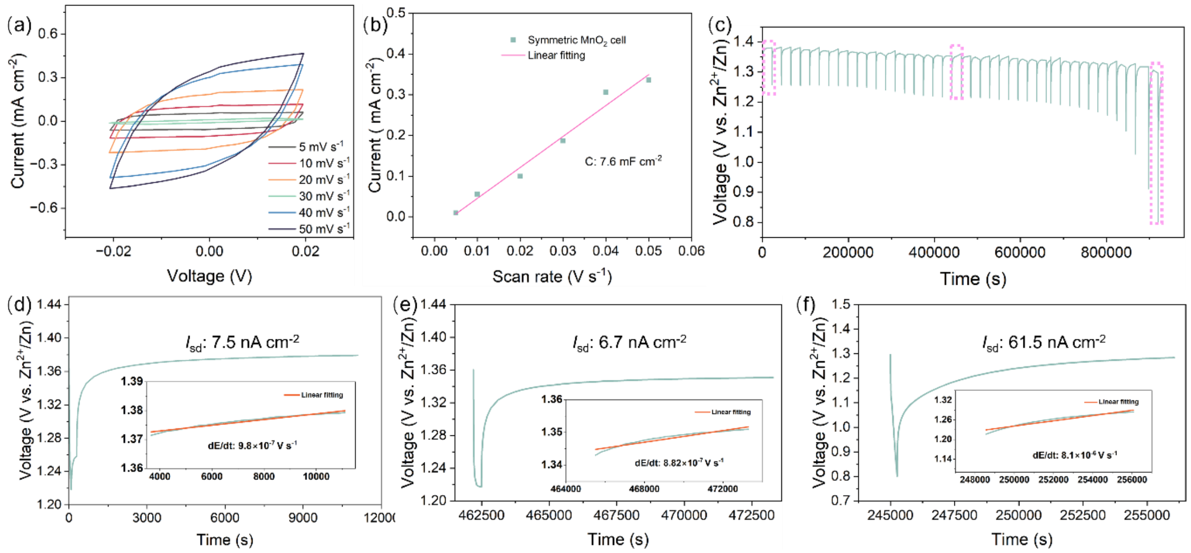

Fig. R2. (a) Cyclic voltammograms of a symmetric MnO_2 cell collected at scan rates of 5–50 mV s^{-1}. (b) Linear fit of capacitive current versus scan rate used to estimate the capacitance (C_{dl}). (c) Potential – time (E-t) polarization profile for the Zn-MnO_2 cell for the in-situ EIS testing; the highlighted regions indicate the representative steps used for self-discharge analysis. (d–f) Representative voltage-time profile at different states of discharge, with insets showing the linear fits used to extract dE/dt.

Interfacial re-equilibration will still occur during resting, including relaxation of concentration gradients, interfacial pH readjustment, and redistribution of dissolved Mn^{2+}. These processes may still have an impact on the time constants and the relative impedance of the processes (or peaks) deconvoluted by the DRT analysis. To directly examine how the relaxation time influences the DRT components, we performed control experiments using a short rest period (30 s; **Fig. R3a**). The DRT spectra under this condition still exhibit the same two-peak structure (**Fig. R3b**), but with reduced peak resolution and lower P1 intensity, a consequence of the system not yet reaching a linear, time-invariant regime. In contrast, the 3-hour rest period yields more stable and better-resolved spectra (**Fig. R3c**), enabling reliable peak attribution. Importantly, the qualitative evolution of P1 and P2 is consistent under both protocols, supporting the robustness of the mechanistic interpretation.

Regarding peak attribution, we agree that multiple interfacial processes may contribute. To strengthen the assignments, first we repeated the SOC-resolved EIS–DRT measurements under identical conditions (3 h resting) with Mn^{2+} added in the electrolyte (0.2 M $MnSO_4$ additive in 1 M $ZnSO_4$ baseline electrolyte). As shown in **Fig. R3d**, the presence of Mn^{2+} in the electrolyte selectively suppresses the P2 peak while leaving P1 largely unchanged, indicating that P2 is sensitive to the background Mn^{2+} concentration or associated with the dissolution-deposition charge transfer route. Because the spectra are collected after rest at each SOC, the DRT features reflect small-signal interfacial kinetics around the open-circuit potential rather than the net galvanostatic flux. Under these quasi-steady conditions, increasing Mn^{2+} activity raises the exchange current density of the MnO_2/Mn^{2+} interfacial redox and therefore lowers the associated charge-transfer resistance. As a result, the mid-frequency P2 feature - assigned to dissolution–deposition coupled charge transfer - diminishes in magnitude in the

$MnSO_4$-containing electrolyte. This does not imply that net dissolution is accelerated during discharge; rather, around the resting potential the interfacial reaction is easier to polarize because the solution-side reactant is more available.

In contrast, P1 remains largely unchanged because the proton-driven surface process has a weak dependence on Mn^{2+} activity. To further probe the effects of porosity and surface area, we performed SOC-EIS testing on calendered electrodes at the same mass loading to reduce porosity and accessible surface area. As shown in **Fig. R3e**, both P_1 and P_2 increase after calendering, consistent with both processes being surface-linked.

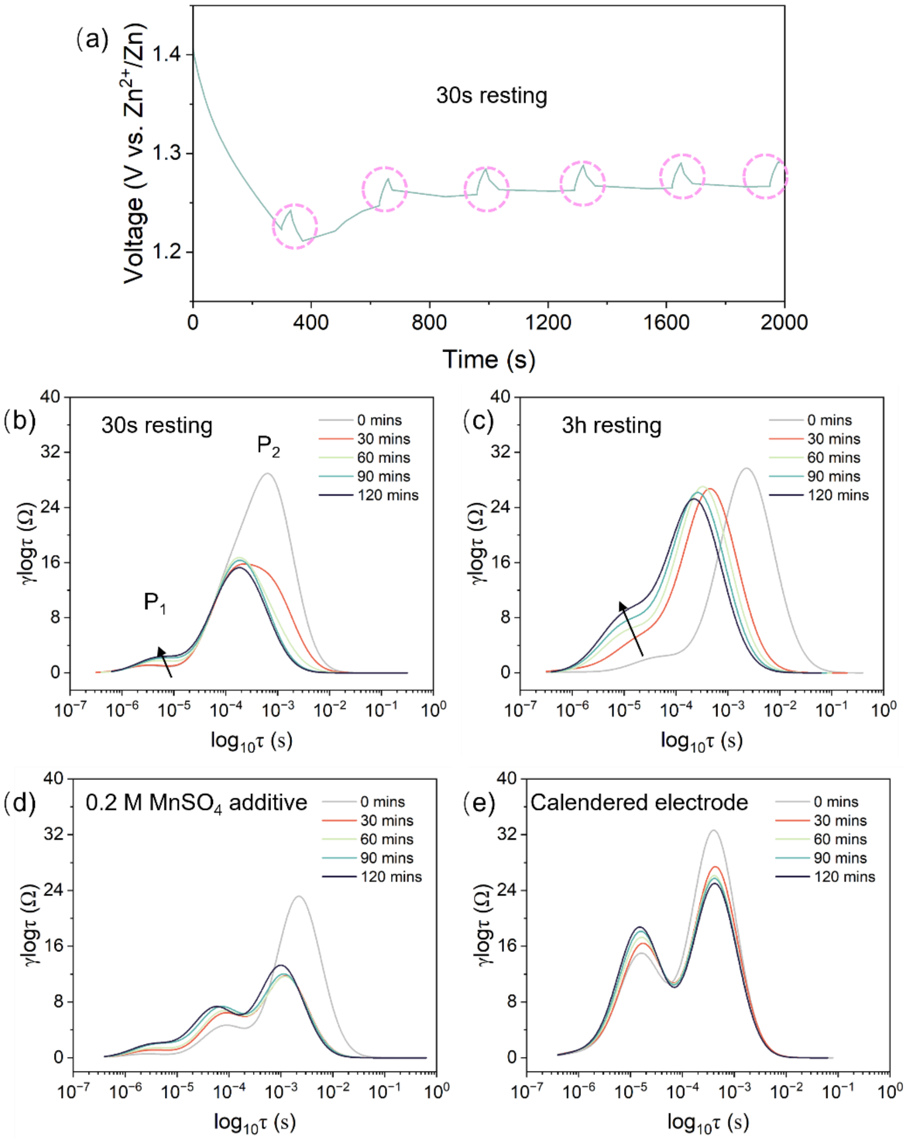

Fig. R3. (a) Voltage-time plot for 30s-resting protocol during SOC-dependent EIS testing, with dashed circles highlighting the voltage evolution during the resting period. DRT spectra corresponding to the SOC-dependent impedance data extracted after resting (b) 30 s and (c) 3 h. DRT evolution with 3h resting for (d) the electrolyte containing 0.2 M $MnSO_4$ additive and (e) calendered electrode with the baseline $ZnSO_4$ electrolyte.

Here is how we have updated the manuscript and Supporting information to address the reviewer's comment:

To more rigorously assign these peaks, we tuned Mn^{2+} activity and interfacial accessibility by adding 0.2 M $MnSO_4$ and by calendering the electrode, respectively. Increasing Mn^{2+} selectively suppressed P2 while leaving P1 largely unchanged, whereas calendering enhanced both peaks. These trends indicate that P1 originates from a proton-driven surface process, while P2 reflects dissolution–deposition charge-transfer kinetics (see **Fig. S42** for detailed discussion). Notably, P2 is pronounced at the onset of discharge, consistent with a sizable kinetic barrier for initial Mn–O bond scission.

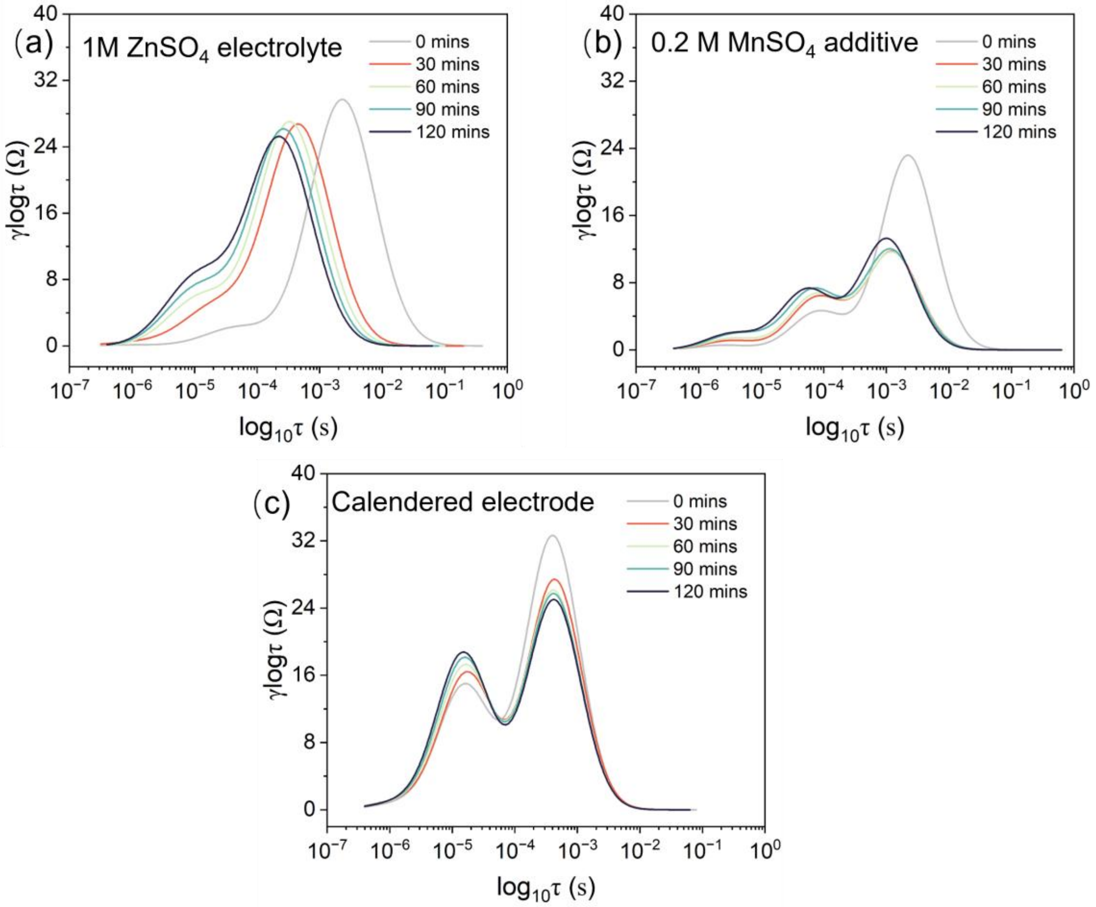

Fig. S42. SOC-resolved EIS–DRT data collected during MnO_2 discharge with (a) 1M $ZnSO_4$ electrolyte, (b) 1M $ZnSO_4$ electrolyte containing 0.2 M $MnSO_4$ additive and (c) calendered electrode and 1M $ZnSO_4$. Adding $MnSO_4$ selectively suppresses the mid-frequency P2 feature while leaving the high-frequency P1 largely unchanged. Because spectra are acquired after rest at each SOC, the peaks reflect small-signal interfacial kinetics around the open-circuit potential. The lower P2 amplitude with Mn^{2+} arises from a higher exchange current density of the MnO_2/Mn^{2+} interfacial redox, which reduces the charge-transfer resistance of the dissolution–deposition step; this should not be interpreted as accelerated net dissolution during galvanostatic discharge. Calendering increases both P1 and P2, consistent with reduced porosity and lower interfacial accessibility amplifying surface- and charge-transfer-related polarization. Taken together, these trends support assigning P1 to a proton-driven surface process and P2 to dissolution–deposition coupled charge transfer.

2. Throughout the manuscript, the redeposited phase is described as "Mn_3O_4-like," mainly based on average Mn valence and electrochemical characteristics. While this interpretation is reasonable, it is worth noting that Mn redeposition in aqueous systems often leads to structurally disordered, hydrated, or mixed-valence MnO_x phases, especially after extended cycling. Placing this discussion in the context of recent studies that combine long-term structural analysis with techniques such as solid-state NMR would help clarify whether a Mn_3O_4-like description is uniquely supported in the present case, or whether a more general description of mixed-valent MnO_x would be more appropriate (for example, see recent analyses reported in 10.1021/acsenergylett.4c02559).

We thank the reviewer for this insightful comment. We agree that caution is needed when comparing the deposited MnO_x to crystalline Mn_3O_4. In our manuscript, we do not assign the deposited phase as Mn_3O_4, nor do we refer to it as such. Rather, we report that the average Mn oxidation state extracted from L-edge XAS lies close to that of Mn_3O_4, which motivated electrochemical comparisons, but we consistently describe the deposited material as simply MnO_x or mixed-valent MnO_x.

High-resolution TEM and synchrotron XRD confirm that the deposited phase is nanocrystalline and highly disordered, preventing an unambiguous structural assignment. The recent study highlighted by the reviewer, which reports the formation of crystalline Mn_3O_4 after extensive long-term cycling, provides useful context, and we now cite it in the revised text. However, our measurements focus on early-cycle behavior, where the deposited material cannot be structurally classified. Accordingly, to emphasize this clarification and avoid possible misunderstanding, we have revised the discussion in the manuscript and Supporting Information as follows.

"Notably, although the deposited product exhibits an average Mn oxidation state and electrochemical signature similar to Mn_3O_4, and a prior study has reported the formation of spinel Mn_3O_4 after long-term cycling,[54] its nanocrystalline disordered character, as observed in this study, precludes an unambiguous assignment. We therefore refer to the deposited phase as MnO_x or mixed-valent MnO_x throughout and use Mn_3O_4 only as a valence benchmark for comparison."

"Such transformation indicates a likely mechanistic pathway, where the local interfacial pH becomes relatively high and hence fosters local Mn^{2+} to transiently form a metastable $Mn(OH)_2$, which is electrochemically converted to mixed-valent MnO_x."

"**Fig. S34.** The linear combination fitting of Mn L-edge AEY spectrum for α-MnO_2 electrode after (a) 1st discharge (b) 1st charge. Although the fitted average Mn valence of the deposited material is close to that of Mn_3O_4, this does not imply a spinel structure; TEM and XRD indicate an amorphous/disordered MnO_x."

"**Fig. S48**. The GCD profile of Zn-Mn_3O_4 battery for the first two cycles while cycling at 100 mA g^{-1} in the range of 0.8- 1.8 V vs. Zn^{2+}/Zn. These data are presented to benchmark electrochemical signatures of Mn_3O_4 against those observed for the deposited MnO_x. This comparison does not imply that the deposited phase is crystalline Mn_3O_4."

3. The detection of Mn^{2+} at early stages of discharge is an intriguing observation that supports the proposed reaction pathway. However, Mn dissolution can also occur during rest or originate from residual surface species, making it important to clearly distinguish electrochemically driven dissolution from spontaneous chemical processes. For example, add D0 point in Fig. 4a. Clarifying whether similar Mn^{2+} release is observed under open-circuit conditions, or briefly discussing relevant control experiments, would help reinforce the interpretation that the early-stage dissolution observed here is intrinsically linked to the electrochemical process.

We thank the reviewer for this important point. Distinguishing spontaneous chemical dissolution from electrochemically promoted dissolution is indeed essential. Our initial resting experiment (Fig. 4g) showed that some Mn^{2+} appears during rest, but we have now performed additional controlled resting tests (30 min, 1 h, 2 h). As shown in Fig. R4, Mn^{2+} release at open circuit stabilizes at ~2.9% of the MnO_2 mass after 1 h, which remains far below the ~10% observed at the early-discharge D1 state. This demonstrates that most of the Mn dissolution observed at D1 is not spontaneous but arises from electrochemical polarization and the resulting interfacial proton accumulation.

These results support our interpretation that surface protonation under bias - rather than spontaneous disproportionation - initiates the non-faradaic dissolution during the early stage of discharge. We have clarified this distinction in the revised manuscript.

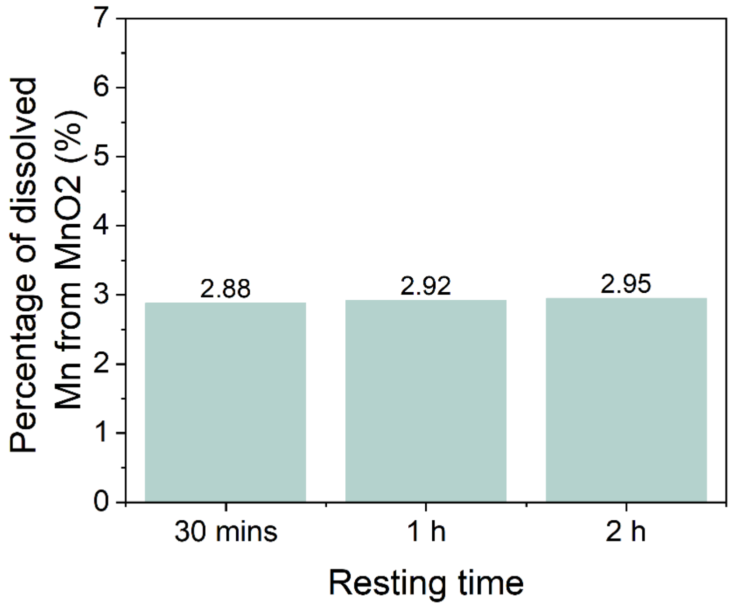

Fig. R4. Quantification of dissolved Mn^{2+} in the electrolyte (normalized to active material mass) at different resting time.

Here is how we have addressed this comment in the revised manuscript.

"In contrast, before cycling (D0, after 1 h resting), only about 2.9% of MnO_2 dissolves, indicating that spontaneous chemical dissolution contributes only a minor fraction relative to the early-discharge dissolution (D1)."

4. The manuscript emphasizes proton involvement and surface proton priming as key elements of the reaction mechanism. This discussion would benefit from being more explicitly situated within the broader context of recent work on proton insertion and H_xMnO_2 formation in α-MnO_2-based cathodes. In particular, recent studies employing techniques such as EQCM-D in combination with solid-state NMR have provided direct insight into proton insertion behavior and the stabilization of H_xMnO_2 phases. Discussing how the present findings align with, extend, or differ from these observations (e.g., 10.1002/smll.202404583) would further strengthen the mechanistic framing and highlight the contribution of the current work.

We thank the reviewer for this valuable suggestion and the relevant reference. The cited study provides strong evidence for proton insertion in MnO_2-based systems (including 2H ssNMR and EQCM-D), and we now note and refer to in the revised manuscript.

In our system, with mildly acidic zinc sulfate electrolytes, it is revealed that proton-related redox is predominantly near-surface in the parent MnO_2 and becomes progressively accommodated by the redeposited MnO_x nanosheets with cycling, rather than occurring as bulk insertion into MnO_2. This is supported by our depth-resolved valence analysis. Our findings therefore complement the cited work by clarifying that, under these conditions, proton insertion does not occur as bulk intercalation into MnO_2 but instead proceeds through a predominantly near-surface pathway. We have added this contextual clarification to the manuscript.

"Notably, a recent study has also confirmed the proton insertion through ^2H solid-state NMR and Electrochemical Quartz Crystal Microbalance Study.[39] However, as we observe, only a very small fraction of the first-discharge capacity arises from the non-dissolution pathway (vide supra), indicating that H^+ insertion in the parent α-MnO_2 is limited and predominantly surface-mediated, rather than occurring as bulk intercalation as proposed in that study."

Response to Reviewers

We are grateful to the reviewers for their time in assessing our manuscript and providing a critical assessment. Here, we are providing a detailed response to each comment. Reviewers' comments and suggestions are *italicized*, and our answers are in blue for clarity. Any changes in the manuscript are highlighted in yellow.

Reviewers #1 and #3:

We thank Reviewer #1 and Reviewer #3 for their positive assessments and for recommending the manuscript for publication. We appreciate their careful evaluation and are pleased that the revisions addressed their previous concerns.

Reviewer #2 (Remarks to the Author):

The authors maintained the extensive structure of their manuscript, although they did a significant effort to take specific comments into consideration. However, some points are still not satisfactorily addressed.

We are thankful to the reviewer for the careful assessment. Based on the reviewer's questions and comments, we have made targeted revisions/edits in the manuscript, as noted in our point-by-point response below, while preserving the comprehensiveness needed to support the multimodal analysis of MnO_2's complex charge storage mechanism. We hope the revisions have further tightened the narrative and clarified the mechanistic arguments without overclaiming the study's reach.

3) In spite of the fact that it is now recognized at page 8 that also other passivating mechanisms can be responsible for the MnO2 passivation, ZSH is still repeatedly indicated as the main responsible for kinetic and capacity limitation (lines 207, 367, 411, 446, 487, 548, 568). It is certainly difficult to discriminate which specific passivation effect is responsible for the hindered dissolution, but direct evidence for the ZHS alone does not seem strong enough. A more generic "passivation" term seems more correct.

We thank the reviewer for this important point. In the revised manuscript, we have systematically updated all ZSH-related statements to avoid implying that ZSH alone governs kinetic or capacity limitations. ZSH is now framed as one significant component of a broader interfacial passivating layer that also includes residual MnO_x and ZSH-derived insulating byproducts. These revisions ensure that we do not over-assign causality to ZSH and instead refer to "interfacial passivation" or "passivating deposits," in line with the reviewer's recommendation. These changes have been applied consistently throughout the manuscript.

Here are some of the corresponding edits in the revised manuscript:

Line 214-216: "Given its insulating nature, ZSH contributes to interfacial passivation and restricts access to reactive Mn–O sites, thereby slowing dissolution–deposition kinetics."

Line 218-225: "Notably, ZSH can also transform into other insulating byproducts, such as poorly ordered ZnO,[33] which is difficult to resolve unambiguously by XRD or other spectroscopic techniques; such species would also contribute to interfacial passivation. Beyond

surface accessibility, the capacity limitation may also stem from the local atomic structure in MnO_2, as has been proposed earlier.[33] In particular, electron transfer is more favorable in edge-sharing than corner-sharing Mn–Mn sites. Preferential removal of edge-sharing octahedra during dissolution enriches corner-sharing Mn units at the surface, ultimately suppressing further dissolution and available capacity."

4) it is unrealistic that during several cycles the contribution of parent MnO2 to capacity continues to be relevant. It remains unclear how Mn2+ in solution can become electrochemically inactive. Its accumulation must be rather related to increased difficulty of MnOx to form. The accumulation of insulating components on the electrode might be one possible cause.

We appreciate the reviewer's comment. We agree that the contribution of the parent MnO_2 to the observed capacity does not remain significant beyond the early cycles. As we have noted in the "Deterioration of dissolution–deposition reversibility" and "Mechanistic mapping" sections, while the parent MnO_2 progressively transforms into disordered MnO_x during cycling, the reversible capacity becomes increasingly dominated by the deposited MnO_x phase.

Regarding the increase in Mn^{2+} concentration in the electrolyte, the revised text emphasizes that this accumulation does not imply that Mn^{2+} becomes intrinsically inactive. Rather, as stated in the manuscript, progressive surface blockage by residual MnO_x and ZSH-related passivating species limits the accessibility of nucleation sites for Mn^{2+} redeposition. In parallel, continued dissolution of the shrinking MnO_2 framework supplies fresh Mn^{2+} into solution. These two effects together account for the observed rise in solution-phase Mn^{2+} without invoking any loss of intrinsic electrochemical activity.

Below is the corresponding revision in the revised manuscript.

Line 365-372: "This underscores a limitation of the dissolution–deposition electrochemistry: it is not fully reversible. Complementary ICP-OES analysis (**Fig. S36**) reveals a steady rise in Mn^{2+} concentration in the electrolyte after charging with cycling. This does not indicate that Mn^{2+} becomes intrinsically inactive; rather suggests that the progressive accumulation of residual MnO_x and ZSH-related insulating byproducts on the surface reduces the number of accessible nucleation sites for redeposition. In parallel, the shrinking MnO_2 framework continues to supply Mn^{2+} into solution over successive cycles, while the elevated Mn^{2+} concentration imposes a common-ion effect that suppresses further dissolution."

5) dissolution of Mn(IV) to Mn2+ needs electrons, if the process is not faradaic, what chemical species will provide them?

The reported observations are certainly intriguing, even if some of the explanations can remain partial and open.

We appreciate the reviewer's question and suggestion. In the revised manuscript, we have added a plausible explanation for non-Faradaic dissolution. As our DFT and MD simulations show, proton adsorption weakens Mn–O coordination. Under such proton-primed conditions, surface and sub-surface layer Mn^{3+} likely undergoes disproportionation or short-range internal

electron exchange with Mn^{4+} centres, which provide a plausible pathway for supplying the electrons required to form Mn^{2+} before any measurable faradaic current is passed.

We have also noted, with appropriate caution, that a minor contribution from water oxidation cannot be entirely ruled out under highly protonated interfacial conditions.

Here is how we have addressed this in the revised manuscript.

Line 403-409: "A plausible explanation is that under the proton primed condition, Mn^{3+} domains in the surface and sub-surface layers undergo disproportionation or short-range internal electron exchange with Mn^{4+}, to lead to Mn^{2+} dissolution before any measurable faradaic current is passed; although our data support this pathway, we cannot entirely rule out a minor contribution from water oxidation under these highly protonated conditions, providing electrons for MnO_2 reduction."
